# Advances in CO_2_ activation by frustrated Lewis pairs: from stoichiometric to catalytic reactions

**DOI:** 10.1039/d3sc03907b

**Published:** 2023-11-09

**Authors:** Md. Nasim Khan, Yara van Ingen, Tribani Boruah, Adam McLauchlan, Thomas Wirth, Rebecca L. Melen

**Affiliations:** a Cardiff Catalysis Institute, School of Chemistry, Cardiff University, Translational Research Hub Maindy Road, Cathays Cardiff CF24 4HQ Cymru/Wales UK MelenR@cardiff.ac.uk; b School of Chemistry, Cardiff University Main Building, Park Place Cardiff CF10 3AT Cymru/Wales UK wirthT@cardiff.ac.uk

## Abstract

The rise of CO_2_ concentrations in the environment due to anthropogenic activities results in global warming and threatens the future of humanity and biodiversity. To address excessive CO_2_ emissions and its effects on climate change, efforts towards CO_2_ capture and conversion into value adduct products such as methane, methanol, acetic acid, and carbonates have grown. Frustrated Lewis pairs (FLPs) can activate small molecules, including CO_2_ and convert it into value added products. This review covers recent progress and mechanistic insights into intra- and inter-molecular FLPs comprised of varying Lewis acids and bases (from groups 13, 14, 15 of the periodic table as well as transition metals) that activate CO_2_ in stoichiometric and catalytic fashion towards reduced products.

## Abstract 2

Frustrated Lewis pair systems have been explored efficiently in homogeneous and heterogeneous conditions for the activation and reduction of CO_2_ to various useful products in stoichiometric as well as in catalytic reactions.

## Graphical abstract

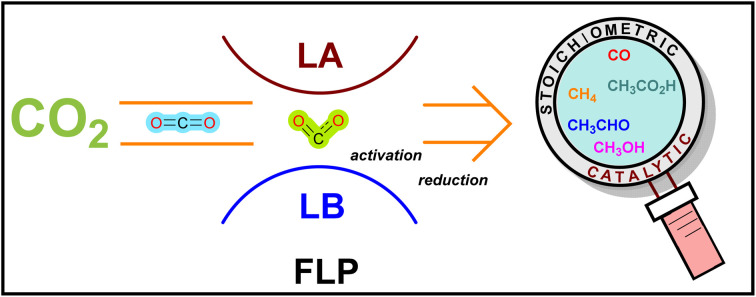

## Introduction

### Background

Since the beginning of the industrial revolution, human activity has raised the concentration of carbon dioxide (CO_2_), amongst other important greenhouse gases, in the environment by over 50%. CO_2_ gas absorbs and emits radiant energy at infrared wavelengths that causes an increase in atmospheric temperature, thus global warming and has become a main driver of climate change.^1^ Society has made some progress in finding low-carbon emitting alternatives, for example in sources of energy, and now a scattered dip in CO_2_ level has been observed. Minimising CO_2_ emissions by at least 50% to limit the increase in the global average temperature by 2 °C by 2050 has been set as a global target.^2^ This will require a rapid exploitation of new energy technologies with a low-carbon or zero-carbon energy sources to restore our ecosystem. As one single technology is not expected to solve this problem, global warming alerts have drawn urgent attention to control the expansion of CO_2_ concentrations in the atmosphere through the framework of carbon capture, storage, and utilisation (CCSU).^3^ The important challenge remains not only in carbon dioxide capture and storage but also to utilise it for the creation of value-added carbon products.^4^ CCSU processes add value to the conversion of CO_2_ into fuels and chemicals, and can compensate the cost of capturing CO_2_. This approach has generated many new directions in various branches of science and technologies including chemical, biological and material applications.^5^ Extensive work has been carried out in the past few decades for CO_2_ capture and utilisation (CO_2_-CU) using various chemical processes for the reduction of CO_2_ into products such as formic acid and methanol, or light hydrocarbons such as methane.^6^ Metal and non-metal derived reagents and catalysts in homogeneous and heterogeneous systems have been explored including promising mediums such as zeolites, metal-organic frameworks (MOFs), covalent organic frameworks (COFs), nanomaterials, as well as electrochemical, photochemical and thermal processes.

The activation and chemical conversion of CO_2_ requires high energy due to its high thermodynamic stability (
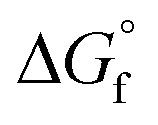
 = −396 kJ mol^−1^).^7^ Entropy is one important factor that limits CO_2_ transformations, even for some reactions where Δ*H*° < 0, Δ*G*° is positive. Conversion of CO_2_ can take place at room temperature or at lower temperatures but in such transformations the carbon atom retains its oxidation state of +4 and promotes the formation of carbamates, carbonates, urea and its derivatives, polycarbonates, and polyethers, where OH^−^, H_2_O, amines, carbanions, olefins, alkynes, and dienes have been reacted as reagents with CO_2_. Reactions that produce carboxylates from CO_2_ are thermodynamically favourable. Carbon product formation such as methanol, carbon monoxide, formaldehyde, methane, and hydrocarbons from CO_2_ require higher energy, and thus kinetic control to steer away from the thermodynamically favoured products. Kinetically, to obtain CO_2_ reduced products with a change of oxidation state in the carbon atom requires more energy. To achieve the reduction of CO_2_, the reagent should bend CO_2_ to overcome the first energy barrier and to achieve subsequent reduction steps. Preorganised reducing agents kinetically favour the challenging reduction of CO_2_ beyond carbonate or formate.

To overcome the energy barrier, external sources of energy (such as electrochemical, photochemical or thermal energy) are typically required. If value-added products are obtained at higher cost compared to the cost of CO_2_ capturing and natural fuels, then the process would not be economical and could not be applied industrially.^8^

**Fig. 1:**
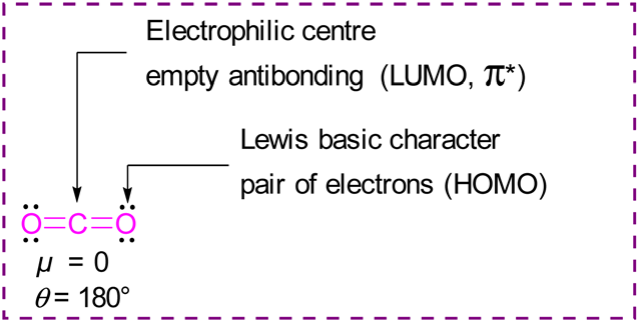
Molecular properties of carbon dioxide.

Although CO_2_ has been reduced to obtain chemicals and fuels, the current use of CO_2_ in chemical synthesis is limited owing to the high thermodynamic stability of CO_2_ that has to be overcome.^9^ To control the energy barrier in reduction processes of CO_2_, metals are being used that are often rare and high cost materials, indeed transition metals are being explored making the process efficient and cost effective. The conversion of CO_2_ to value-added products is highly desirable, yet the inert and highly energetically stable nature of this small molecule makes this a challenging task.^10^ CO_2_ is a linear and an apolar molecule (dipole moment, *μ* = 0), despite its two polar C=O bonds and is ambiphilic in nature. The length of the C=O bond in CO_2_ is 116.3 pm, shorter than the approximate 140 pm bond length of a typical single C–O bond, and shorter than most other C=O double bonds, such as carbonyls.^11^ It offers two reaction sites, an electrophilic site at the carbon (Lewis acidic centre) due to the low-lying empty antibonding π* orbital (LUMO, lowest unoccupied molecular orbital), and two nucleophilic sites at the oxygen atoms (Lewis basic character) due to an available pair of valence electrons (HOMO, highest occupied molecular orbital) (Fig. 1). Carbon dioxide has an electron affinity (*E*_ea_) of about −0.6 eV and a first ionisation potential (IP) of about +13.8 eV which makes it a better electron acceptor than electron donor. Overall, a high energy of about 750 kJ mol^−1^ is required to break the C=O bond. Upon activation, the molecule will distort from its linear sp-hybridised geometry to a sp^2^-hybridised carbon centre with concurrent elongation of the C=O bond and a change in its molecular energy.^12^

**Fig. 2:**
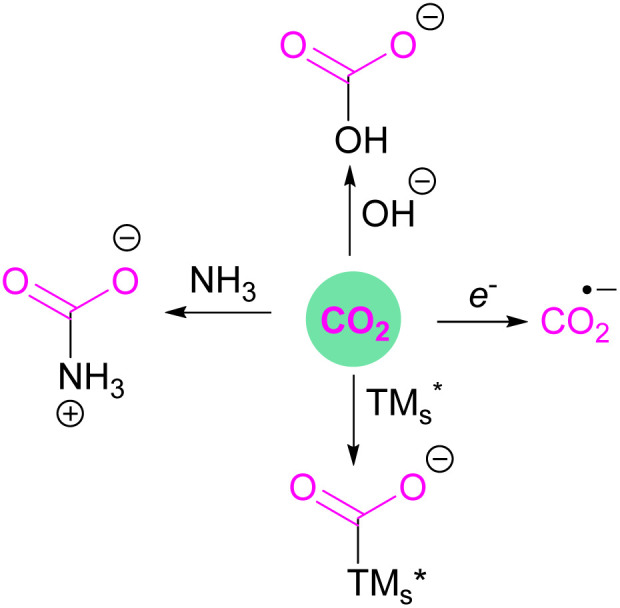
Classical activation reactions of carbon dioxide.

Classical activation of CO_2_ by nucleophilic attack at the carbon atom can be achieved using bases,^13^ transition metals,^14^ or by one electron reductions^15^ to ultimately generate acetates, carbamates, ureas, bicarbonates, oxalates, formates, or carbon monoxide, amongst other products (Fig. 2). Further advances in research have been able to achieve value added carbon products by reducing CO_2_ to products such as methanol, methane, or higher carbon chains.^16^

Frustrated Lewis pairs (FLPs)^17^ are mixtures of a Lewis acid and a Lewis base, that, because of steric hindrance, cannot combine to form a classical adduct. FLPs can perform efficient chemical transformations without losing the individual properties of the FLP system.^18^ This feature also enables the activation of small molecules including CO_2_ and is now well-explored in the literature in several reviews.^19^ Herein, we cover all major developments made using p-block elements and transition metals to activate CO_2_ with FLP systems with a particular emphasis on more recent reports. In this review we will cover stoichiometric as well as catalytic processes including theoretical efforts to understand the mechanism of CO_2_ activation and reduction using FLPs.

## Frustrated Lewis pairs (FLPs)

**Fig. 3:**
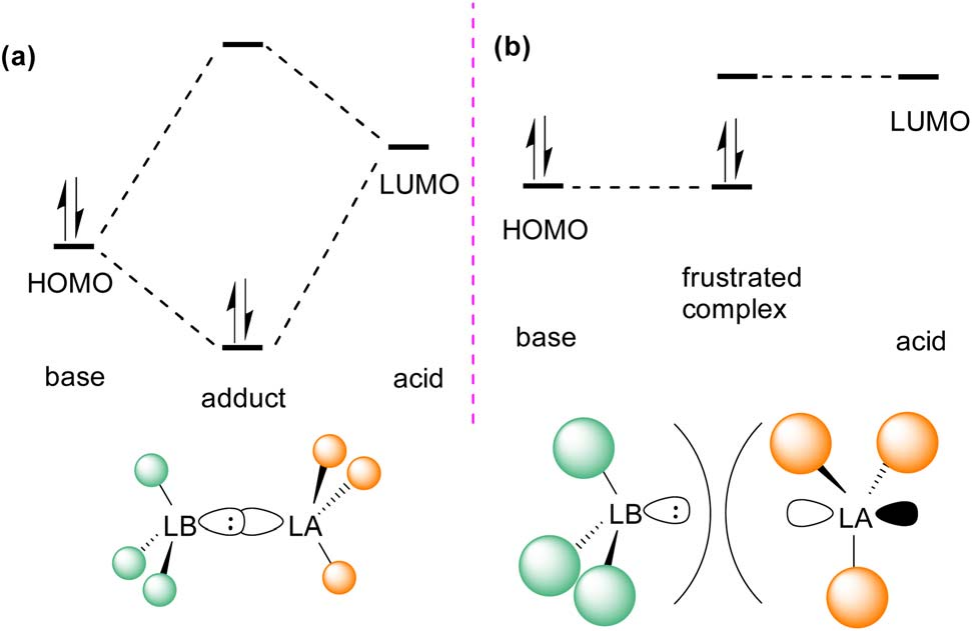
Frontier molecular orbital presentation of (a) a classic Lewis acid–base adduct and (b) a frustrated Lewis pair. LA = Lewis acid; LB = Lewis base.

In the classic model of Gilbert Lewis (Fig. 3a), a Lewis base with an electron pair in the HOMO donates electron density to the LUMO of the Lewis acid by forming a dative bond.^20^ This process provides a HOMO of lower energy with a stabilised donor acceptor adduct and quenches the reactivity of both, the Lewis acid and base. A deviation to the classical model was observed after the augmented work reported by Brown,^21^ in which no adduct formation occurred between BMe_3_ and 2,6-lutidine, and later Wittig,^22^ Tochtermann,^23^ Piers^24^ and Oestreich.^25^ Stephan and co-workers coined the chemical term “frustrated Lewis pair” (FLP) that exists with unquenched acidity and basicity in a combination of a sterically hindered Lewis acid and Lewis base (Fig. 3b).^26^ This inhibition of adduct formation allows for the HOMO of the Lewis base and the LUMO of the Lewis acid to effect non-classical reactivity.

**Scheme 1:**
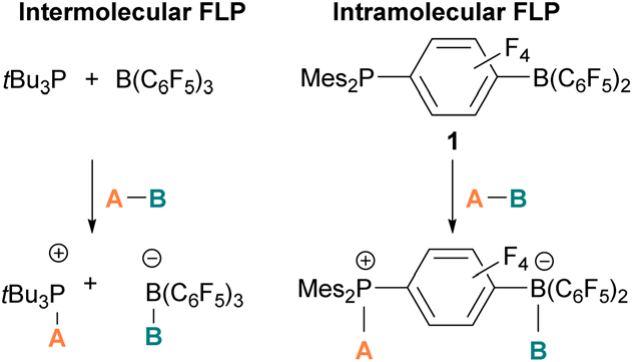
Cleavage of molecule A–B with intermolecular and intramolecular FLPs. Mes = Mesityl.

**Scheme 2:**
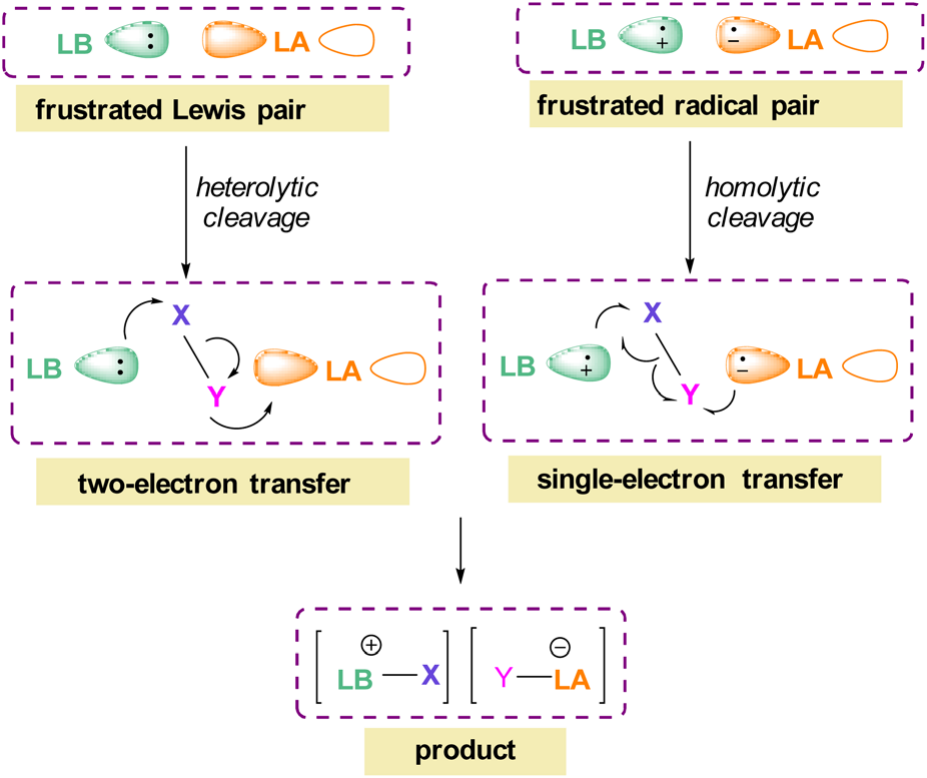
FLP reactivity with small molecules *via* heterolytic (two e^−^ transfer) and homolytic cleavage (single e^−^ transfer).

The pioneering work of splitting dihydrogen heterolytically with the FLP *t*Bu_3_P/B(C_6_F_5_)_3_ demonstrated that the unquenched reactivity of FLPs could be applied to the activation of small molecules.^27^ Since then, various FLP systems have been investigated to activate a variety of small molecules. Two types of FLP systems are typically considered: intermolecular or intramolecular (Scheme 1). Intermolecular FLPs are systems where the Lewis acid and Lewis base are two individual molecules that interact through secondary London dispersion interactions to bring the Lewis acid and base together where small molecules insert into the cavity of the FLP such as the combination of *t*Bu_3_P and B(C_6_F_5_)_3_. In intramolecular FLP systems, the Lewis acid and Lewis base are combined in one molecule by a covalent linker. An example of an intramolecular FLP system is the phoshinoborane 1 shown in Scheme 1, where the Lewis acidic boron centre and the Lewis basic phosphorus centre are separated by an aryl ring. These molecules are also able to heterolytically cleave the small molecule A–B (Scheme 1). When the FLPs heterolytically cleave the bonds in small molecules, the Lewis base donates its electron pair to the electron deficient fragment of the small molecule and the Lewis acid accepts an electron pair from the HOMO of the small molecule. This results in bond formation and an ionic/zwitterionic product. It has also been found that certain combinations of Lewis acids and bases in FLPs can lead to a transfer of one electron from the Lewis basic donor to a Lewis acidic acceptor generating a reactive frustrated radical pair (FRP). This FRP can react in a homolytic way with small molecules (Scheme 2).^28^

## Mechanistic aspects of FLP CO_2_ reduction

**Scheme 3:**
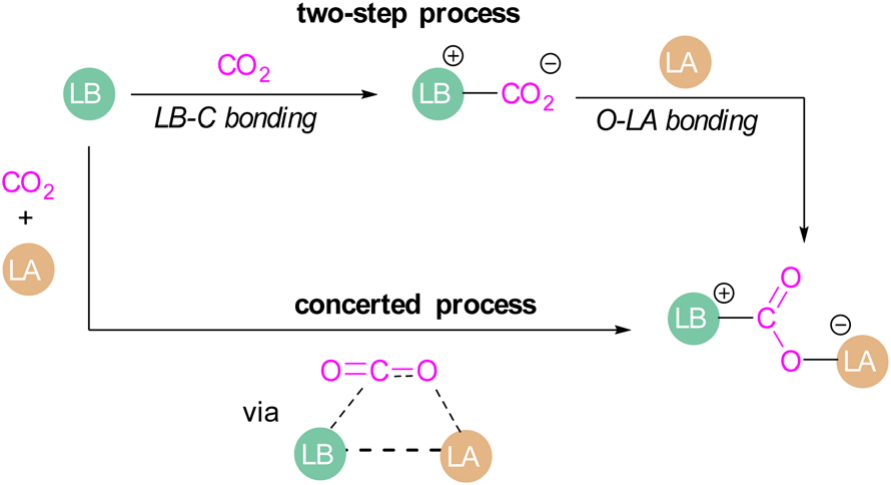
Mechanistic aspects of FLPs in their reaction with CO_2_.

Amongst the small molecules activated by FLPs, CO_2_ has been well-studied owing to the importance of discovering new CCSU processes. Two mechanistic pathways are proposed for the capture and activation of CO_2_ by FLPs thus far (Scheme 3). One is a concerted mechanism and the other is a two-step process. Two computational models have been explored in the *t*Bu_3_P/B(C_6_F_5_)_3_ FLP system to study the mode of CO_2_ activation. In the concerted mechanism, the reactants (FLP and free CO_2_) and the CO_2_-FLP adduct is formed by a single transition state (TS) in which the LB–C and LA–O bonds are formed simultaneously.^29^ Conversely, for the two-step process, when the CO_2_ moves closer to the FLP system, the P–C bond is formed first, followed by the formation of the B–O bond to give the final CO_2_-FLP adduct.

It is well-documented that the solvent is important to stabilise the final zwitterionic products.^30^ Liu and co-worker^31^ led a mechanistic study in the solid-state utilising density functional theory (DFT) simulations, in which they analysed the separate roles of the Lewis acid and base without the presence of a solvent. The authors found that the reaction proceeds in a two-step process where CO_2_ initially enters the cavity of the *t*Bu_3_P/B(C_6_F_5_)_3_ FLP. The carbon atom of CO_2_ then interacts with the phosphorus atom and an oxygen atom interacts with boron leading to a reduction in the O–C–O angle of CO_2_ to 167.8°. This means that the CO_2_ species is bent although there are no chemical bonds formed. They believe that this is due to a weak interaction between CO_2_ and the FLP, where CO_2_ interacts with crystal fields in the solid state created by the FLP pair. In the solution state, the crystal fields would be replaced by solvent interactions. In studying the separate roles of the Lewis acid and base, the authors suggest that the combination of a strong Lewis acid and a weak Lewis base should be selected to make the CO_2_ activation thermodynamically feasible. This is due to the formation of the B–O bond being strongly exergonic while the formation of the P–C bond was deduced to be endergonic.

Other sources of energy such as light and/or electric current have been employed in other fields however, the most used source of energy for the activation of CO_2_ with FLP systems is heat and pressure of CO_2_.

**Scheme 4:**
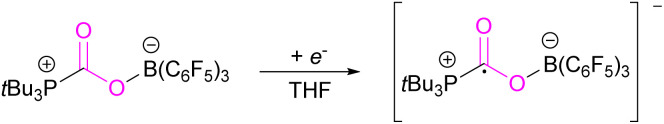
Electrochemical reduction of FLP adduct *t*Bu_3_P-CO_2_-B(C_6_F_5_)_3_.

However, in the FLP adduct of CO_2_, the CO_2_ molecule is bent, and a one-electron transfer could facilitate the reduction process. In a homogeneous system, the first electrochemical study was performed on the FLP-CO_2_ adduct for *t*Bu_3_P-CO_2_-B(C_6_F_5_)_3_ (Scheme 4).^32^ Electrochemically, when an electron is added to *t*Bu_3_P-CO_2_-B(C_6_F_5_)_3_, a change in the bond lengths was observed. The carbon oxygen C=O bond length increased by 0.05 Å in and the C–O bond length decreased by 0.03 Å. The B–O and P–C bonds both decreased in length by 0.06 Å and 0.01 Å, respectively. Overall, it was observed that addition of electrons to the CO_2_ adduct *t*Bu_3_P-CO_2_-B(C_6_F_5_)_3_ first generated intermediate [*t*Bu_3_P-CO_2_-B(C_6_F_5_)_3_]^−^, then reduced CO_2_ to CO also generating *t*Bu_3_P, and [(HO)B(C_6_F_5_)_3_]^−^. In this system, the intermediate [*t*Bu_3_P-CO_2_-B(C_6_F_5_)_3_]^−^ can also react with the solvent THF causing dimerisation.

To obtain a controlled reduction of CO_2_, FLPs in the solid-state have been explored based on the phenomenon of adsorption, activation, and evolution pathways of CO_2_. For example, Yan and co-workers created a stable FLP system for the activation of carbon dioxide.^33^ Their system involves a composite material of zinc and tin having different electronegativities. The Lewis pairs first capture and stabilise protons and then selectively activate CO_2_. Here the zinc oxide surface captures protons and acts as a Lewis base while the tin acts as Lewis acid. The two-electron reduction with two protons start the reaction for CO_2_ activation and finally resulted in the formation of formic acid.

In this review we will cover FLP mediated activation and reduction of CO_2_ that has been explored to achieve value-added carbon products. This will include, several inter- and intramolecular FLPs systems which have been designed and developed utilising both p- and d-block elements acting as a Lewis acid in combinations with p- and d-block elements acting as a Lewis base. Each section and sub-sections will detail the different systems developed with stoichiometric and catalytic quantities of FLPs, and will be ordered by the periodic group of the Lewis acid to give structure to this review. The mechanistic and computational insights will be discussed where relevant.

## Group 13 Lewis acids

### Borane/phosphine FLPs for CO_2_ activation

Boron is by far the most explored Lewis acidic element in FLPs for CO_2_ activation. Different Lewis base partners such as phosphrous, nitrogen, carbon as a carbene and metals have been explored in combination with the boron Lewis acid.

**Scheme 5:**
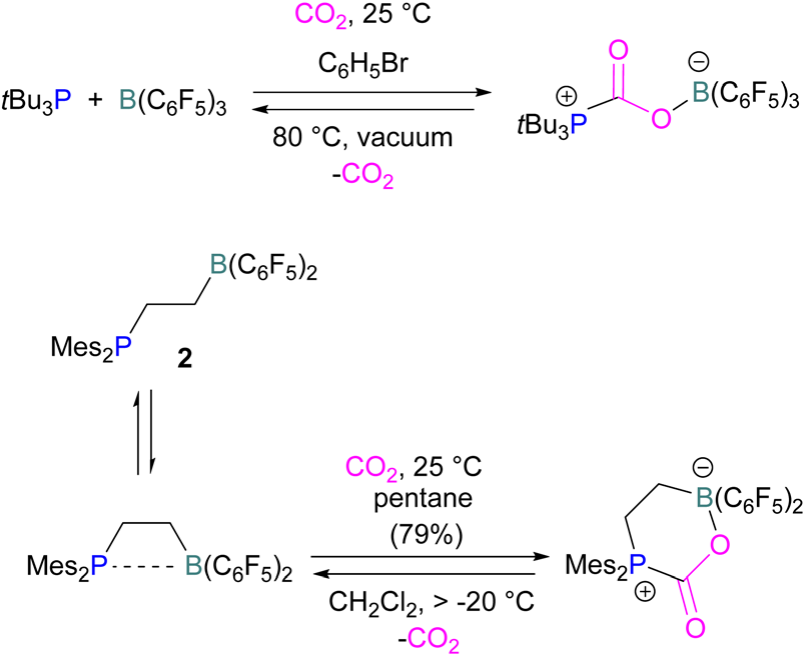
First inter- and intramolecular FLPs utilised in CO_2_ activation.

Many of the first FLP systems for CO_2_ activation involved phosphorus as the Lewis basic component. Early FLPs utilised in CO_2_ activation comprised of a phosphine and borane that could reversibly bind and release CO_2_ including the intermolecular FLP *t*Bu_3_P/B(C_6_F_5_)_3_ and 2 (Scheme 5).

**Fig. 4:**
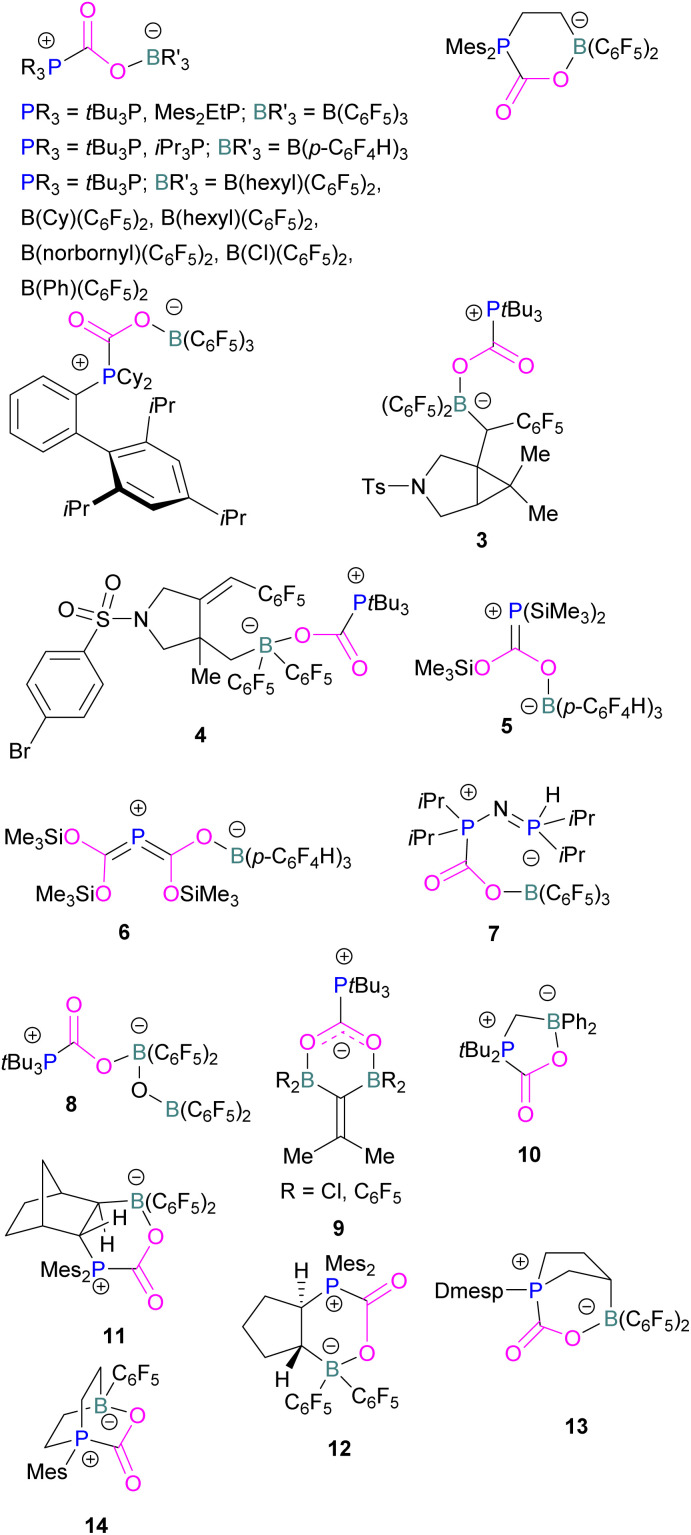
Examples of FLP adducts of boranes with a phosphorus Lewis base. Dmesp = Dimesitylphenyl.

At the time, these systems offered rare examples of metal-free CO_2_ sequestration.^34^ Theoretical investigations show the mechanism proceeding by simultaneous formation of P–C and O–B bonds from thermochemical computed data (B97-D/TZVPP’, B2PLYP-D/TZVPP’, and B2PLYP-D/QZVP(-g, -f) levels of theory). *t*Bu_3_P reacts with B(C_6_F_5_)_3_ at room temperature and under 1 bar of CO_2_ forms the desired stable product *t*Bu_3_P-CO_2_-B(C_6_F_5_)_3_, which upon heating at 80 °C under vacuum releases the CO_2_ molecule and regenerates the starting FLP mixture. Calculations for the formation of *t*Bu_3_P-CO_2_-B(C_6_F_5_)_3_ show that the overall reaction is exothermic. Privalov and co-workers calculated several energy pathways for CO_2_ activation.^35^ After these first reports of non-metal based inter- and intramolecular FLP-mediated reversible CO_2_ activation, the scientific community explored a range of new FLPs for CO_2_ activation, as shown in Fig. 4. Several other phosphine bases and boron acids have been used in the intermolecular system including *i*Pr_3_P, XPhos and Mes_2_EtP, and B(*p*-C_6_F_4_H)_3_, and B(R)(C_6_F_4_H)_2_, (R = hexyl, Cl, cyclohexyl, norbornyl, Ph).^36,37^ More complex boranes bearing functionalised substituents with cyclic structures were also tested with *t*Bu_3_P to trap CO_2_ generating adducts 3 and 4.^38^

**Scheme 6:**
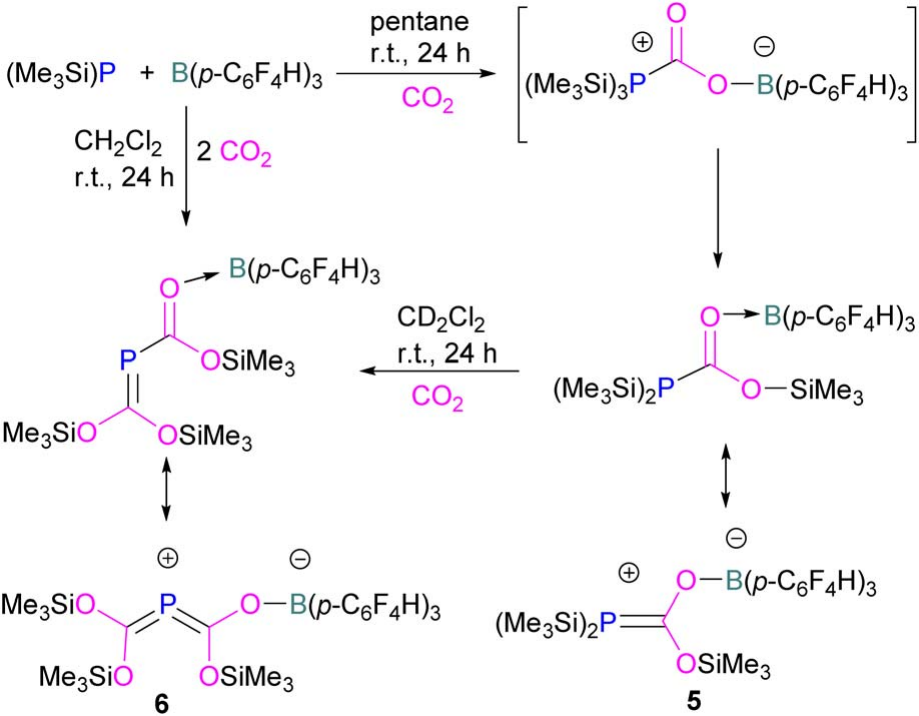
Silyl migration in the (Me_3_Si)_3_P and B(*p*-C_6_F_4_H)_3_ FLP.

**Scheme 7:**
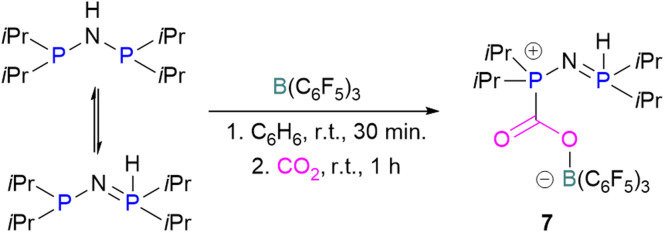
H-isomerisation with a migration of the proton from nitrogen to phosphorus.

Interestingly, when (Me_3_Si)_3_P was utilised as a Lewis base instead of *t*Bu_3_P, with B(*p*-C_6_F_4_H)_3_, silyl migration was observed in the final adducts 5 and 6 (Fig. 4 and Scheme 6). *t*Bu_3_P and (Me_3_Si)_3_P with CO_2_ in pentane at room temperature initially yields the expected adduct (Me_3_Si)_3_P-CO_2_-B(*p*-C_6_F_4_H)_3_. Subsequently, silyl migration from phosphorus to oxygen forms a stable compound which can be better represented as the zwitterionic compound 5. The same starting Lewis acid and base can also react with two equivalents of CO_2_ in dichloromethane (CH_2_Cl_2_) at room temperature for 24 h, providing silyl migrated product 6. Compound 6 can also be obtained from 5, when 5 is treated with CO_2_ in CD_2_Cl_2_ at room temperature for 24 h (Scheme 6).^39^ Kemp and co-workers on the other hand, investigated the phosphine base bis(di-i-propylphosphino)amine with B(C_6_F_5_)_3_ which formed the expected 1 : 1 adduct 7. The crystal structure of 7 shows that H-isomerisation took place with a migration of the proton from nitrogen to phosphorus (Scheme 7).^40^

Stephan and co-workers have expanded the borane scope to explore the reactivity of bis-boranes to trap CO_2_ with *t*Bu_3_P. It was also found that, 1,1-bis-(C_6_F_5_)_2_BOB(C_6_F_5_)_2_ binds with CO_2_ in a monodentate manner generating 8, whilst bis-boranes of type (R_2_B)_2_C=CMe_2_, where R = Cl or C_6_F_5,_ provide a bidentate chelation of CO_2_ to obtain a unique type of heterocyclic compounds 9.^41^ The chelation of CO_2_ by the two B-centres in 8 was restrained due to steric crowding as well as a significant π-character in the B–O bonds, which was evident from the relatively large B–O–B bond angle of 139.5(2)°. Whereas, in 9 B–C–B angles of 117.3(2)° (R = Cl) and 121.2(2)° (R = C_6_F_5_) show a six membered planar structure.

**Fig. 5:**
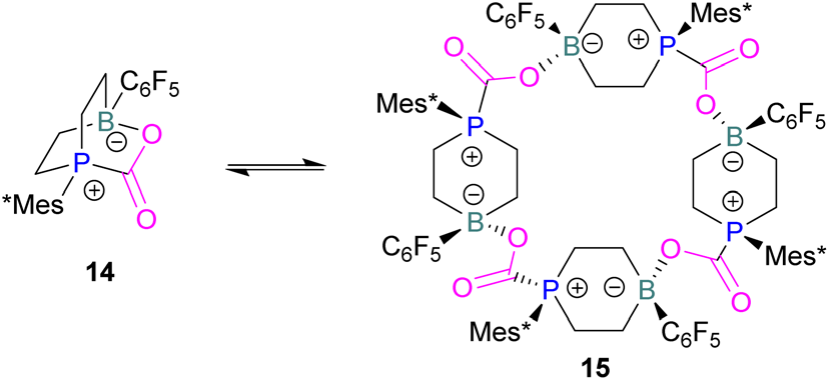
FLP adducts of boranes with P-base. Mes* = 2,4,6-Tri-*tert*-butylphenyl.

In intramolecular systems, when the Lewis base and acid are sufficiently aligned in a geminal fashion, an increase in reactivity is observed as seen in the formation of adduct 10.^42^ Another intramolecular FLP with a norbornane structure with a vicinal designed FLP was utilised to trap CO_2_ to obtain adduct 11.^43^ Similarly to the vicinal FLP in adduct 11, an FLP based on cyclopentane with *trans*-1,2-substituents was explored to form 12.^44^ Other intramolecular B/P FLP systems include an active FLP borylated tetrahydrophosphole which yielded adduct 13,^45^ and a cyclic six membered FLP with 1,4-phosphane/borane substituents which undergoes an addition reaction with CO_2_ to form adduct 14. Erker and co-workers observed that heating 14 in *n*-heptane at 80 °C for 15 min under CO_2_ converts 14 to its cyclotetrameric macrocyclic oligomer 15. Tetramer 15 is unstable in solution, even in a CO_2_ atmosphere it slowly converts back to the monomer 14 (Fig. 5).^46^

**Scheme 8:**
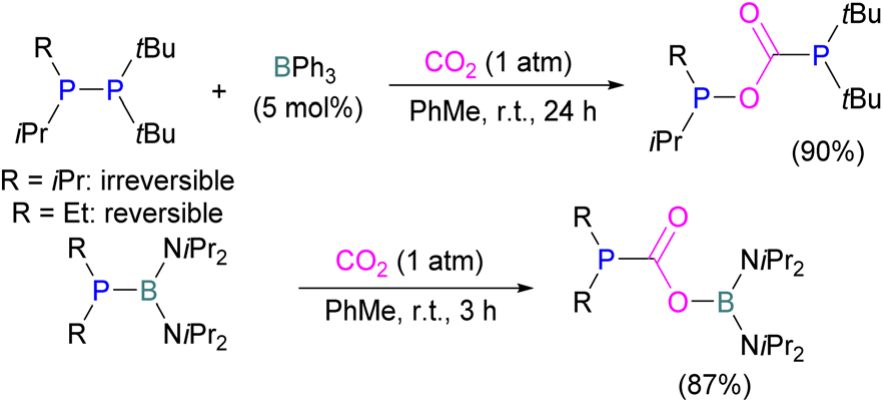
Diphosphination (top) and phosphinoboration (bottom) of CO_2_.

**Scheme 9:**
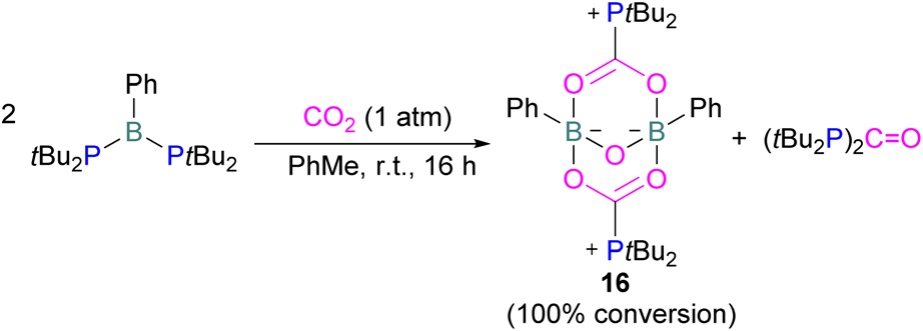
Synthesis of phenyl oxoborane.

Szynkiewicz and co-workers reported the phosphinoboration and diphosphination of CO_2_. In 2019, they published the first report of catalytic (with respect to the borane) diphosphination of CO_2_ with a diphosphane/boron FLP. CO_2_ was inserted into the relatively weak P–P bond (Scheme 8).^47^ Furthermore in 2019, they reported the use of diaminophosphinoboranes to phosphinoborate CO_2_; though not sterically frustrated, this compound still exhibits FLP-like reactivity (Scheme 8, bottom).^48^ More recently the same group built on this work, reporting the reaction of CO_2_ (among several other small molecules) with a diphosphinoborane B(P*t*Bu_2_)_2_Ph to yield a diphospha-urea and a bicyclic diboroxane 16 (Scheme 9). The reaction proceeds by CO_2_ insertion into a single B–P bond, elimination of (*t*Bu_2_P)_2_C=O to give phenyl oxoborane PhBO. Reaction of this species with a further equivalent of the parent diphosphinoborane and 2 equivalents of CO_2_ gives the product 16. While stable under N_2_, the product decomposes with loss of CO_2_ and diphospha-urea to give triphenylboroxine (PhBO)_3_.^49^

**Scheme 10:**
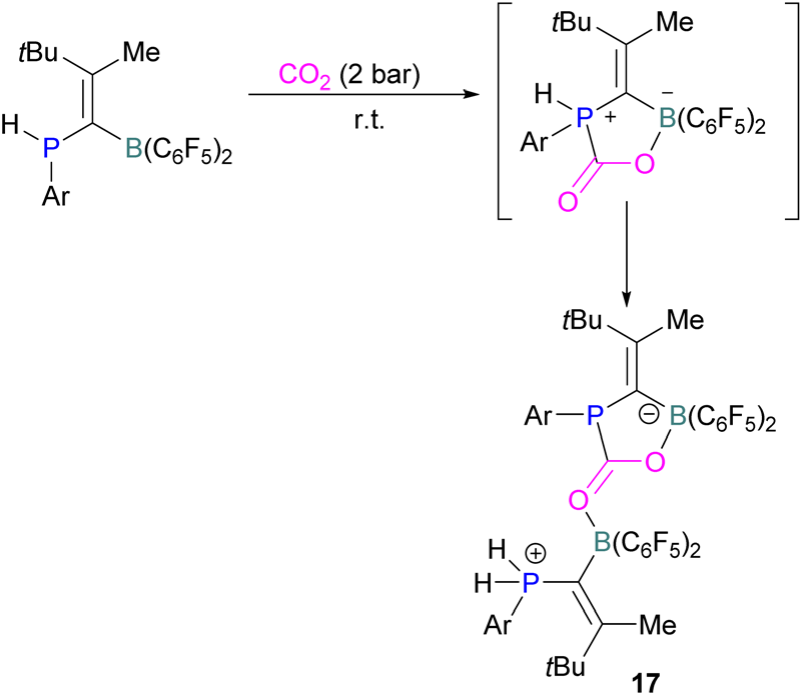
Reaction of geminal vinylidene-bridged P/B Lewis pair with CO_2_.

Kessete and co-workers published a computational study evaluating a number of intramolecular phosphine/borane catalysts for CO_2_ reduction. One finding was that, though electron-withdrawing substituents on borane like fluorine stabilise the CO_2_-FLP adduct, they also destabilise the transition state (TS), increasing activation energy. Fluorination of the substituents of the phosphorus reduces its basicity and so destabilises both the transition state and the adduct formed. This highlights the importance of tuning the Lewis acidic and basic sites of FLPs to achieve stabilised transition states, but that are also Lewis acidic, or Lewis basic enough centres to bind with CO_2._^50^ Jian and co-workers reported in 2017 that geminal vinylidene-bridged phosphorus/boron Lewis pairs could react with CO_2_ to give a phosphinodiborated product 17, as shown in Scheme 10. Interestingly, this geminal P/B is supported with an sp^2^ carbon, which is different from previous reports of geminal FLPs.^51^

**Scheme 11:**
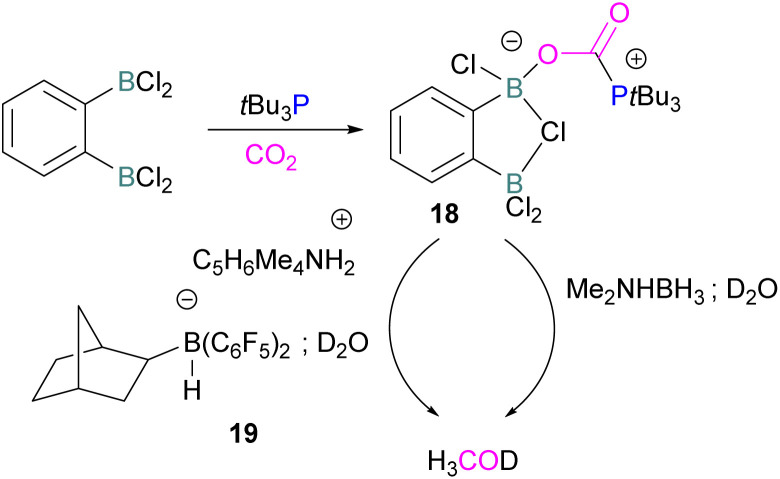
Stoichiometric reduction of FLP-CO_2_ adduct 18.

Following activation of CO_2_, the subsequent transformations have been investigated initially stoichiometrically. Stephan and co-workers reported that the bis-borane, 1,2-C_6_H_4_(BCl_2_)_2_, forms an adduct with *t*Bu_3_P and also shows FLP reactivity with CO_2_ to form the FLP-CO_2_ zwitterionic compound 18 (Scheme 11). Compound 18 is remarkably more stable, with respect to the loss of CO_2_, and no decomposition was observed even on heating to 80 °C for 24 h compared to the CO_2_ adducts obtained from FLPs *t*Bu_3_P/B(C_6_F_5_)_3_ (loss of CO_2_ at 80 °C), Mes_2_PCH_2_CH_2_B(C_6_F_5_)_2_ (loss of CO_2_ at −20 °C), and bis-boranes Me_2_C=C(BR_2_)_2_ where R=Cl, C_6_F_5_ with *t*Bu_3_P (loss of CO_2_ at 15 °C). The chlorine atom in 18 bridges between the boron centres which enhances the Lewis acidity of the boron bound with the oxygen atom of CO_2_ and results in a stronger B–O bond making the adduct more thermally stable, than other discussed examples. Hence, the strength of the bond between the Lewis acid and the oxygen atom of CO_2_ plays a critical role in establishing reversibility, this can be induced by the addition of electron-withdrawing groups, such as Cl in 18. The species 18 was reduced by Me_2_NHBH_3_ followed by quenching with deuterated water (D_2_O) to obtain deuterated methanol (MeOD) as the final product. In another way, 18 was also reduced by [C_5_H_6_Me_4_NH_2_]/[HB(C_6_F_5_)_2_(C_7_H_11_)] (19) and quenched with D_2_O again yielding H_3_COD (Scheme 11). Here, two Lewis acidic boron sites are available, and bridging of a chlorine atom between the two stabilises the zwitterionic adduct.^52^

There remain two major issues with *t*Bu_3_P/1,2-C_6_H_4_(BCl_2_)_2_ that pose limitations for a catalytic cycle. The first issue is that H_2_ cannot be activated, so H_2_ surrogates such as Me_2_NHBH_3_ or [C_5_H_6_Me_4_NH_2_]/[HB(C_6_F_5_)_2_(C_7_H_11_)] were utilised as stoichiometric reductants. Secondly, the boron centre in this FLP is more oxophilic, so the last step required quenching with D_2_O to cleave the B–O bond.

**Scheme 12:**
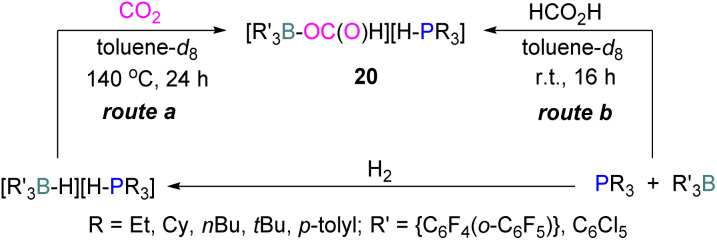
A series of FLPs consisting of C_6_F_4_(*o*-C_6_F_5_)}_3_B or (C_6_Cl_5_)_3_B with trialkylphosphines, and their reduction reactions.

In another stoichiometric system, O'Hare and co-workers synthesised a series of FLPs based on Lewis acid {C_6_F_4_(*o*-C_6_F_5_)}_3_B and (C_6_Cl_5_)_3_B with trialkylphosphines as Lewis bases (Scheme 12). The idea of synthesising these FLPs was to achieve a weaker B–O bond to facilitate the cleavage of B–O bond upon reduction and potentially generate a catalytic system. The steric congestion factor was applied as steric bulk at the *ortho* position alone could decrease the B–O bond strength.^53^ The synthesised FLPs were exposed to H_2_ to form FLP-H_2_ as activated salts of the type [R_3_P–H][H–BR′_3_]. These salts were then exposed to CO_2_ (1 atm) to obtain formatoborates of type 20 in the presence of toluene at 140 °C for 24 h using Young's tap NMR tubes. The formatoborates 20 could also be prepared independently from the reaction of the FLP with formic acid in toluene at room temperature for 16 h (Scheme 12).^54^ The formatoborates 20 were subjected to H_2_ and heated to 140 °C for 16 h but were not reduced, instead decarboxylation of the formatoborates 20 occurred and hydride salts were formed with no further reductions.

The higher stability of the formatoborates 20 and their decarboxylation at higher temperatures limited this FLP approach for a catalytic reduction of CO_2_.

**Scheme 13:**
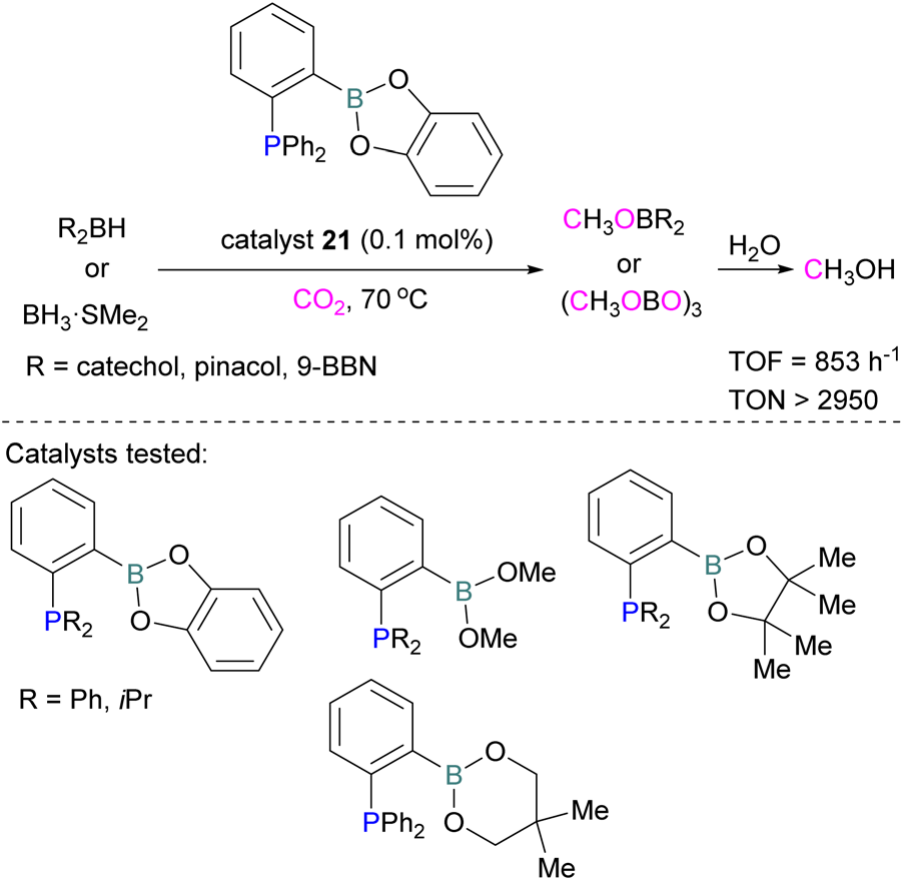
Catalytic performance of 21 in reduction of CO_2_ for methanol synthesis (top), and different FLP catalysts tested (bottom).

**Scheme 14:**
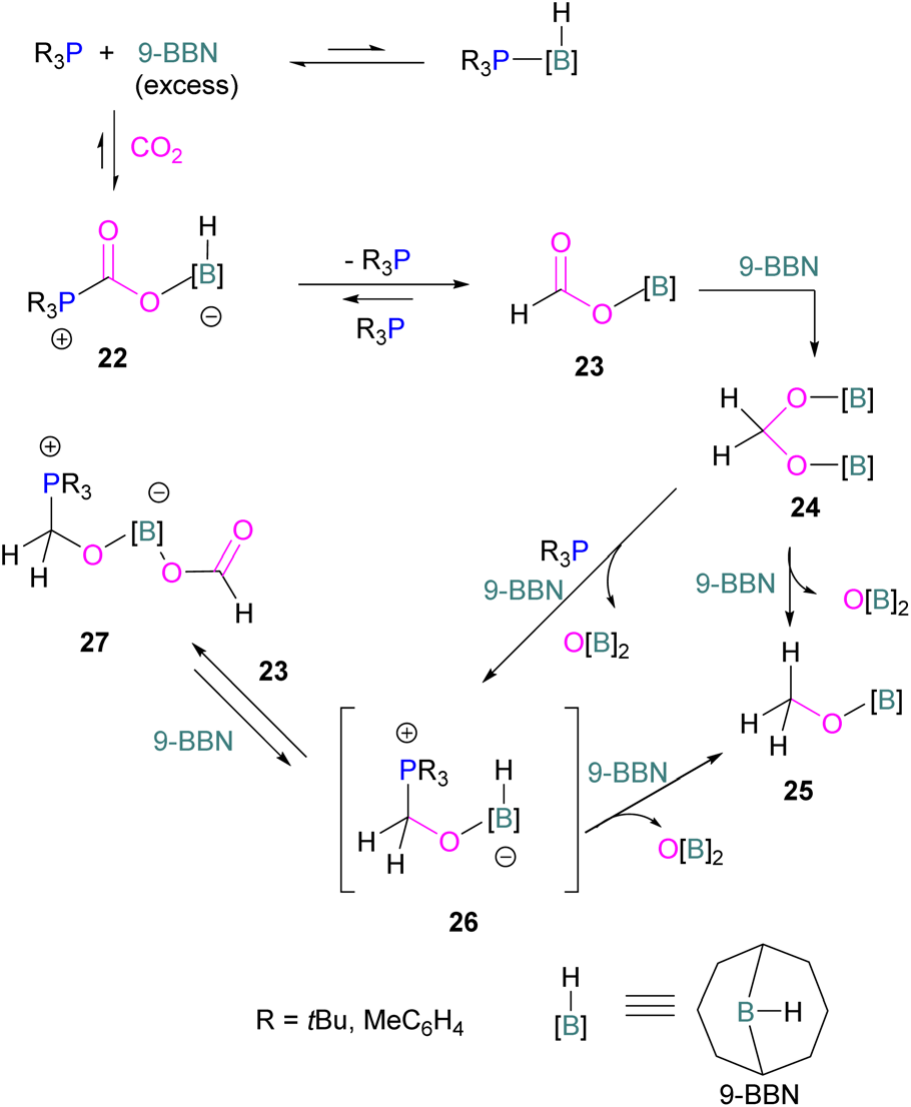
Phosphine catalysed CO_2_ reduction with 9-BBN.

Following reports on stoichiometric reactions, the first catalytic reduction of CO_2_ with an organocatalyst FLP was explored by Fontaine and co-workers in 2013. They applied hydroboranes HBR_2_ [HBcat (catecholborane), HBpin (pinacolborane), 9-BBN (9-borabicyclo[3.3.1]nonane), BH_3_·SMe_2_ and BH_3_·THF] to produce CH_3_OBR_2_ or (CH_3_OBO)_3_ following reduction of CO_2_. Upon hydrolysis, CH_3_OBR_2_ or (CH_3_OBO)_3_ yield methanol as the final product in up to 99% yield (Scheme 13) with high turnover numbers (TON > 2950) and turnover frequencies (TOF = 853 h^−1^). The intramolecular phosphino-borane catalyst 21 was found to be an efficient catalyst for this reaction.^55^ The same authors studied the mechanism of this hydroboration of CO_2_ with catalyst 21 using computational and experimental methods. It was found that an intramolecular FLP was involved in every step of the reduction and the simultaneous activation of both, the reducing agent and CO_2_, were the key to efficient catalysis in every reduction step.^56^ Furthermore, Fontaine and co-workers synthesised various phosphine–borane derivatives of catalyst 21 with different substituents on boron and phosphorus as shown in Scheme 13 (bottom). These were then tested for hydroboration of CO_2_ using HBcat or BH_3_·SMe_2_ to generate methoxyboranes. The most active species were derivatives with a catechol unit on boron. They also performed isotope labelling experiments and DFT studies and found that once the formaldehyde adduct was generated, the CH_2_O moiety remained on the catalyst system. The lowest energy barriers were found for concerted activation of catecholborane by the Lewis base and of CO_2_ by the Lewis acid. The results show higher potency of “O” for the activation of hydroboranes than “P”.^57^ Overall, FLP 21 acted as an efficient catalyst because of two important features: Firstly, 21 did not form an adduct with CO_2_, as seen previously with most FLPs that formed a stable CO_2_ adduct. Exposing 21 to 1 atm of CO_2_ at room temperature resulted in no spectroscopic change of the solution (by ^1^H, ^31^P, and ^11^B NMR spectroscopy). Also, species 21 remained monomeric in solution without any P–B interaction. Secondly, the CH_2_O moiety was released upon reduction from the catalyst and made 21 available for another reaction. Hence, the higher high turnover numbers and high turnover frequencies for 21. Later, Stephan and co-workers developed another catalytic method for the reduction of CO_2_ using 9-BBN as a reducing agent and phosphine as a catalyst (Scheme 14). The reaction proceeds *via* an FLP-type CO_2_ activation intermediate 22 and the reduction products include boron-bound formate species, 23, the diolate-linked compound 24, and methoxide product 25. Intermediate 26 could be transferred to 25 in the presence of 9-BBN and to 27 in the presence of the boron-bound formate species 23. Derivatives of 27 were isolated and confirmed with single crystal X-ray diffraction analysis. With 0.02 mol% of *t*Bu_3_P, product 25 is obtained in 98% yield at reaction temperature 60 °C. In the best scenario, the catalyst *t*Bu_3_P provides 5556 turnovers of hydride transfers to CO_2_ and a TOF of 176 h^−1^.^58^

Instead of forming a classical adduct of *t*Bu_3_P and 9-BBN, this system showed an FLP-type CO_2_ activation and subsequent hydride transfer from boron to the carbonyl carbon in 22, releasing *t*Bu_3_P for the next cycle and hence this system worked catalytically for the reduction of CO_2_.

**Scheme 15:**
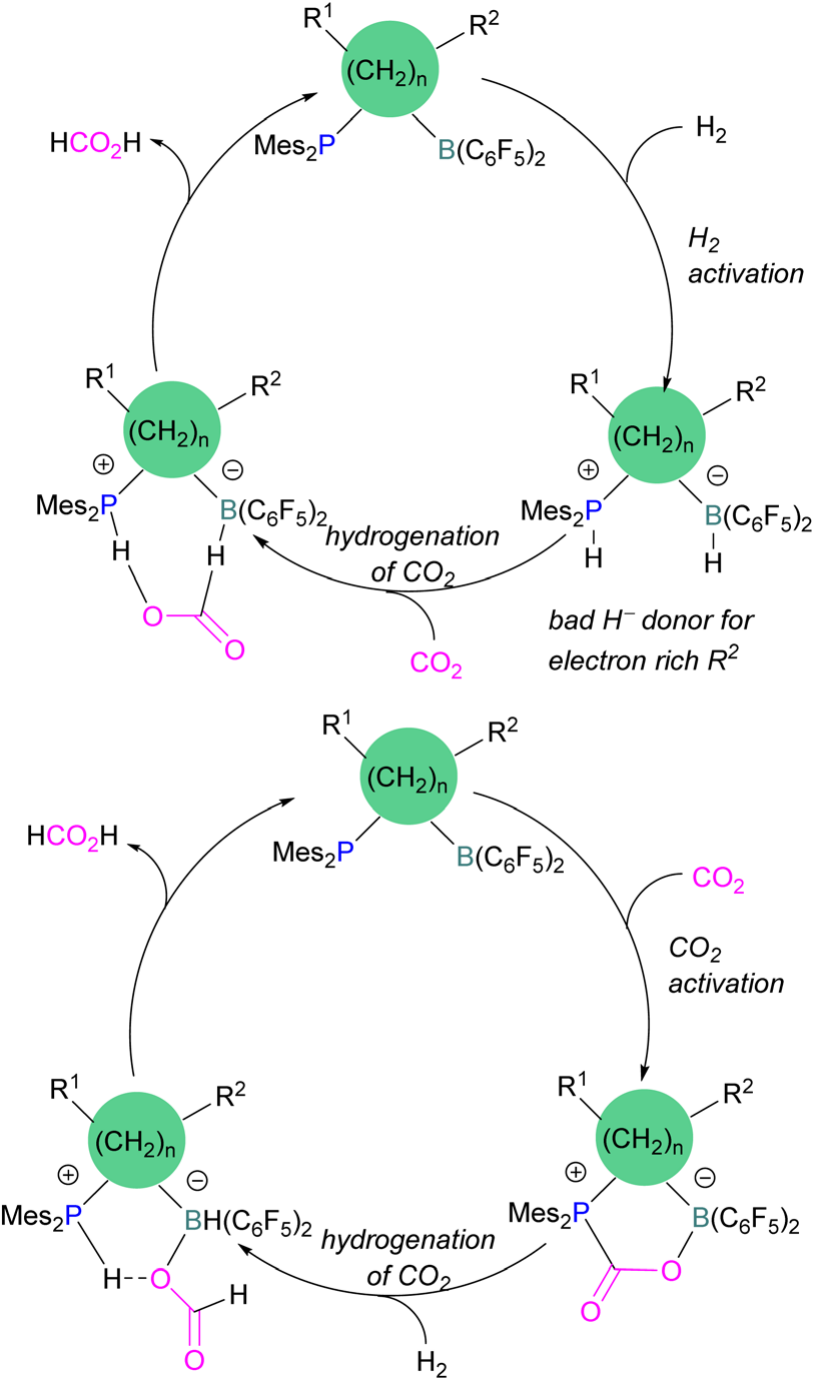
Two different calculated reduction cycles for CO_2_ in bridged FLPs.

Dang and co-workers reported a theoretical study on a catalytic mechanism for computationally designed bridged P/B FLPs in the activation of H_2_ and CO_2_. They found that the reaction follows a one-step concerted mechanism with small reaction barriers (14.8–24.0 kcal mol^−1^). Among the computationally designed bridged FLPs, some were found to successfully reduce CO_2_ with molecular hydrogen in two feasible pathways. The first pathway follows immediate hydrogenation of CO_2_ after H_2_ activation (Scheme 15, top), the second follows CO_2_ activation first, then metathesis of H_2_ followed by reductive elimination (Scheme 15, bottom). Both catalytic cycles provide the product HCO_2_H from the reduction of CO_2_. From all computationally designed bridged FLPs, straightforward H_2_ activation takes place with those that do not have electron donating substitutions on the B's adjacent carbon site, or have a long chain between the B and P.^59^

**Scheme 16:**
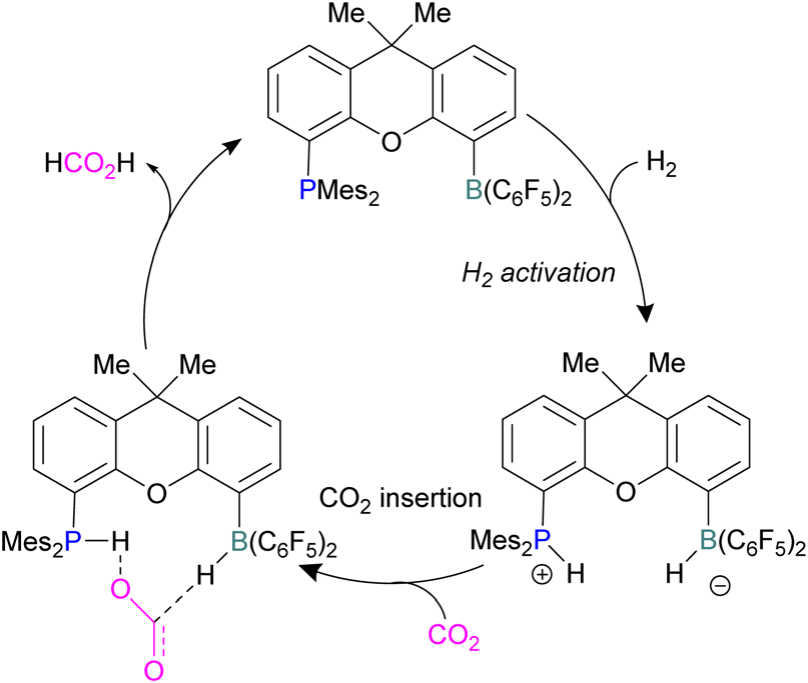
Xanthene FLPs mediated catalytic reduction of CO_2_ into formic acid.

Xanthene FLPs were computationally investigated using DFT methods [level of theory: B3LYP-D3/6-311+G*(*)//M06-2X/6-31G*(*) in bromobenzene], and their reduction of CO_2_ was modelled as shown in Scheme 16. Differently substituted xanthene backbones were investigated, showing that more rigid backbones have lower activation energies for CO_2_ hydrogenation.

The formation of P/B–H in the first step was shown to be exergonic and this first intermediate is the catalyst resting stage.^60^ The hydride transfer from boron to the carbonyl carbon of CO_2_ produces formate and subsequent protonation resulted in the formation of formic acid bringing the xanthene FLP into the next cycle for the CO_2_ reduction.

**Scheme 17:**
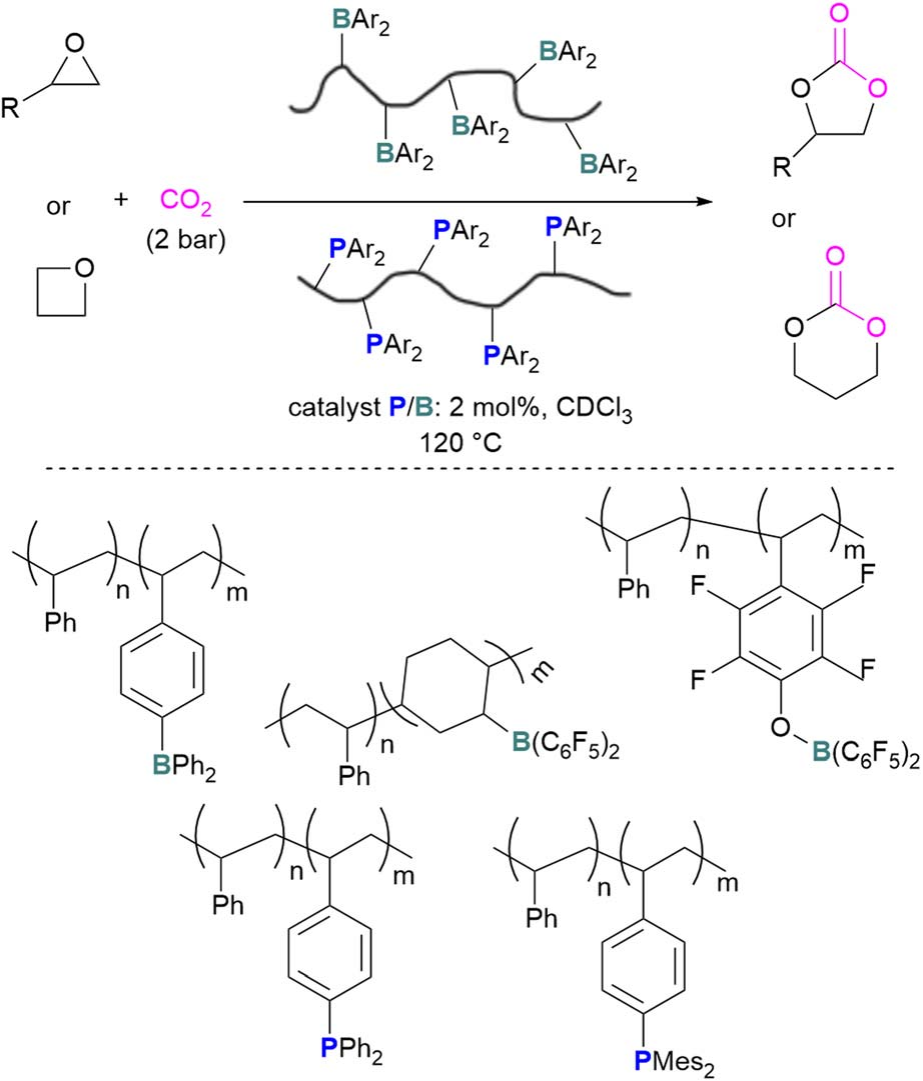
Polymeric FLP-catalysed reaction of ethers and CO_2_ for cyclic carbonate formation (top), and polymers used (bottom).

The incorporation of FLPs into polymers has also seen some success in CO_2_ activation. Shaver and co-workers explored the first use of polymeric FLPs to catalyse the incorporation of CO_2_ into cyclic ethers for the formation of cyclic carbonates and showed good selectivity (Scheme 17, top). Different phopshines and boranes were explored as the Lewis base and acid in the polymer (Scheme 17, bottom). These poly(FLPs) can easily be recovered and reused after the reaction, however the efficiency of the catalyst gradually decreases due to partial phosphine oxidation and increased crosslinking.^61^

**Scheme 18:**
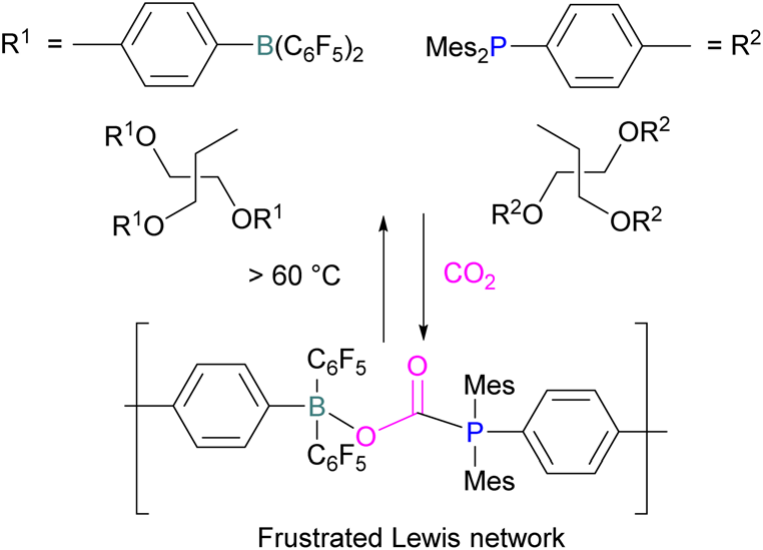
CO_2_-responsive dynamic gel system based on a FLPs.

Yan and co-workers developed CO_2_-responsive dynamic gel system based on an FLP for the first time (Scheme 18). Here, CO_2_ can be regarded as a “gas glue” which crosslinks the Lewis acidic and Lewis basic sites and forms a new type of a FLP network. The trapped CO_2_ FLP network undergoes reversible release of CO_2_ upon heating at >60 °C.

**Scheme 19:**
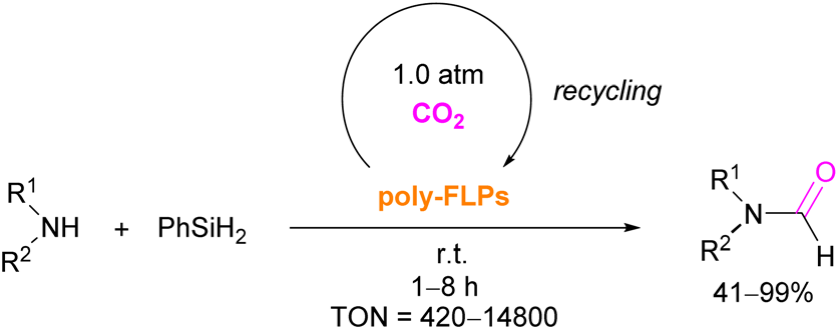
–B–CO_2_–P– poly-FLPs in formamide synthesis.

The authors found that CO_2_-bridging crosslinks in the network are dynamic covalent linkages, which provides the gel with unique gas-tuneable viscoelastic, mechanical, and self-healing characteristics.^62^ The authors showed that the same (–B–CO_2_–P–) poly-FLPs are efficient catalysts in transforming amine substrates to formamide derivatives using the CO_2_ poly-FLP as the starting point. Various amines were screened and the yields for the formamide products were in the range of 41–99% with TON = 420–14 800. The highest TON of 14 800 was observed for diethylamine giving 99% yield of the corresponding diethylformamide product (Scheme 19).^63^ CO_2_ bridges the polymer chains and a CO_2_ -triggered micellisation was obtained. Addition of PhSiH_3_ and R^1^R^2^NH resulted in the desired formamide products and regenerated the polymer. After separation of the products re-micellisation of the polymers was performed with CO_2_ and a reusable catalytic system was established with a high turnover number.

**Scheme 20:**
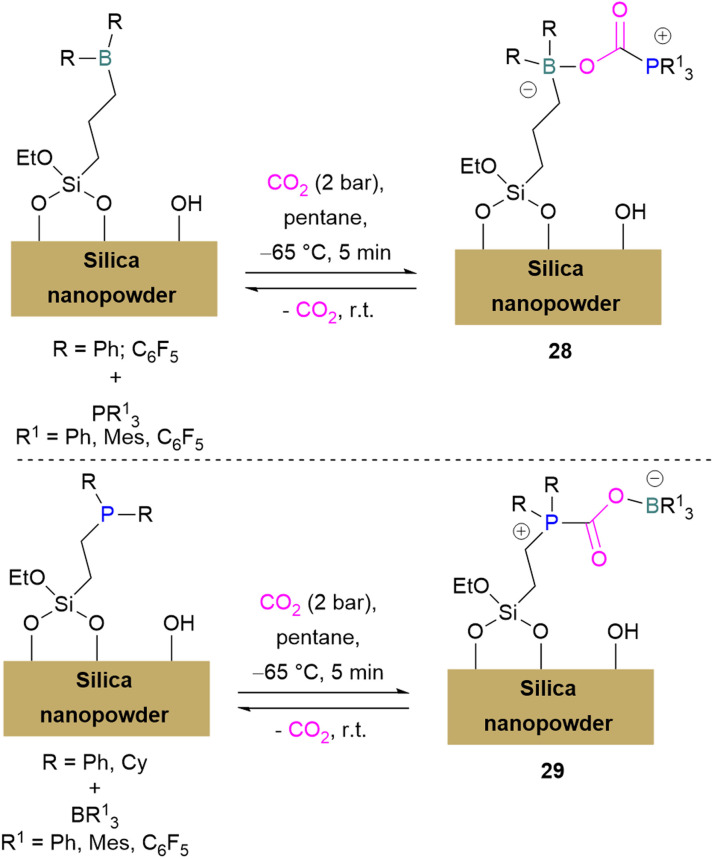
FLPs supported on silica nano-powder for CO_2_ capture.

Erasmus and co-workers developed efficient FLPs supported on silica nano-powder for CO_2_ capture.^64^ A series of CO_2_ adducts 28 were synthesised by reacting silica nanopowder supported Lewis acids and dissolved Lewis bases in pentane with CO_2_ (2 bar) which was passed through the pentane mixture at −65 °C. At room temperature these adducts were observed to be reversible in nature (Scheme 20, top).

In a similar manner a series of silica nano-powder supported FLP-CO_2_ adducts 29 were synthesised from silica nano-powder supported Lewis bases and dissolved Lewis acids. The silica nanopowder supported FLPs were also explored for the conversion of CO_2_ to formic acid using hydrogen gas. Initially, the activation of H_2_ was done by the supported Lewis acid/bases with FLP partners to obtain [–BH]^−^ [HP–]^+^ salts. Furthermore, introducing CO_2_ to these salts resulted in HCO_2_H and regenerated the FLPs. HCO_2_H is a protic polar molecule and has tendency to form O⋯H bonds with the free –OH functionalities on the silica. The main reason for the release of HCO_2_H from the system after reduction was the immobility of silica nanopowder bound Lewis acids (or Lewis bases) and so did not inhibit the activity of the FLPs.

In FLP systems having a P-basic centre, activation of CO_2_ proceeds *via* the formation of a P–C bond, and depending on the type of reactive acidic site a B–O, Al–O or Ga–O bonds are generated, often in a reversible manner. Alkyl phosphines *i*Pr_3_P or *t*Bu_3_P alone could not activate the CO_2_ molecule. It is known that the presence of a Lewis acidic component is not necessary for capturing CO_2_ when very electron rich *P*-nucleophiles are used.^65^

### Borane/nitrogen FLPs for CO_2_ activation

**Fig. 6:**
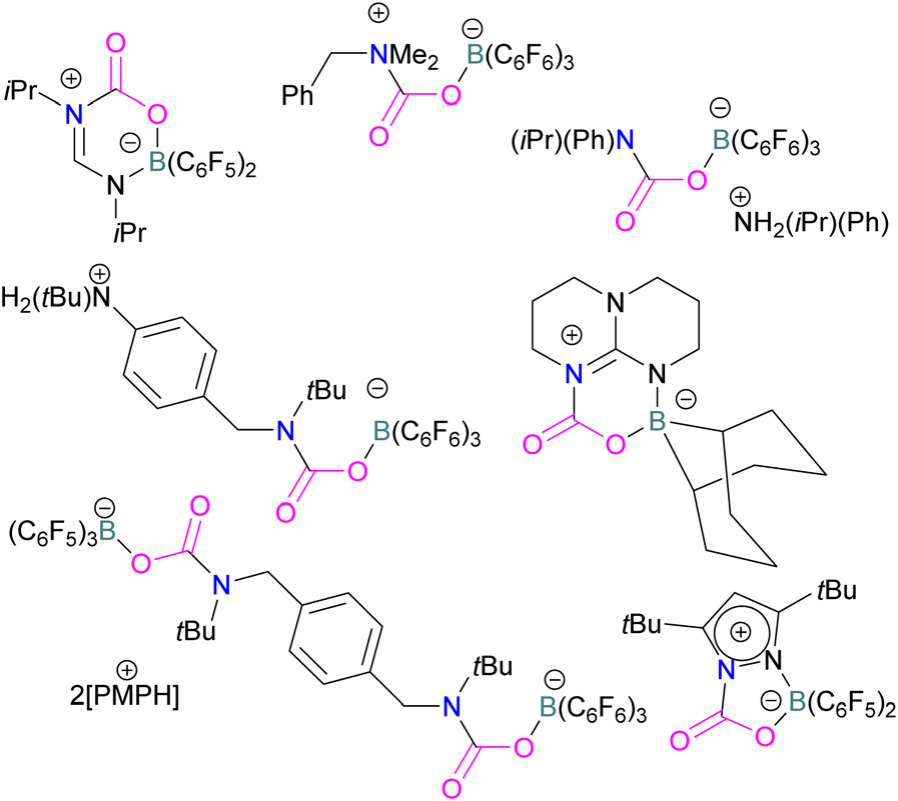
Structures of N–CO_2_–B adducts. PMP = 1,2,2,6,6-Pentamethylpiperidine.

In addition to phosphorus as a Lewis base in FLP-CO_2_ activation and reduction, there has been a wealth of FLPs described in the literature that use a nitrogen Lewis base in combination with a boron Lewis acid. A selection of the corresponding FLP-CO_2_ adducts are displayed in Fig. 6.^66,67^

**Scheme 21:**
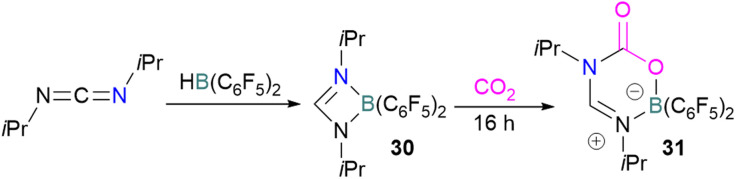
Synthesis of boron amidinates and reaction with CO_2_.

Stephan and co-workers reported a new synthetic method for making boron amidinates. The strained ring boron amidinate derivative 30 was prepared by reacting Piers' borane, HB(C_6_F_5_)_2_, with isopropyl carbodiimide. 30 was then successfully employed to trap CO_2_ incorporated into a new heterocycle 31 (Scheme 21). Compound 31 was fully characterised along with a single crystal X-ray diffraction structure.^68^ A theoretical study on the reaction of 30 with CO_2_ found a concerted addition mechanism.

**Scheme 22:**
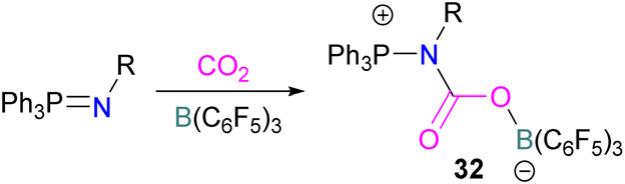
Activation of CO_2_ using phosphinimines and B(C_6_F_5_)_3_.

**Scheme 23:**
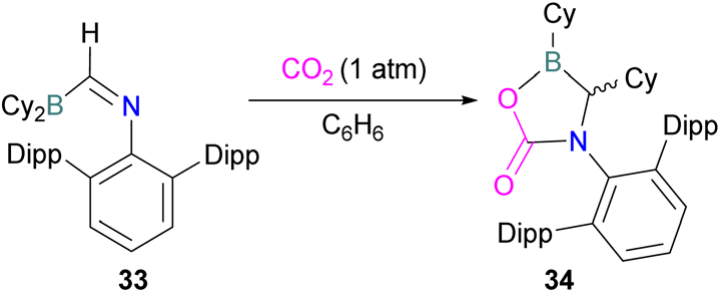
CO_2_ adduct of (boryl)iminomethane. Dipp = 2,6-Diisopropylphenyl.

In this reaction, the C-atom and O-atom of CO_2_ inserts into the B–N bond of 30 and forms the C–N and B–O bonds simultaneously. The frontier orbitals involved in the reaction mechanism were investigated as well as electric charge analysis and showed that results were consistent with charge transfer from HOMO of 30 to the LUMO of CO_2_.^69^ In another findings, Chattaraj and co-workers have studied this boron amidinate 30 as a bridged B/N FLP.^70^ They compared 30 with a P/B bridged system shown in Scheme 15 which describes two types of cycles. In this work, a similar process shows that CO_2_ hydrogenation with amidinate 30 leads to formic acid (HCO_2_H) as the final product. In the proposed mechanisms, either H_2_ is activated by the Lewis basic centre of the FLP, and CO_2_ is activated by the Lewis acidic centre of the FLP, or alternatively, CO_2_ can be activated by Lewis basic centre of the FLP and H_2_ by Lewis acidic centre of the FLP. In both cases, simultaneous activation of CO_2_ and H_2_ by a single TS was confirmed by Natural Bond Orbital (NBO) analysis and this TS is the rate determining step. From energy decomposition analysis (EDA), in the TS geometry it was found that electron density was donated from the HOMO of FLP to the LUMO of H_2_ and electron density from HOMO of H_2_ molecule to the LUMO of CO_2_.^70^ Stephan and co-workers have also utilised phosphinimines and B(C_6_F_5_)_3_ to explore FLP reactivity, Ph_3_P=NR with B(C_6_F_5_)_3_ and CO_2_ produced the adducts 32 (R = Ph, C_6_F_5_) (Scheme 22).^71^ Figueroa and co-workers observed that (boryl)iminomethane 33 reacts intramolecularly with CO_2_ and forms a five-membered ring 34 in a 1,2-cyclohexyl shift (Scheme 23). Due to the 1,2-cyclohexyl shift, product 34 is stable and prevents the release of CO_2_, exhibiting irreversibility. Heating of the solution of 34 to 80 °C showed no release of CO_2_. Likewise, heating of the solid sample of 34 to 150 °C under vacuum did not display any CO_2_ release either.^72^

**Scheme 24:**
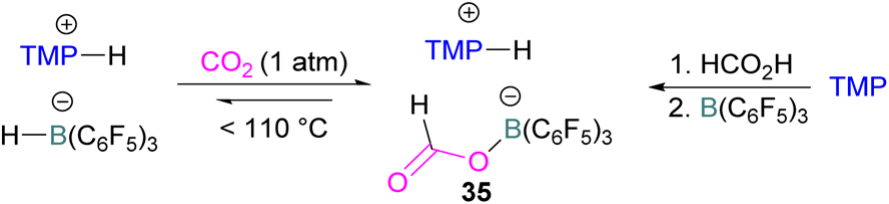
Formation of a formatoborate complex with the TMP/B(C_6_F_5_)_3_ FLP.

**Scheme 25:**
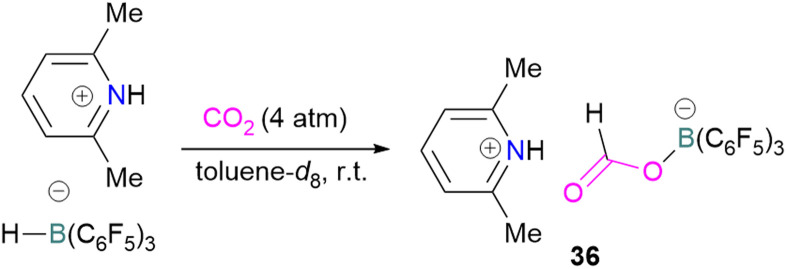
Formation of a formatoborate complex with the 2,6-lutidine/B(C_6_F_5_)_3_ FLP.

**Scheme 26:**
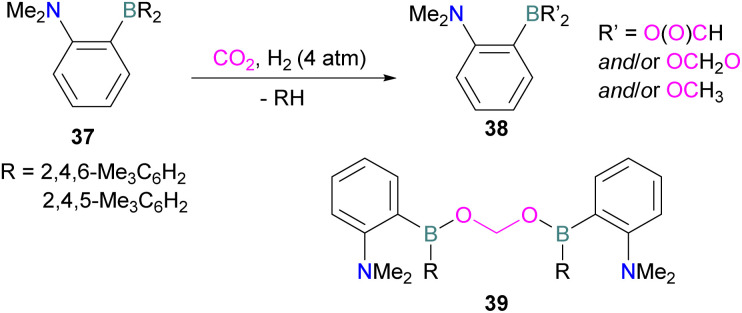
Hydrogenation of CO_2_ using intramolecular B/N FLPs.

N/B CO_2_ adducts have also been used in subsequent stoichiometric transformations. It has been previously reported that TMP [2,2,6,6-(tetramethylpiperidine)] along with B(C_6_F_5_)_3_ splits H_2_ heterolytically and forms the ion pair [TMPH][HB(C_6_F_5_)_3_].^73^ O'Hare and co-workers utilised this ion pair [TMPH][HB(C_6_F_5_)_3_] to insert CO_2_ into the B–H bond forming a formatoborate complex 35 at elevated temperatures. Compound 35 can also be obtained from the reaction of TMP, B(C_6_F_5_)_3_ and HCO_2_H (Scheme 24). The structure of 35 was confirmed by single crystal X-ray diffraction analysis.^74^ The formatoborate complex 35 could be transformed to produce MeOH by applying more equivalent of ion pair [TMPH][HB(C_6_F_5_)_3_]. The formation of [(C_6_F_5_)_3_B–OH]^−^ is an obstacle for this method to be developed into a catalytic transformation. A similar FLP system consisting of 2,6-lutidine/B(C_6_F_5_)_3_ has also been shown to split H_2_ heterolytically to form borohydride salt [(CH_3_)_2_C_5_H_3_NH][HB(C_6_F_5_)_3_].^75^ Mayer and co-workers applied this salt for the activation of CO_2_ at 4 atm pressure and at room temperature. The air-stable formatoborate complex 36 (Scheme 25) resulted and its structure was confirmed by X-ray diffraction analysis.^76^ Compared to 35, Mayer and co-workers observed that 36 on heating to 80 °C resulted only in decomposition instead of transforming to other CO_2_ reduced products. This restricts the method to obtain only formatoborate complex 36 in a stoichiometric way. Fontaine and co-workers explored the hydrogenation of carbon dioxide using intramolecular *o*-phenylene bridged B/N FLPs 37 (Scheme 26). When R = 2,4,6-Me_3_C_6_H_2_, the FLP species forms the formyl, acetal and methoxy derivatives 38, but when R = 2,4,5-Me_3_C_6_H_2_, the boron-linked product 39 formed instead.^77^

**Scheme 27:**
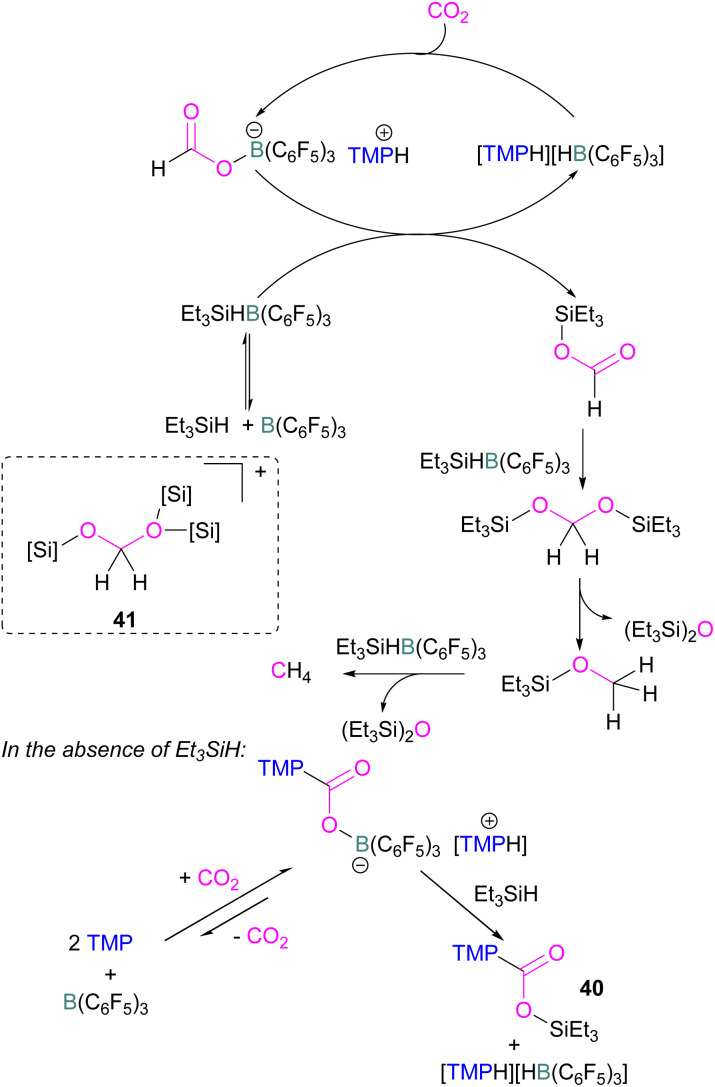
Converting stoichiometric CO_2_ reduction into a catalytic process by adding excess B(C_6_F_5_)_3_ and Et_3_SiH to the TMP/B(C_6_F_5_)_3_ FLP system.

Catalytic transformations of CO_2_ have also been successful using B/N FLP systems. To address the catalytic shortcomings of the reaction developed by O'Hare and his group using the FLP TMP/B(C_6_F_5_)_3_ for the reduction of CO_2_ with H_2_ to form methanol, Piers and co-workers developed a catalytic method by adding silane to the reaction mixture with excess B(C_6_F_5_)_3_ to form methane (Scheme 27).^78^ They also reported that when Et_3_SiH was not added to the reaction, then the CO_2_ adduct as the salt [TMP-CO_2_-B(C_6_F_5_)_3_][TMPH] was formed. As seen in other systems, the formation of the CO_2_-adduct is reversible, however, when Et_3_SiH is added then it provided the [TMPH][HB(C_6_F_5_)_3_] salt along with a triethylsilyl carbamate 40.^78^

For this reaction, Wang and co-workers carried out computational studies to look at the mechanism of CO_2_ reduction to methane with Et_3_SiH catalysed by the ion pair [TMPH][HB(C_6_F_5_)_3_] in combination with B(C_6_F_5_)_3_ in detail. The mechanism proposed by Piers was confirmed to be energetically feasible in this study. The reduction proceeds *via* CO_2_ insertion into [TMPH][HB(C_6_F_5_)_3_], followed by three successive hydride transfers from Et_3_SiH to the CO_2_ centre. It was confirmed that the insertion of CO_2_ into the H–B bond of [TMPH][HB(C_6_F_5_)_3_] proceeds in a stepwise manner with H^δ+^ and H^δ−^ in the salt first transferring to CO_2_ to form 41 (Scheme 27, insert).

The role of B(C_6_F_5_)_3_ was also found to be important since it promotes hydride transfer and acts as a shuttle to bring H^δ−^ from Et_3_SiH to CO_2_.^79^ Overall, additional B(C_6_F_5_)_3_ activates the silane reducing agent, Et_3_SiH, producing Et_3_Si^+^ as a good oxygen acceptor and thus promotes the catalytic deoxygenation of CO_2_ to CH_4_.

**Scheme 28:**
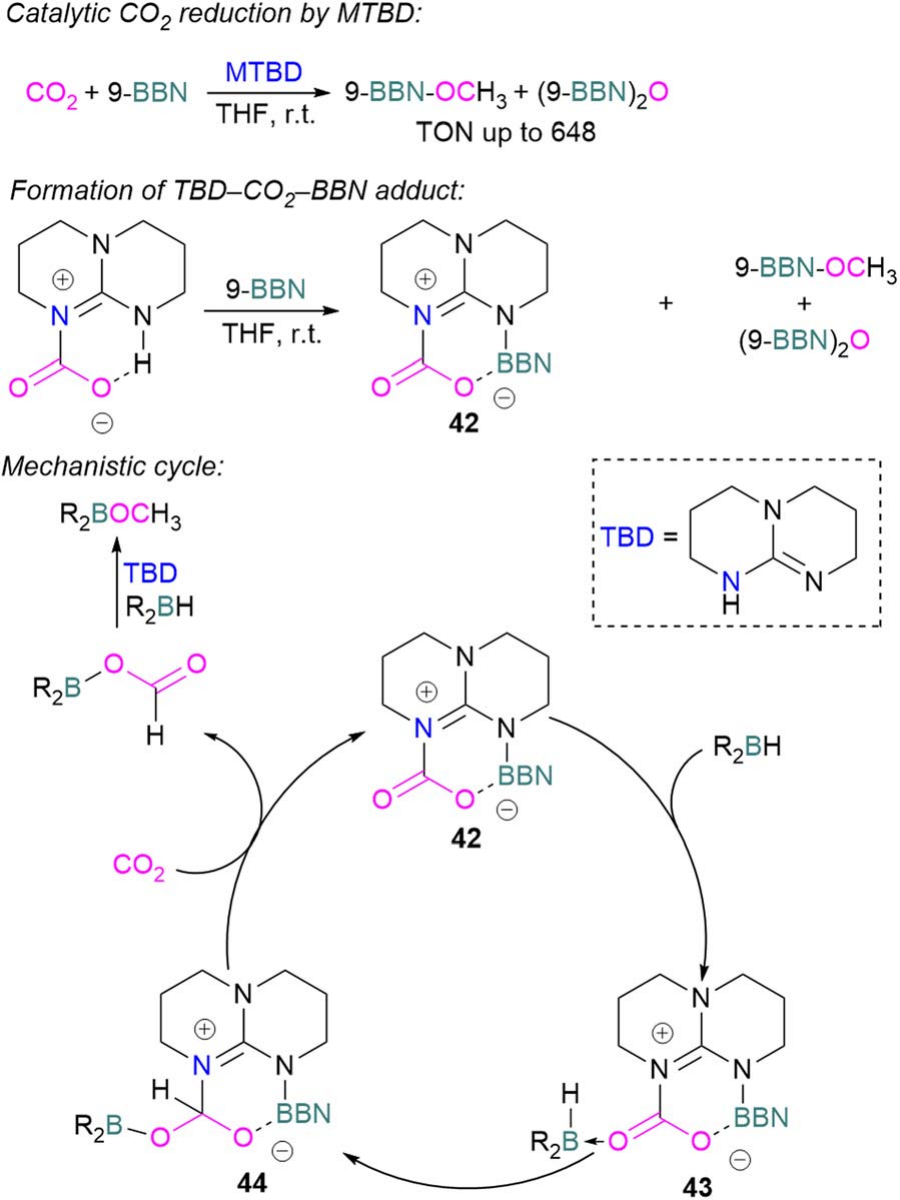
Catalytic reduction of CO_2_ using N-bases and B–H reducing agents.

Cantat and co-workers explored nitrogen bases such as TBD (triazabicyclodecene), Me-TBD (MTBD), DBU (1,8-diazabicyclo[5.4.0]undec-7-ene), and others for the reduction of CO_2_ in the presence of 9-BBN or CatBH. The reactions were performed at room temperature and a TON of up to 648 was achieved (Scheme 28). In this process, CO_2_ is initially reduced to a borylformate which then undergoes reduction firstly to an acetal and then a methoxyborane. The stoichiometric reaction of TBD–CO_2_ and 9-BBN in THF forms product 42 along with other reduced products (Scheme 28). Compound 42 was analysed by single crystal X-ray diffraction and it was found that the acidic NH proton in the TBD–CO_2_ adduct was replaced with a 9-BBN unit. Compound 42 can be considered as a nitrogen/boron FLP system trapped with CO_2_. A mechanism was proposed based on rigorous control experiments. In 42, CO_2_ behaves as a Lewis base and coordinates to the hydroborane R_2_BH to form adduct 43, which enables hydride transfer from the borane to carbon and forms 44. Compound 42 is regenerated when CO_2_ is applied, releasing the boron formate and thus catalysed the system for CO_2_ hydroboration. Finally, the boron formate is reduced to the methoxyborane. It is important to note that for MTBD it was found that the reaction proceeds with the activation of borane followed by the capture of CO_2_.^80^

**Scheme 29:**
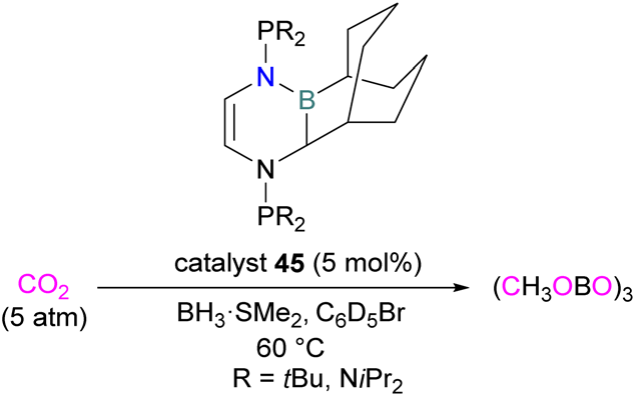
Catalytic reduction of CO_2_ using FLP 45.

Stephan and co-workers inadvertently discovered a new class of N/B molecule 45, that consists of a strong Lewis basic phosphorus centre and weak Lewis acidic boron centre which makes it a suitable FLP system. They utilised FLP 45 in the reduction of CO_2_ (5 atm) at 60 °C with BH_3_·SMe_2_ as a reducing agent and obtained a boroxine product (Scheme 29).^81^ The reduction of CO_2_ was observed catalytically in this case due to the presence of a strong basic centre and a weak Lewis acid that facilitates lability of the reduced CO_2_ fragments. This shows a difference to FLPs composed of a strong Lewis acid in which only stoichiometric reduction was observed, as in the case of 18 where 1,2-C_6_H_4_(BCl_2_)_2_ is the Lewis acid (Scheme 11).

**Scheme 30:**
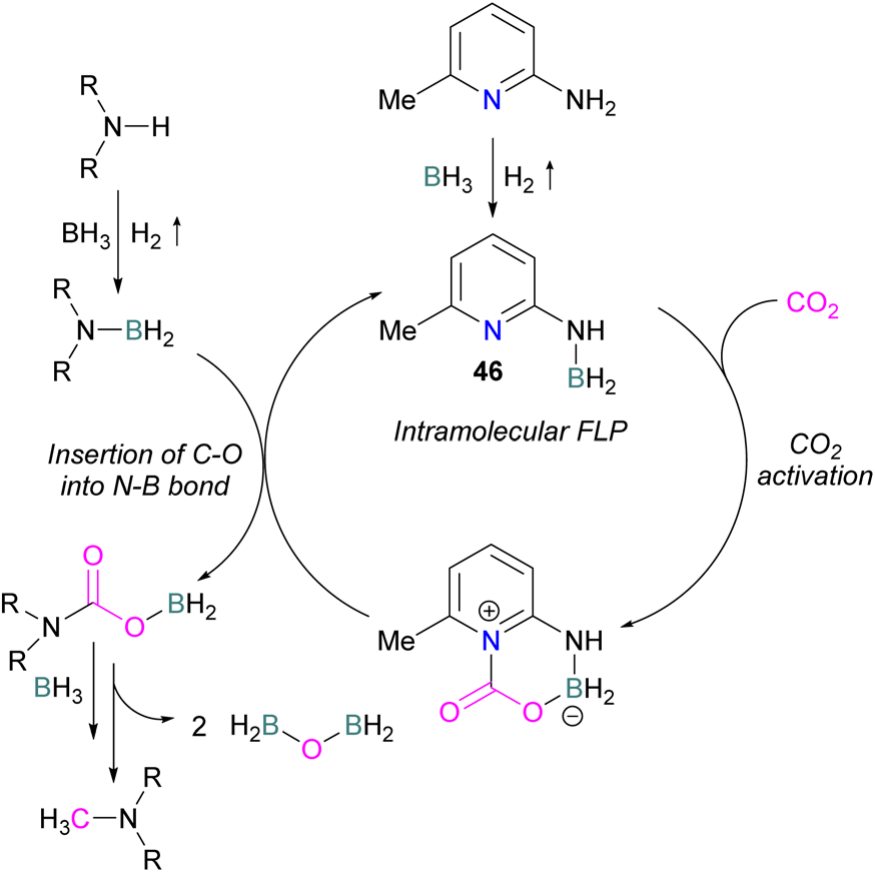
Catalytic transformation of CO_2_ by *N*-methyl formylation of secondary amines.

Zhang *et al.* found that 4 equivalents of BH_3_·NMe_3_ and catalytic 6-amino-2-picoline could be used to formylate secondary amines. The proposed mechanism proceeds though dehydrocoupling of the amineborane and catalyst to form an intramolecular FLP 46, which reacts with CO_2_. The activated CO_2_ is then inserted into the N–B bond which is subsequently reduced by borane with loss of H_2_BOBH_2_ to give the methylated amine (Scheme 30).^82^

For the activation of CO_2_ using FLPs, many arrangements of plausible Lewis pairs are possible. Hence, it is a challenge to find a particular combination that is superior for catalysing CO_2_ reduction.

**Scheme 31:**
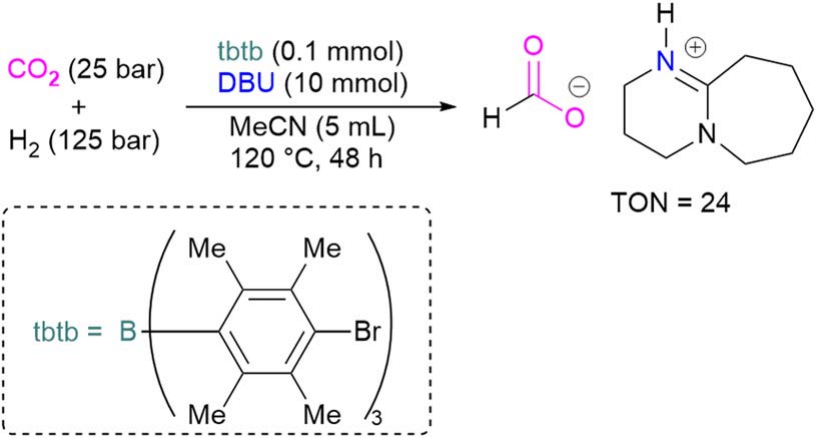
Catalytic transformation of CO_2_ to formate using the inverse FLP consisting of tbtb and DBU.

With this in mind, Corminboeuf and co-workers proposed a map of chemical composition of FLPs for their activity towards formate product by catalytic hydrogenation of CO_2_. They built the map upon linear scaling relationships, pinpointing specific FLP combinations with complementary acidity and basicity to optimally balance the energetics of the catalytic cycle. Amongst such combinations, they created a library of 60 P/N Lewis bases and 64 triaryl boranes as Lewis acids resulting in a library of 3840 FLPs. Out of these, they experimentally demonstrated the catalytic transformation of CO_2_ to formate by using an inverse FLP system obtained from tris(*p*-bromo)tridurylborane (tbtb) as Lewis acid and DBU as the Lewis base. A turnover number of 24 ± 3 was found for this catalytic reaction (Scheme 31). This is the first example of a metal-free CO_2_ hydrogenation in which stoichiometric addition of a silylhalide was not required. This was achieved through the fine-tuning of the Lewis acid and base based on their energies of hydride and proton attachment, respectively. Here, the authors conclude that inverse FLPs, with a weaker Lewis acid and strong Lewis base or strong Lewis acid with weaker Lewis base, yet with cumulative high acid–base strength, is the ideal combination to achieve CO_2_ hydrogenation. The authors highlight the importance of overcoming both activation barriers to CO_2_ activation as well as H_2_ activation when targeting catalytic CO_2_ hydrogenation.^83^

**Scheme 32:**
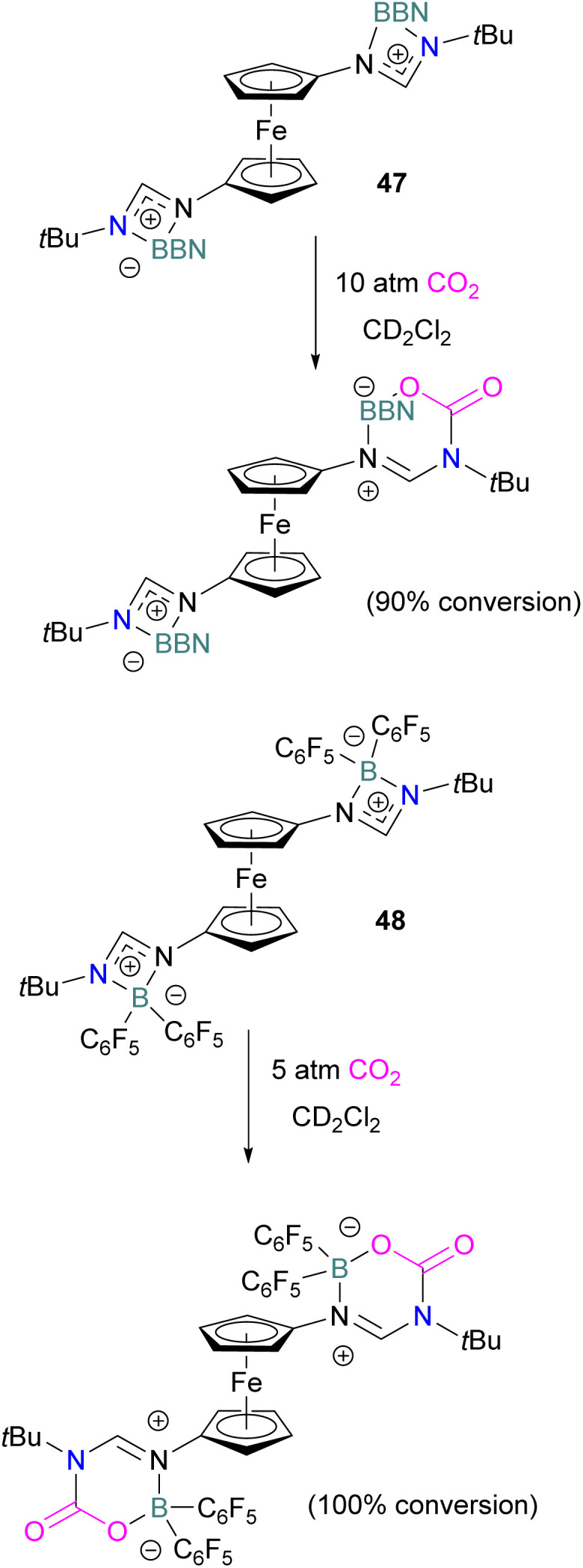
CO_2_ adducts of bis(boramidinate)ferrocenes.

In 2022, Palomero and Jones reported the preparation of bis(boramidinate)ferrocenes 47 and 48 by hydroboration of 1,1′-dicarbodiimidoferrocenes. The resulting compounds reacted with CO_2_. The reaction of the BBN derivative 47 with CO_2_ (10 atm) to form the mono-CO_2_-bound product as a yellow precipitate (90% conversion) in a process that was highly reversible (Scheme 32). Whilst this precluded isolation of the CO_2_-bound products, such reversibility may be preferable for applications in catalytic hydrogenation, facilitating release of the reduced product and catalytic turnover. Lower pressures of CO_2_ were shown to reduce conversion.^84^ To allow the use of lower pressures, the more electron-poor bis(pentafluorophenyl)borane analogue 48 was employed. At 5 atm CO_2_, it activated 2 equivalents of CO_2_, although the reaction required two weeks to go to completion.

### Borane/carbon FLPs for CO_2_ activation

**Scheme 33:**
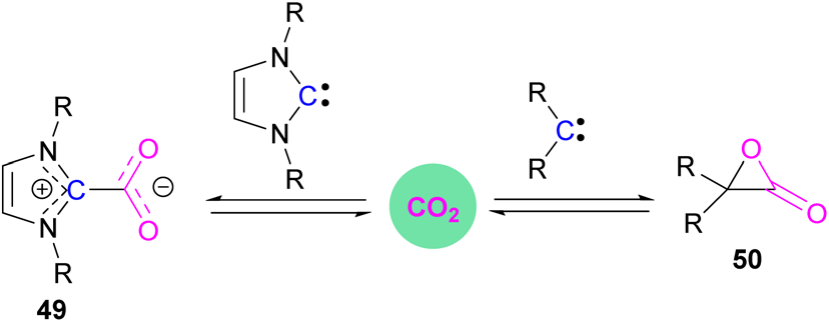
Reaction of carbenes with CO_2_.

Stable *N*-heterocyclic carbenes (NHCs) upon reaction with CO_2_ form a mesomeric betaine 49 having a C–C bond between the carbene and CO_2_. On the other hand, very reactive carbenes can form oxiranones 50 (Scheme 33). The product remains in equilibrium with the starting substrates. Most attention has been focused on stable sterically hindered NHCs amongst all carbenes for the activation of small molecules,^85^ and several carbenes in FLP systems have been explored and found to be efficient in the activation of CO_2_.^86^

**Scheme 34:**
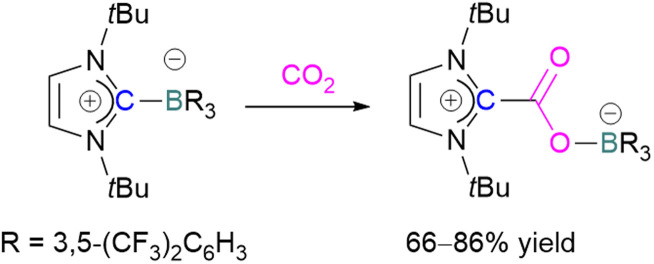
First carbene based FLP system for CO_2_ activation.

The first carbene based FLP system to activate CO_2_ was reported by Tamm and co-workers in 2012 (Scheme 34).^87^ They showed that exposure of CO_2_ to a solution of a bulky carbene (1,3-di-*tert*-butylimidazolin-2-ylidene) and tris[3,5-bis(trifluoromethyl)phenyl]borane, B(3,5-(CF_3_)_2_C_6_H_3_)_3_, in benzene at 60–70 °C, a white precipitate, identified as the FLP-CO_2_ adduct was formed. The adduct was isolated in 66% yield. At room temperature this adduct was also obtained on exposure of CO_2_ to the solution of the FLP in benzene with a 24 h reaction time and a higher yield of 86% was isolated.

**Scheme 35:**
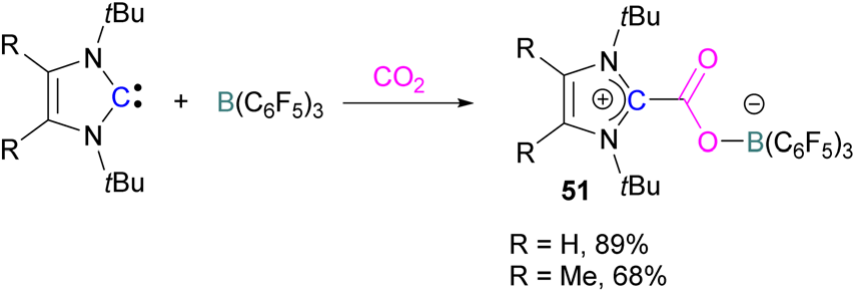
Capture of CO_2_ with NHC/B(C_6_F_5_)_3_ FLPs.

Later, Tamm and his group synthesised a library of carbene based FLP CO_2_-adducts and studied their reaction profile computationally (level of theory: M05-2X/6-311G**). A 1 : 1 mixture of a bulky carbene and B(C_6_F_5_)_3_ with CO_2_ provided NHC-CO_2_-B(C_6_F_5_)_3_ products 51 in 89% and 68% yield depending on the starting carbene (Scheme 35). From DFT calculations a low energy barrier was observed for the NHC-CO_2_-B(C_6_F_5_)_3_ adduct formation (10.4 (R = H) and 12.2 (R = Me) kcal mol^−1^), and it was concluded that steric changes on the NHC were more pronounced than electronic impacts.^88^

**Scheme 36:**
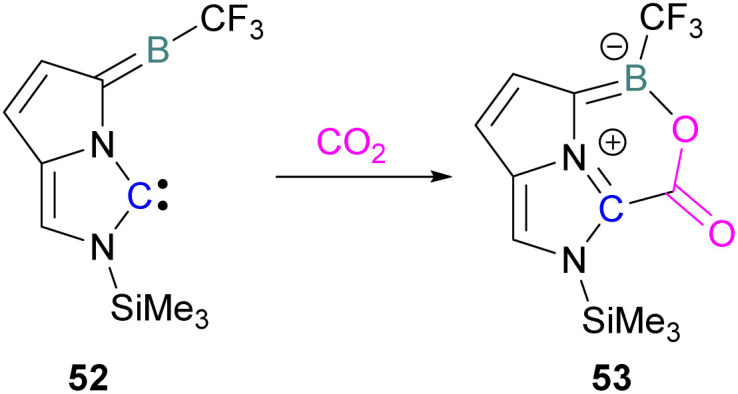
Computationally designed carbene/borane derived FLP.

**Scheme 37:**
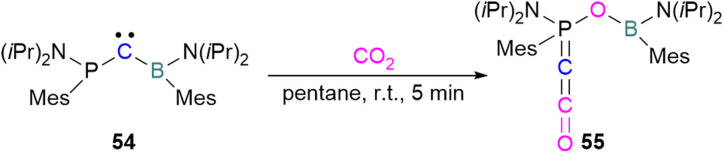
CO_2_ adduct of a boryl(phosphine)carbene.

Zhu and co-workers computationally designed a boron-based carbene intramolecular FLP 52 and calculated its reactivity with various small molecules, including CO_2_. This FLP with CO_2_ forms a zwitterionic species 53 and the authors discuss the important driving force of aromaticity in the final adduct (Scheme 36).^89^ Baceiredo and co-workers exposed boryl(phosphine)carbene 54 to CO_2_ (1 atm) and an unusual product 55 was observed. After analysis of the product's structure, it was found that the carbene inserted into the C=O bond of the CO_2_. Thus, incorporating carbon dioxide into the corresponding phosphoryl ketenylidene derivative (Scheme 37).^90^

**Scheme 38:**
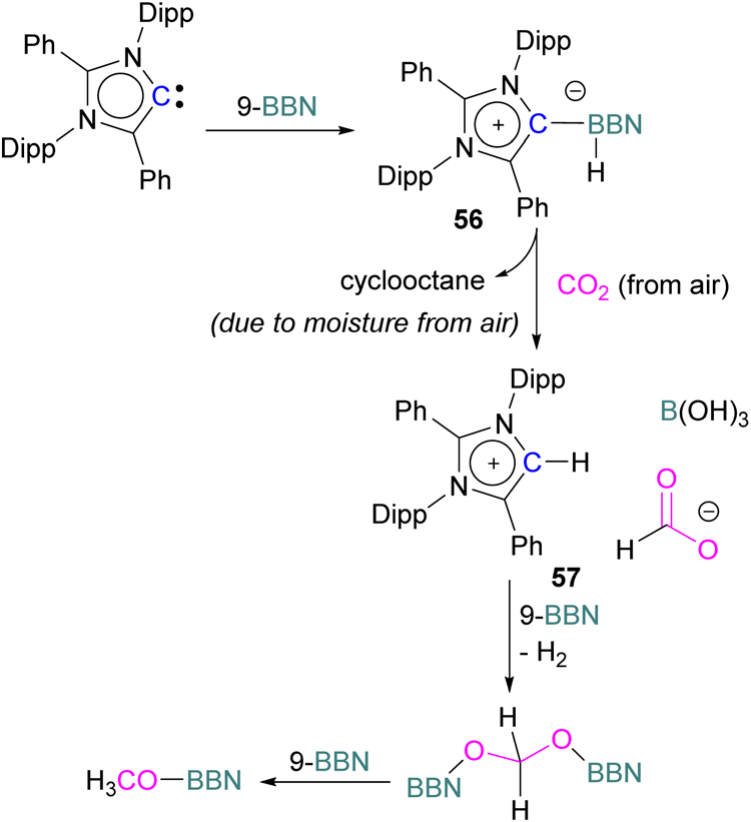
NHC-BBN adduct captures of CO_2_ from the air under ambient conditions and reduction to formate and methoxide.

**Scheme 39:**
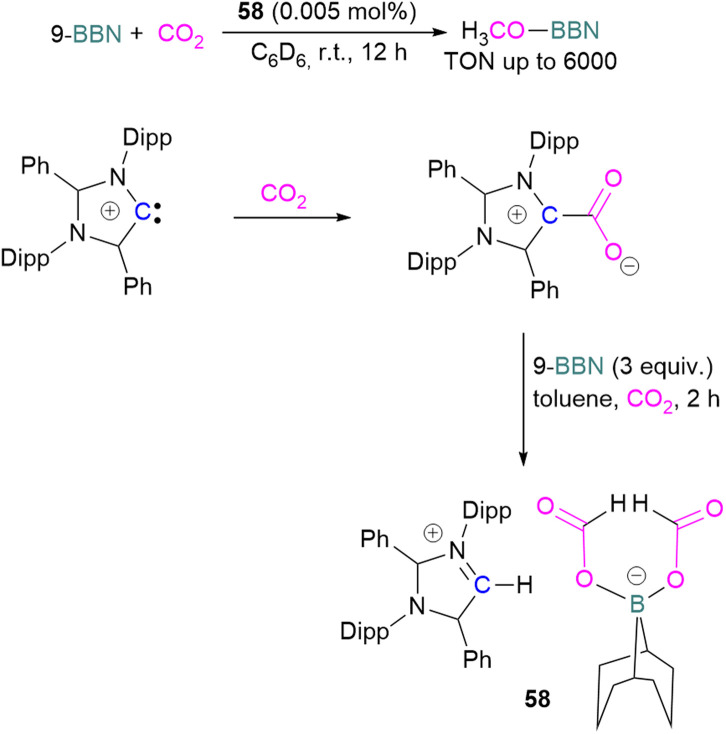
Preparation of zwitterionic boron diformate 58 and its use as a catalyst for the reduction of CO_2_.

Stoichiometric reduction reactions with carbene/borane FLPs have been reported.^91^ In 2019, Mandal and co-workers prepared an *N*-heterocyclic carbene-boron adduct 56 by reacting an abnormal heterocyclic carbene (aNHC) with 9-BBN. The synthesised NHC-boron adduct 56 was utilised to capture CO_2_ from the atmosphere under ambient conditions in benzene overnight. Product 57 was obtained, due to moisture in the air leading to hydrolysis of 9-BBN and boric acid formation with the release of a cyclooctane molecule. The CO_2_ was incorporated as a formate ion. Further treatment of 57 with excess 9-BBN leads to the formation of compound CH_2_(OBBN)_2_ with the release of H_2_, and finally converts this to CH_3_OBBN (Scheme 38).^91^ This work was presented as a first metal-free system to reduce CO_2_ by capturing it from the atmosphere under ambient conditions where CO_2_ remains in a concentration of ∼400 ppm. Mandal and co-workers later reported the use of the same FLP system for a catalytic reduction of CO_2_ in the presence of a range of hydroboranes leading to methoxyborane (Scheme 39). Reaction of the carbene with CO_2_ firstly gave the adduct whilst reaction of the carbene with 3 equivalents of 9-BBN in the presence of CO_2_, provided boron diformate 58. Zwitterionic boron diformate 58 was utilised catalytically with a loading of 0.005 mol% for the conversion of 9-BBN to the methoxide derivative CH_3_O-BBN under a CO_2_ atmosphere. Catalyst 58 leads to a TON of 6000, which is the highest TON observed among all the metal-free catalysts investigated at ambient conditions. The key feature of this catalytic process is the formation two equivalents of 9-BBN formate, BBN(OCHO), from the reaction of catalyst 58 with an equivalent of 9-BBN resulting in the release of dihydrogen.

This generates the carbene which further captures a CO_2_ molecule regenerating 58 with 9-BBN and thus providing a catalytic process. The 9-BBN formate is finally reduced and hydroborated to CH_3_O–B in a series of steps in the presence of an excess of 9-BBN.^92^

### Borane/silicon FLPs for CO_2_ activation

**Scheme 40:**
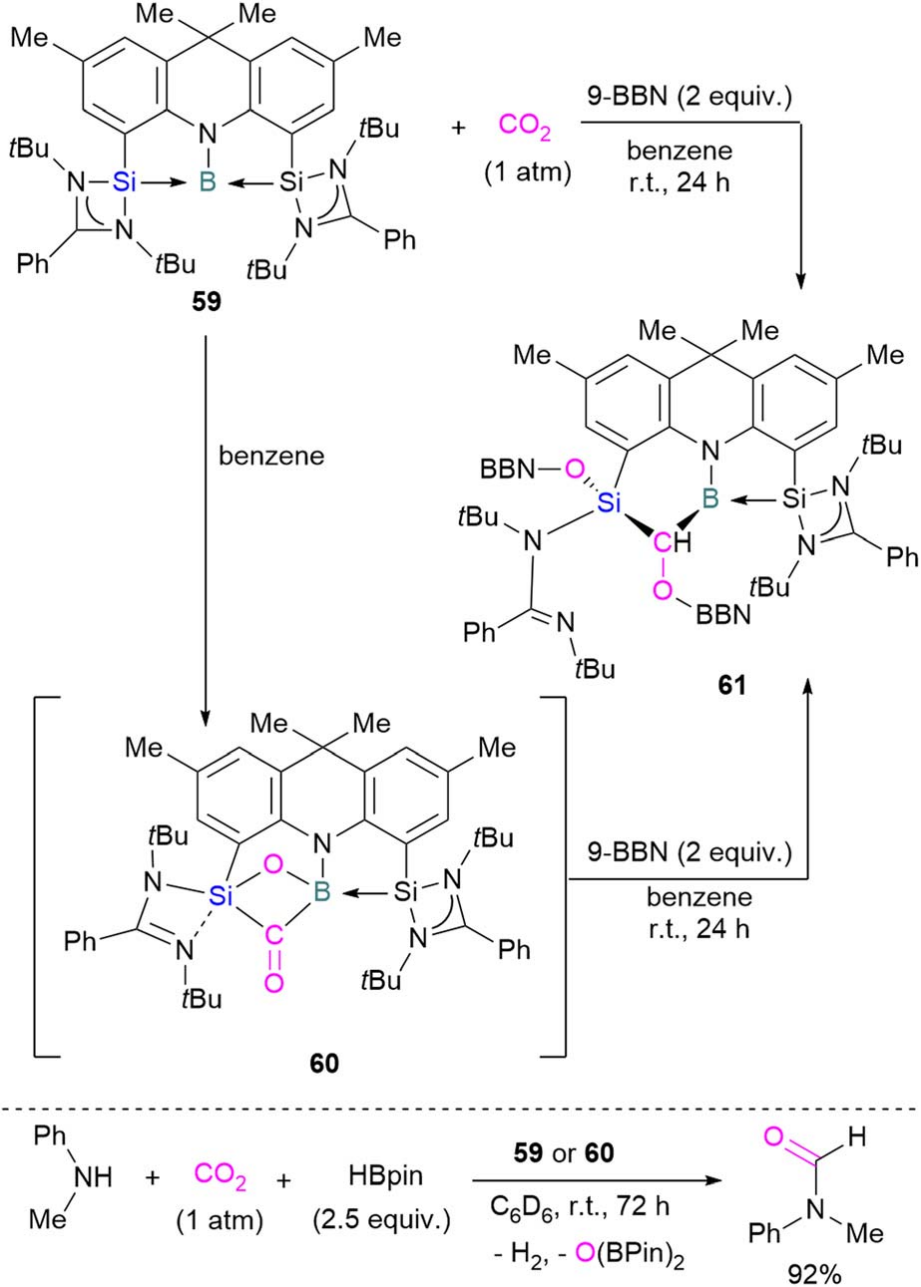
Cooperative bond activation of CO_2_ with a borylene/silylene compound and catalytic application.

To date there is just one example of a boron/silicon FLP for CO_2_ activation. Very recently Mo and co-workers reported the synthesis of a geometrically constrained bis(silylene)-stabilised borylene 59.^93^ Spectroscopic and X-ray analyses reveal that structure 59 has a tricoordinate boron centre with a distorted T-shaped geometry. Computational analysis shows that the HOMO comprises a lone pair of electrons on the boron centre and is delocalised over the Si–B–Si unit. Compound 59 shows single electron transfer reactivity towards B(C_6_F_5_)_3_ forming a frustrated radical pair [(SiNSi)B]˙^+^[B(C_6_F_5_)_3_]˙^−^. The reaction of 59 with CO_2_ (1 atm) in C_6_D_6_ at room temperature forms a new product 60 by cleaving CO_2_ which, upon hydroboration with two equivalent of 9-BBN, forms compound 61 in quantitative yields. The structure of 61 showed that boron and silicon atoms are bridged by boryloxymethylene (CHOBR_2_) formed by the hydroboration of the C=O group. The Si–O–B bridge in 60 was cleaved along with the formation of BH and SiOBR_2_ units. Compound 61 can also be obtained directly by treating 59 with CO_2_ and 2 equivalents of 9-BBN in C_6_D_6_ at room temperature with an isolated yield of 45% (Scheme 40). The catalytic performance of 59 (5 mol%) and 60 (5 mol%) shows an efficient transformation of *N*-methylaniline into the corresponding formamide (92% yields in each case) by capturing and hydroborating CO_2_ with HBpin (Scheme 40).^93^

### Borane/germanium FLPs for CO_2_ activation

**Scheme 41:**
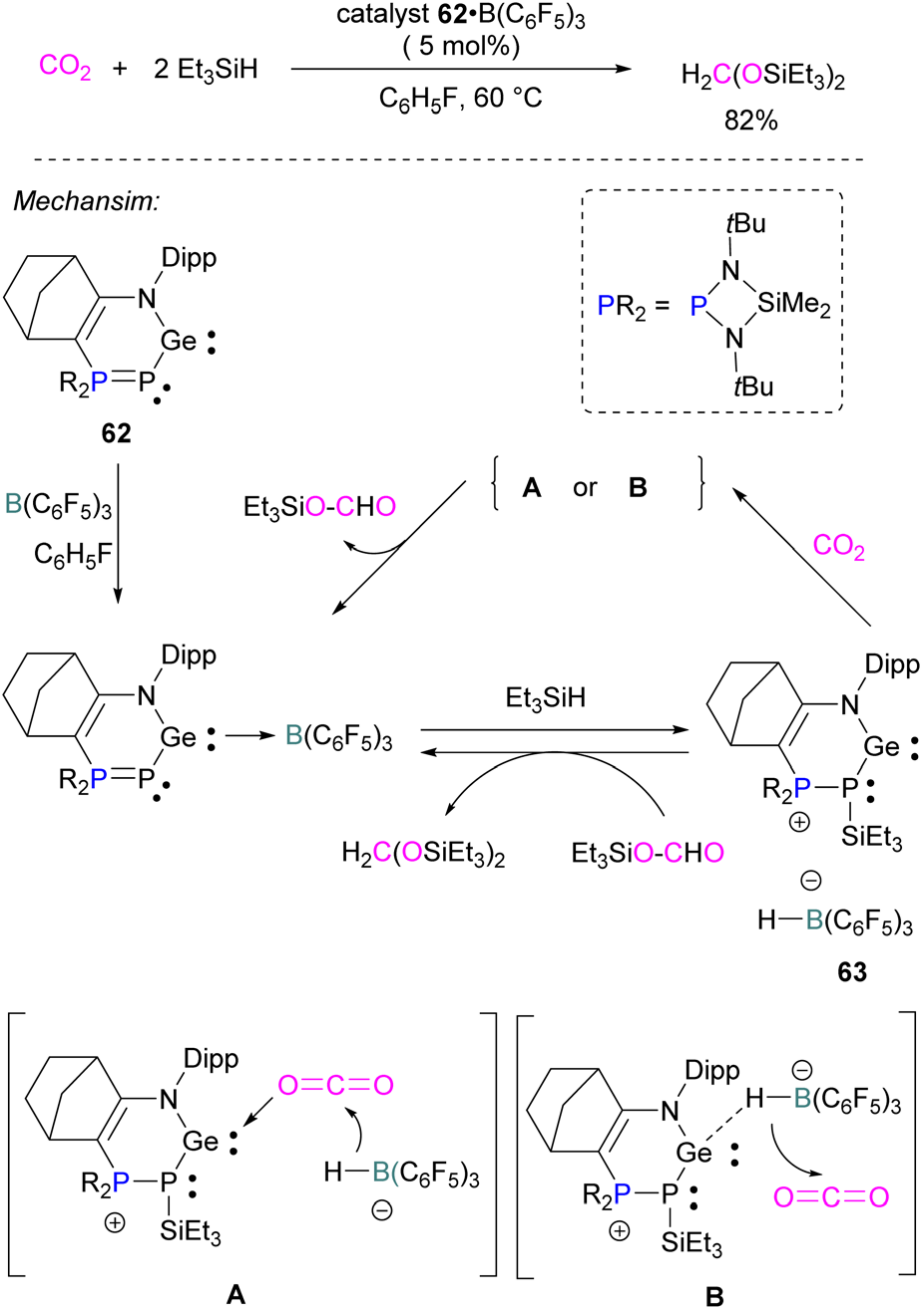
*N*,*P*-heterocyclic germylene in catalytic hydrosilylation of CO_2_.

Similar to the silylene described above, germylenes are also reported as the Lewis base component of an FLP for CO_2_ reduction. Kato and co-workers reported an interesting *N*,*P*-heterocyclic germylene 62 in 2016, that bears several reactive sites (including a germylene centre) and can activate two CO_2_ molecules simultaneously.^94^ Compared to classical FLPs, 62 showed unusual behaviour of multi-reactive sites and has been utilised as a donor component in the Lewis acid–base pair. In fluorobenzene, the *N*,*P*-heterocyclic germylene 62 reacts with B(C_6_F_5_)_3_ at room temperature to give the corresponding adduct 62 B(C_6_F_5_)_3_ as colourless crystals in 67% yield (Scheme 41). The authors explored the catalytic reduction of CO_2_ with 5 mol% of the FLP adduct 62 B(C_6_F_5_)_3_ and the reducing agent Et_3_SiH. The proposed mechanism for CO_2_ activation showed that 62 B(C_6_F_5_)_3_ reacts with silane Et_3_SiH and forms a cationic germylene 63 which promotes CO_2_ hydrosilylation catalytically *via* two possible activation modes A and B to obtain product H_2_C(OSiEt_3_)_2_ selectively.^94^

### Borane/boron FLPs for CO_2_ activation

FLP systems where the same element is used both as the Lewis acidic and Lewis basic reactive centres are rarely observed. One such example was reported by Kinjo and co-workers in 2015 with 1,3,2,5-diazadiborinine 64 featuring nucleophilic and electrophilic boron centres within the same molecule.^95^

**Scheme 42:**
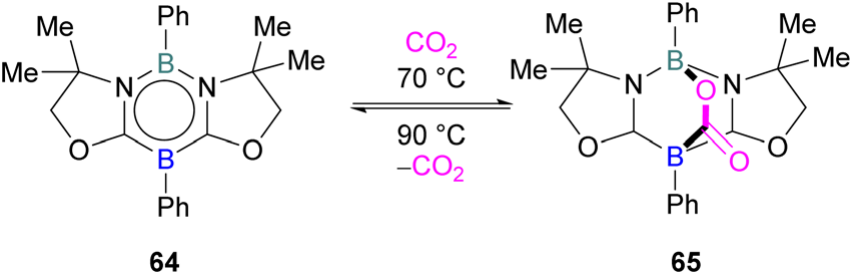
Reaction of a 1,3,2,5-diazadiborinine with CO_2_.

This first reported intramolecular boron–boron FLP showed high regioselectivity in the reaction with CO_2_, yielding a bicyclic product 65 (Scheme 42). Interestingly, CO_2_ activation by 64 was reversible at 90 °C. To obtain insight into electronic features of 1,3,2,5-diazadiborinine 64, the authors performed DFT calculations [level of theory: B3LYP/6-311G+(d,p)]. NBO analysis showed that the compound possess both nucleophilic and electrophilic boron centres with a formal B(+I)/B(+III) mixed valence system.^95^ Later, Zhao and co-workers performed more detailed computational analyses of 64.^96^ They reported π delocalisation over the central ring which extends from the lone pair on (O) → π*(N–C), and favourable orbital overlap with CO_2_ is generated from the electrophilic interaction with the Lewis acidic boron centre and nucleophilic donation to the LUMO+3 of the other boron centre.

Kinjo and co-workers synthesised another class of boron compounds in which the boron acts as a Lewis basic centre.

**Scheme 43:**
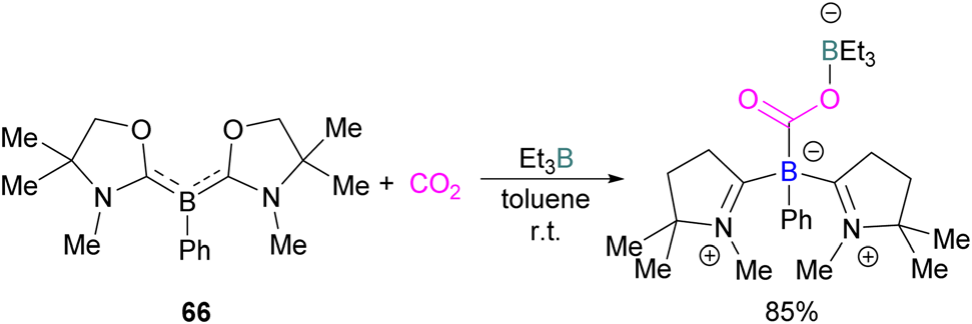
Reaction of tricoordinate organoboron compound with Et_3_B and CO_2_.

It was found previously that tricoordinate organoboron L_2_PhB: (L = oxazol-2-ylidene) compound 66 does not react with BEt_3_ This is perhaps due to a mismatch of the softness/hardness of the respective boron centres in 66 and BEt_3_ based on HSAB (Hard Soft Acid Base) theory in addition to steric hindrance. As compound 66 and BEt_3_ do not react, they act like an FLP. Thus 66 and BEt_3_ were reacted with CO_2_ in toluene at room temperature and the FLP-CO_2_ adduct was isolated in 85% yield (Scheme 43).^97^

### Borane/metal FLPs for CO_2_ activation

**Scheme 44:**
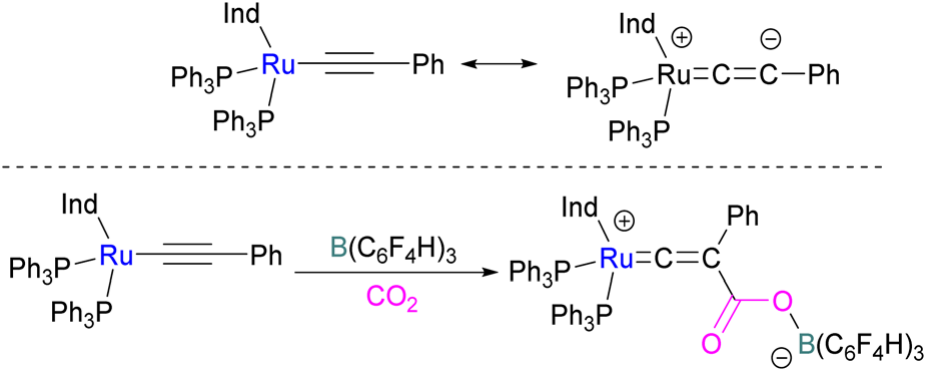
CO_2_ adduct of [(η^5^-indenyl)Ru(PPh_3_)_2_(CCPh)] and B(C_6_F_4_H)_3_. Ind = Indenyl.

In addition to p-block Lewis bases, transition metal complexes can also act as the Lewis base component of an FLP with boron as the Lewis acid. This is demonstrated using a ruthenium acetylide which is an electron rich species. This can create a Lewis basic β-carbon centre and form an FLP when combined with a Lewis acid (Scheme 44). Stephan and co-workers in 2013 showed that when [(η^5^-indenyl)Ru(PPh_3_)_2_(CCPh)] was reacted with B(*p*-C_6_F_4_H)_3_ no reactivity was observed, indicating that their combination is an FLP in nature. A solution of this FLP, when exposed to CO_2_ for 12 h, provided an orange solid in 70% yield and was fully characterised using NMR and single crystal X-ray crystallography as the FLP-CO_2_ adduct where the β-carbon centre had attacked the electrophilic carbon centre of CO_2_ (Scheme 44).^98^

**Scheme 45:**
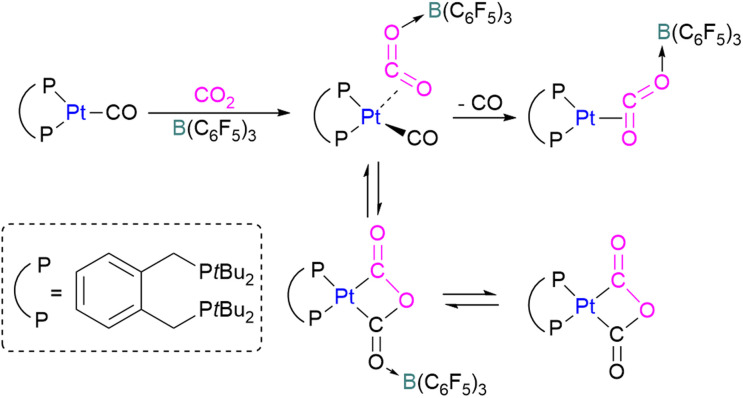
Activation of CO_2_ using Lewis basic platinum(0)–CO complex/B(C_6_F_5_)_3_.

Wass and co-workers explored reactions with a platinum(0) complex as a Lewis base in the activation of small molecules using a sterically congested boron-based Lewis acid. They found that pairing a Lewis basic platinum(0)–CO complex supported by a diphosphine ligand with B(C_6_F_5_)_3_ acts as a frustrated Lewis pair, to activate CO_2_ (Scheme 45). The presence of B(C_6_F_5_)_3_ is important as no activity was observed between the platinum complex and CO_2_ in the absence of the borane. In this scenario, Pt(0) acts as a donor of electron and the boron atom acts as the acceptor forming a coordinated Pt–CO_2_–B system. A substitution of CO by CO_2_ on platinum was observed after the loss of the CO molecule.

In this process 95% isotopically pure ^13^CO_2_ was used but the ^31^P NMR analysis of the product showed a mixture of ^13^C labelled and unlabelled product in a ratio of 4 : 1. The source of unlabelled product must be from the ^12^CO in ligand of the starting material. This suggests that a symmetrical [C_2_O_3_]^2−^ complex forms during the reaction pathway. The proposed mechanism in Scheme 45 suggests that the reaction of the platinum(0)–CO complex and B(C_6_F_5_)_3_ with CO_2_ is a metal-mediated oxygen transfer between CO_2_ and CO rather than a simple ligand substitution.^99^

**Scheme 46:**
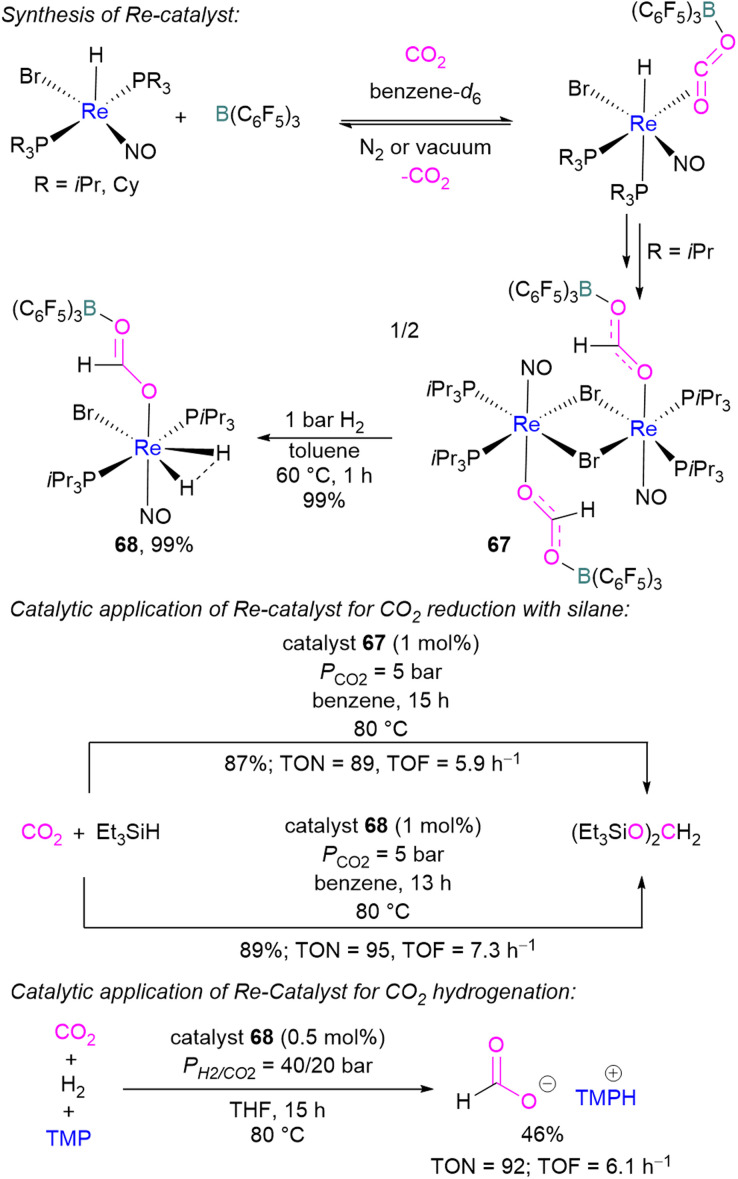
Synthesis of [Re]-CO_2_-B(C_6_F_5_)_3_] adduct and utilisation as a catalyst in CO_2_ reduction.

In 2013, Berke and co-workers showed an FLP-type activation of CO_2_ using a [Re]–H/B(C_6_F_5_)_3_ system where the Re–H bond acts as a Lewis base. Catalysts 67 and 68 were prepared stepwise from a rhenium hydride precursor [ReH(PR_3_)_2_(NO)Br] (Scheme 46). Initially, the precursor was reacted with B(C_6_F_5_)_3_ and CO_2_ in benzene to form the FLP-CO_2_ adduct. With a P*i*Pr_3_ ligand on the rhenium precursor, insertion of the Re–H into the FLP-bound CO_2_ molecule was observed generating 67. Compound 67 could be hydrogenated with H_2_ (1 bar) in toluene at 60 °C for 1 h to give 68 in 99% yield. Both 67 and 68 were screened for the hydrosilylation of CO_2_ using Et_3_SiH as a reducing agent (Scheme 46). Catalyst 67 with a loading of 1 mol% provided the (Et_3_SiO)_2_CH_2_ product in 87% yield (TON = 89, TOF = 5.9 h^−1^), while 68 provided the reduced product in 89% yield (TON = 95, TOF = 7.3 h^−1^). Similarly, catalysts 67 and 68 were utilised for CO_2_ hydrogenation (*P*_H_2_/CO_2__ = 40/20 bar) in the presence of TMP as a base. Catalyst 68 provided the formate salt of TMP in 46% yield (TON = 92, TOF = 6.1 h^−1^).^100^

**Scheme 47:**
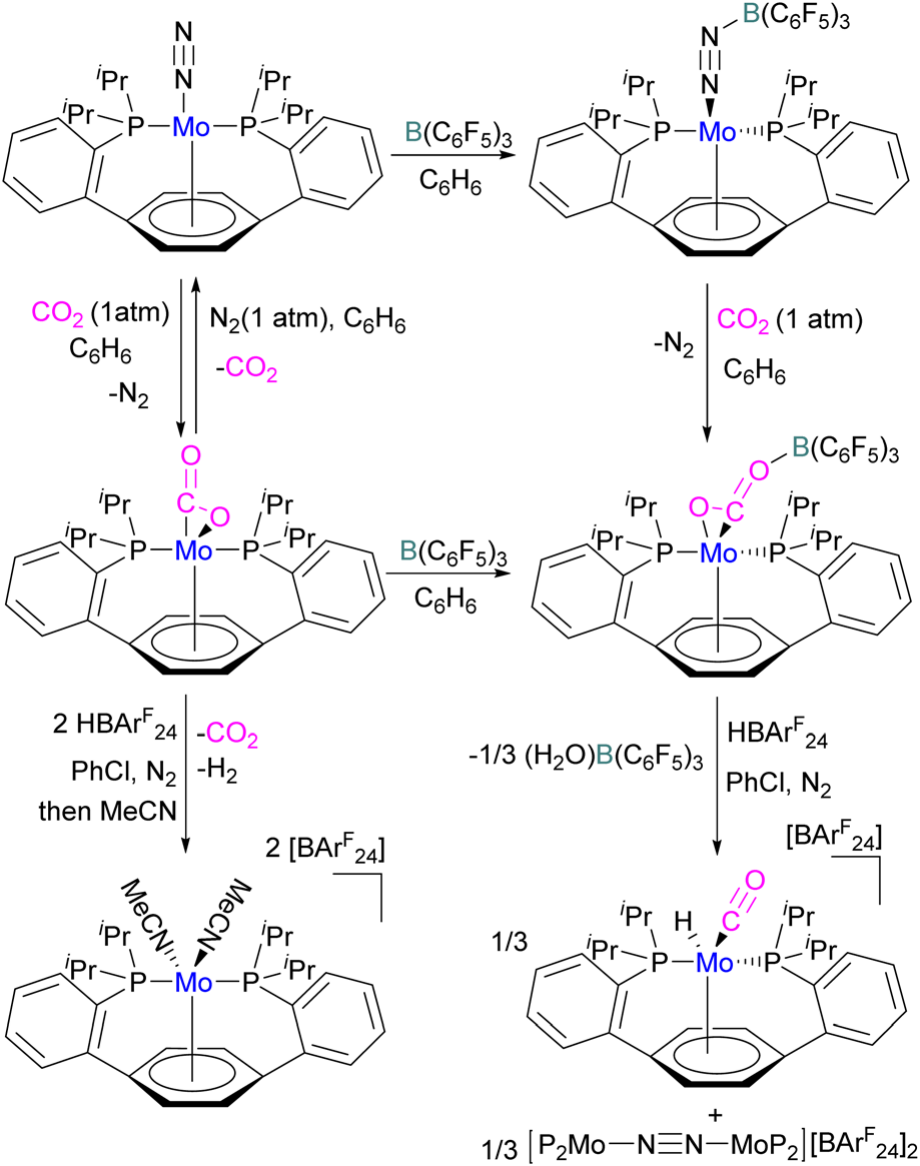
Mo based FLP for CO_2_ activation. BAr^F^_24_ = tetrakis(3,5-bis(trifluoromethyl)phenyl)borate.

In another study, Agapie and co-workers investigated the effects of the Lewis acid B(C_6_F_5_)_3_ towards the conversion of CO_2_ to CO and water using a molybdenum complex (Scheme 47).^101^ The activation of CO_2_ was found to be linearly related to the strength of the Lewis acid. When a labile Mo(0)-CO_2_ adduct interacts, it will increase both the degree of activation and the kinetic stability of bound CO_2_ as shown in Scheme 47. In contrast to the CO_2_ displacement by a solvent that is predominantly observed in the absence of a Lewis acid, in the presence of B(C_6_F_5_)_3_ and [H(Et_2_O)_2_][BAr^F^_24_] (BAr^F^_24_ = tetrakis[3,5-bis-(trifluoromethyl)phenyl]borate) CO_2_ cleavage occurs. This demonstrates the significance of kinetic and thermodynamic aspects in the effective CO_2_ reduction chemistry primarily relying upon bond activation and the residence period of the associated small molecule.

The authors demonstrated that the chemistry of the labile substrate is greatly influenced by the time the substrate resides in the metal's coordination sphere. It is shown that Lewis acid additives promote CO_2_ cleavage *via* kinetic stabilisation rather than merely by thermodynamic activation.

One final system to note here uses the boron Lewis acid B(C_6_F_5_)_3_ with s-block metal carbonates M_2_CO_3_ (M = Na, K, and Cs) for the highly efficient reduction of CO_2_ to formate. Amongst the screened metal carbonates, Cs_2_CO_3_ showed the highest TON of 3941.^102^

### Aluminium FLPs for CO_2_ activation

A number of FLPs based on aluminium Lewis acids have also been reported for CO_2_ capture and reduction, although the greater oxophilicity of aluminium (potentially inhibiting product release) means that catalytic hydrogenation has yet to be reported. This oxophilicity also means that, whereas FLPs containing boron Lewis acids typically bind CO_2_ in a 1 : 1 : 1 Lewis acid : Lewis base : CO_2_ ratio, Al-containing FLPs often bind it in a 2 : 1 : 1 ratio, with both oxygen atoms binding an aluminium centre.^103^ Studying FLPs comprised of phosphines and aluminium esters, Smythe *et al.*, showed that the ratio of mono-to bis-bound adduct varies with Lewis acidity.^104^

After exposure to 1 atm of CO_2_, Al(O–C_6_H_2_Cl_3_)_3_ bound CO_2_ in a predominantly mono fashion (*ca.* 95% mono), whereas the greater Lewis acidity of Al(OC_6_Cl_5_)_3_ (*ca*. 1 : 1 mono : bis) and Al(OC_6_F_5_)_3_ (*ca.* 75% bis) favoured the bis-bound adduct.

**Scheme 48:**
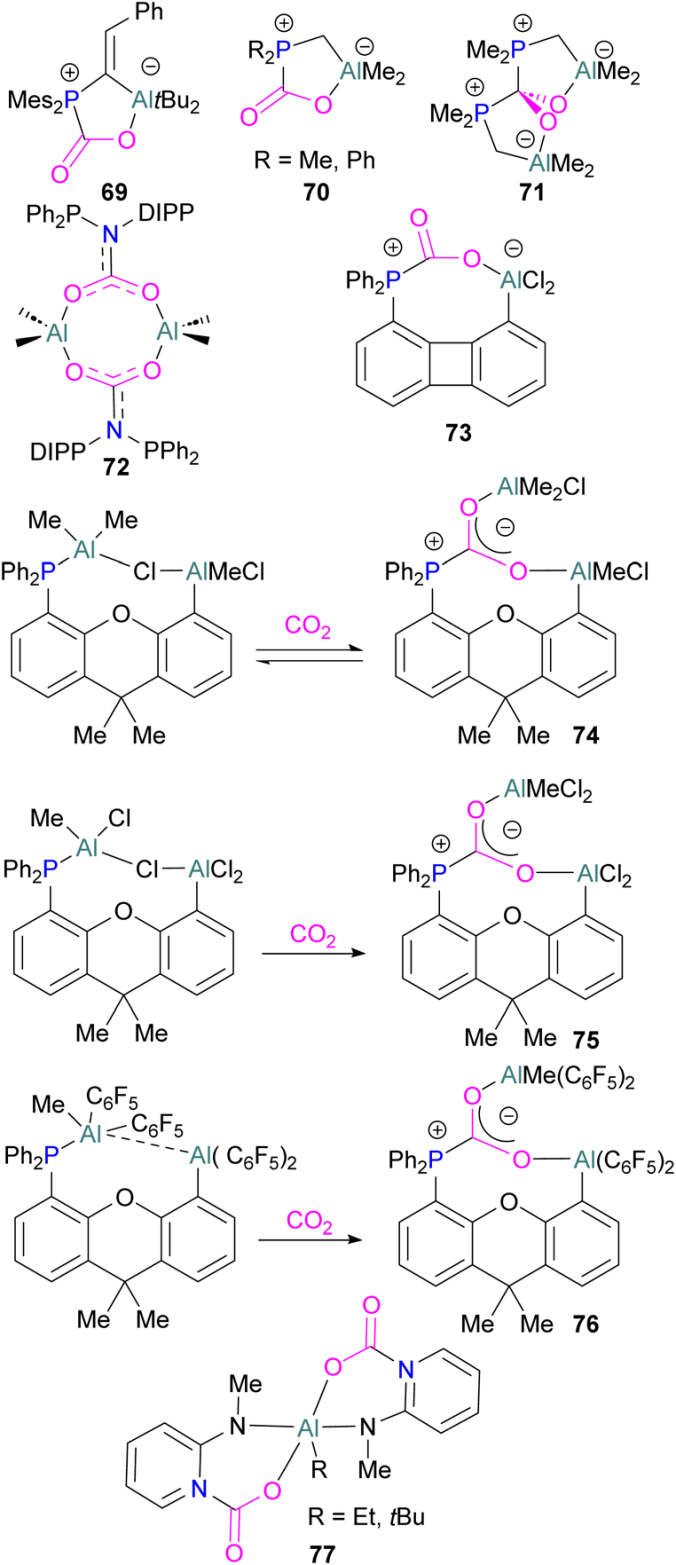
Aluminium based CO_2_ adducts.

Like boron CO_2_ adducts, a range of aluminium FLP-CO_2_ adducts are reported (Scheme 48). Uhl and co-workers reported the synthesis of geminal ambiphilic phosphine–aluminium FLPs that can activate CO_2_ to a form a cyclic adduct 69.^105^ Later, the same authors synthesised AlPC_2_O type heterocycle having *cis*/*trans* isomeric compounds.^106^ Uhl also reported a P–H functionalised Al/P FLP in 2019. The FLP reacts with CO_2_ to give a five-membered zwitterionic cycle similar to that in 69, as typical for vicinal intramolecular FLPs. However, the enhanced acidity of the phosphine means that it can be deprotonated by addition of a base (such as DABCO or *n*BuLi) to give a more stable precipitate.^107^ Similarly, Fontaine and co-workers studied the reactivity of the stable Lewis adducts [R_2_PCH_2_AlMe_2_ (R = Me, Ph)] and found adducts 70 and 71.^108^ Harder and co-workers reported a geminal Al/P FLP, with a nitrogen rather than the more common carbon linker. Like the carbon-linked Al/P FLP reported earlier by Fontaine, this reacts with CO_2_ to give a 2 : 2 eight-membered ring product 72 by insertion of CO_2_ into the Al-linker bond with *cis*- and *trans*-isomers.^109^ Limberg *et al.* utilised a biphenylene backbone to prepare a strained intramolecular P/Al-based FLP which was reacted with CO_2_ (2 bar) at room temperature for 5 minutes in deuterated dichloromethane to obtain adduct 73.^110^ They also reported xanthene-linked intramolecular Al/P FLPs containing two Al centres which are able to activate CO_2_.^111^

In the products 74–76, the two aluminium centres each bind to one of the CO_2_'s oxygen atoms. The binding strength could be tuned by varying the substituents on aluminium. The more Lewis acidic xanthene-AlCl_2_ and xanthene-Al(C_6_F_5_)_2_ fragments bind CO_2_ irreversibly giving 75 and 76 (Scheme 48), while xanthene-MeClAl binds CO_2_ reversibly giving 74 under 2 bar CO_2_, liberating CO_2_ when this excess pressure was released. For catalytic applications, this reversible binding is necessary to enable release of the product. Although most CO_2_-binding Al FLPs are Al/P rather than Al/N, one example of an Al/N FLP was described by Brewster in 2020 using the readily available 2-(methylamino)pyridine as ligand yielding 77 upon reaction with 2 equivalents of CO_2_.^112^ An example of an FLP-CO_2_ adduct with a metal as a Lewis base was provided by Bourissou and co-workers who utilised geminal P–Al ligand [Mes_2_PC(=CHPh)Al*t*Bu_2_/Pt(PPh_3_)] in the activation of CO_2_ molecule to obtain an adduct.

**Scheme 49:**
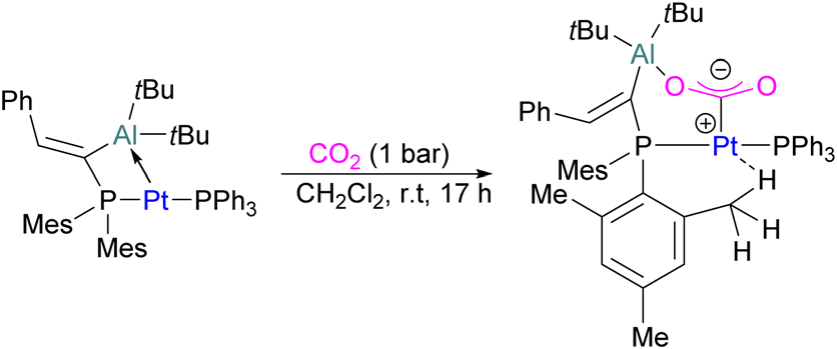
Pt/Al bimetallic FLP system in CO_2_ activation.

In this bimetallic system, platinum acts as the Lewis base and activates the CO_2_ molecule by reacting at the carbon centre of the CO_2_ molecule and the formed negative charge on one of the oxygen atoms is stabilised by the Lewis acidic aluminium centre (Scheme 49).^113^

**Fig. 7:**
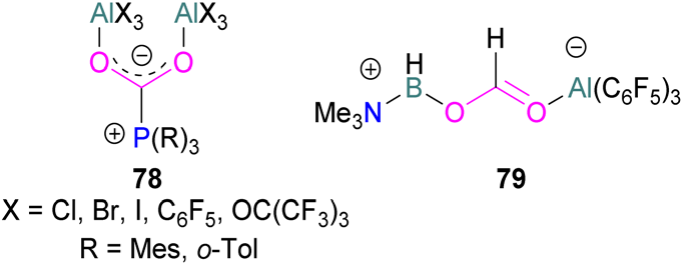
Aluminium based FLPs-CO_2_ adducts used in CO_2_ reduction.

Several of these adducts have been used in stoichiometric and catalytic transformations of CO_2_. Stephan and co-workers, reported the synthesis of CO_2_ adducts 78 between AlX_3_ (X = Cl, Br) and PR_3_ (R = Mes) (Fig. 7). Upon treatment of these adducts with excess ammonia borane (NH_3_·BH_3_), an Al-methoxy species was generated which after hydrolysis resulted in the formation of MeOH at room temperature.^103^ To study the steps involved in the reaction, Me_3_N·BH_3_ was utilised to reduce the FLP-CO_2_ adduct 78 (X = C_6_F_5_, R = *o*-Tol). Along with the methoxy derivatives of alane, compound 79 was isolated and fully characterised.^114^ Later, other groups have studied the mechanism for this reaction computationally and explained the reduction of CO_2_ trapped FLPs.^115^ In other reactions, Stephan and co-workers explored the adducts of 78 (X = Cl, Br, I, C_6_F_5_, OC(CF_3_)_3_ and R = Mes, *o*-tolyl) for the stoichiometric transformation of CO_2_ to CO.^116^

**Scheme 50:**
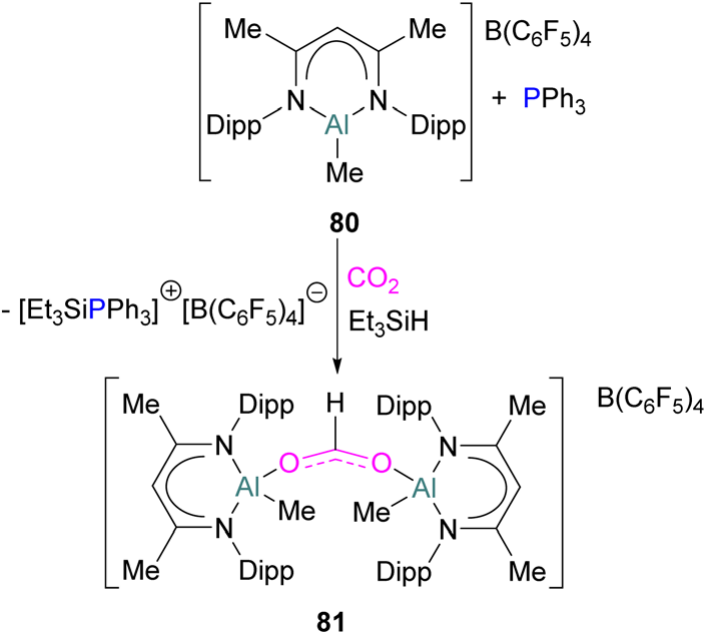
Aluminium based FLP for the stoichiometric reduction of CO_2_.

While fluorination of substituents is a common way to increase Lewis acidity in FLP design, an alternative is the use of a cationic Lewis acid. Harder reported an FLP comprised of Lewis basic PPh_3_ and a cationic [Dipp-NacNacAlMe]^+^ (NacNac = β-diketiminate ligand) Lewis acid 80.^117^ Exposure of this FLP to CO_2_ results in the rapid formation of a stable adduct which upon stoichiometric hydrosilylation with triethylsilane forms compound 81 (Scheme 50). Hydride transfer from Et_3_SiH to the carbon atom of CO_2_ generates Et_3_Si^+^ which is trapped by the base, PPh_3_, and forms an ion pair [Et_3_SiPPh_3_][B(C_6_F_5_)_4_]. The insertion of Et_3_Si^+^ into an ion pair restricts the system to stoichiometric CO_2_ reduction. Otherwise, cleavage of the Al–O bond and transfer of formate ion HCO_2_^−^ to Et_3_Si^+^ would have made the system catalytic.

**Scheme 51:**
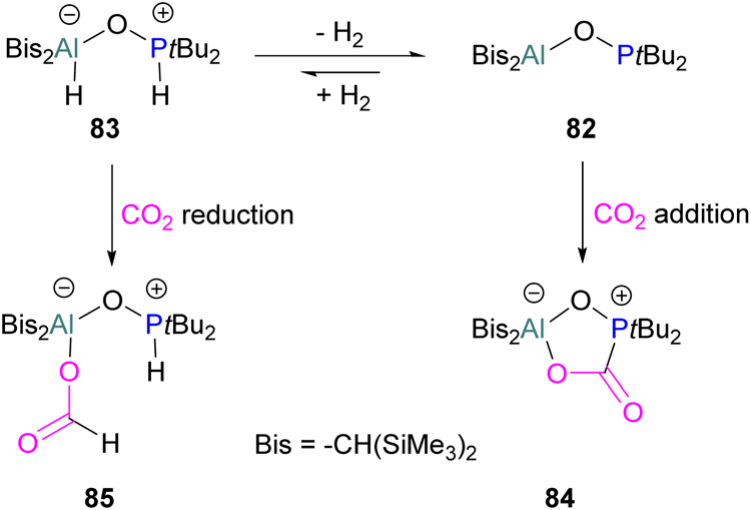
Oxygen-bridged geminal Al/P FLP for CO_2_ activation and reduction.

An unusual report of an oxygen-bridged geminal Al/P FLP 82 was made by Wickemeyer *et al.* (Scheme 51). It was prepared by reaction of the parent alane and phosphine oxide, giving an initial zwitterionic compound 83 which slowly eliminates H_2_ to give the FLP 82. 82 was found to bind CO_2_ to give the heterocyclic CO_2_ adduct 84. The hydrogenated zwitterion 83 can also be generated in small quantities by exposure of the FLP to H_2_. This species bound CO_2_ irreversibly, and exposure of the hydrogen adduct to CO_2_ gave stoichiometric CO_2_ reduction to the aluminium bound formate 85.

However, the instability of the hydrogen adduct and strong Al–O bond make the system not well suited for catalytic applications (Scheme 51).^118^

Huang *et al.* reported a variety of group 12 and group 13 formamidinate FLPs. While formamidinates are able to coordinate as bidentate ligands, the incorporation of strongly electron-withdrawing C_6_F_5_ substituents on nitrogen increases the preference of the monodentate species with a vacant coordination site on the metal. The free “N” and unsaturated metal in proximity are able to act as an FLP,^119^ and the compounds' potential for catalytic CO_2_ hydrosilylation. While the formamidinates investigated (B, Al, Ga, In and Zn) showed poor activity for this reaction on their own, significantly improved performance was seen when combined with B(C_6_F_5_)_3_ or Al(C_6_F_5_)_3_. The highest activity for complete conversion of triethylsilane under 1 bar CO_2_ after 10 h at 80 °C was observed with the aluminium formamidinate/B(C_6_F_5_)_3_, yielding almost exclusively CH_4_. Replacing Et_3_SiH with Ph_2_SiH_2_ gave selective formation of the bis(silyl ether).

However, mechanistic studies involving the aluminium formamidinate suggest that the catalyst decomposes under the reaction conditions to generate other aluminium species, which were the catalytically active species, and were not identified.

**Scheme 52:**
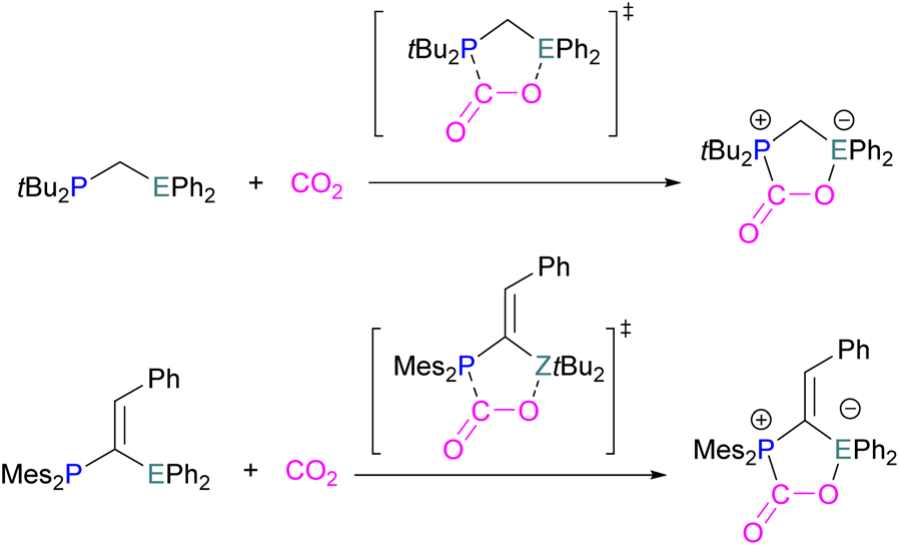
Geminal P/B and P/Al FLPs with CO_2_.

Surawatanawong and co-workers compared the reactivity of geminal P/Al and B/P FLPs with CO_2_ and H_2_ based on systems previously published by Lammertsma *et al.*^42^ The compounds investigated consisted of an sp^2^-carbon bridged FLP (Mes_2_P–C(=CHPh)–E*t*Bu_2_) and an sp^3^-carbon bridged FLP (*t*Bu_2_P-CH_2_-EPh_2_) (E = B, Al). In their comparative study between the geminal B/P and Al/P FLP activation of CO_2_ and H_2_ (Scheme 52), the main conclusions the authors drew are that the FLPs are more reactive towards CO_2_ than H_2_, and that the geminal B/P FLPs involve stronger orbital interactions with CO_2_ than their Al/P counterparts. Distortion–interaction decomposition showed that the distortion energy in the H_2_ fragment is higher than that in the CO_2_ transition state leading to a higher energy barrier for H_2_ activation than CO_2_ activation. This again highlights the importance of considering energy barriers to the activation of both CO_2_ and H_2_, similarly highlighted by the work of Corminboeuf above (Scheme 31). The type of geminal linker, sp^2^ or sp^3^, was found not to affect the reactivity.^120^

### Base-free CO_2_ reduction with group 13 Lewis acids

**Scheme 53:**
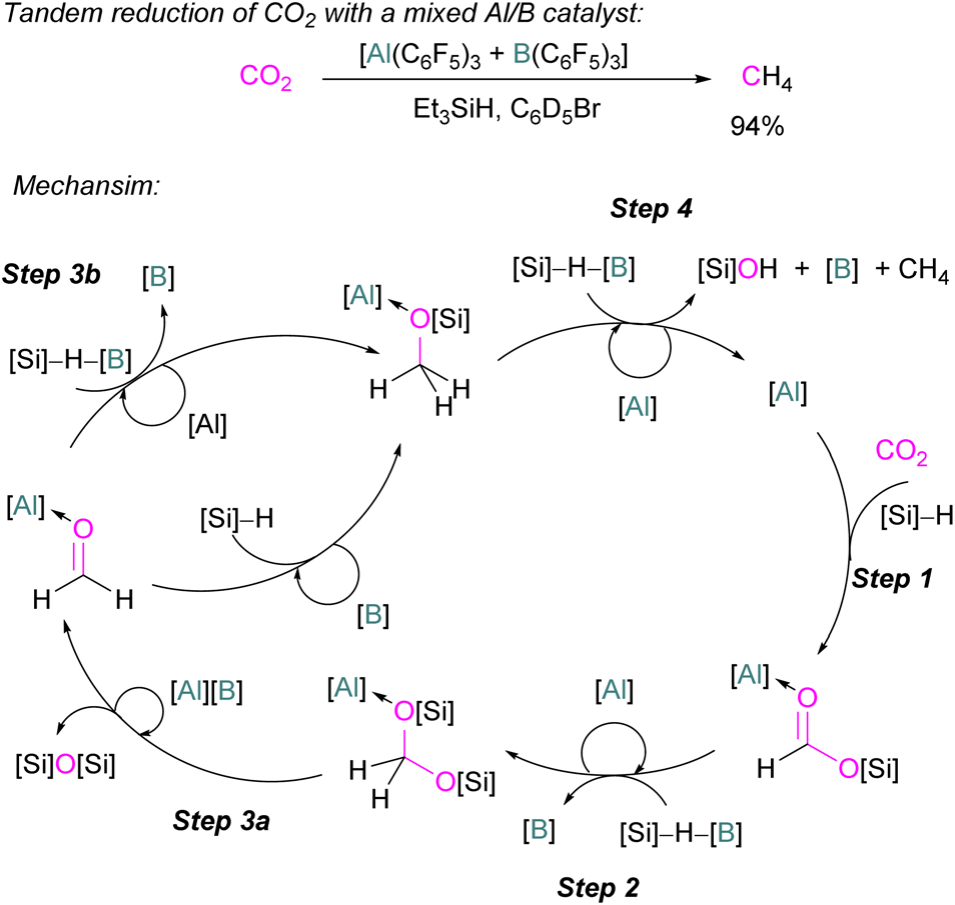
Catalytic reduction of CO_2_ by mixed Lewis acids [Al(C_6_F_5_)_3_ and B(C_6_F_5_)_3_]. [B] = B(C_6_F_5_)_3_; [Al] = Al(C_6_F_5_)_3_; [Si] = SiEt_3_.

In the final example using only Group 13 Lewis acids, without a base, Chen and co-workers reported the first example of a mixed Lewis acid system consisting of Al(C_6_F_5_)_3_ and B(C_6_F_5_)_3_ for the highly selective reduction of CO_2_ into CH_4_*via* a tandem hydrosilylation (Scheme 53). The reaction proceeds in a catalytic manner. In the first step, Al(C_6_F_5_)_3_ effectively mediates the overall hydrosilylation cycle fixing CO_2_ into HCO_2_SiEt_3_ by activating the carbonyl group. For this initial transformation B(C_6_F_5_)_3_ was found to be inefficient but for the subsequent reduction steps to CH_4_ (Scheme 53, steps 2–4) B(C_6_F_5_)_3_ was found to be crucial to give CH_4_ in up to 94% yield through a frustrated Lewis pair (FLP)-type Si–H activation. The higher Lewis acidity of Al(C_6_F_5_)_3_ relative to the corresponding borane led to the formation of stable intermediates ([Al]-substrate adducts and [Al]-intermediates). In this reaction for the overall reduction of CO_2_ to CH_4_, the role observed for both Lewis acids are not only complementary but also synergic where the first reduction step is initiated by the aluminium catalyst and later by the boron catalyst.^121^

### Gallium and indium FLPs for CO_2_ activation

**Scheme 54:**
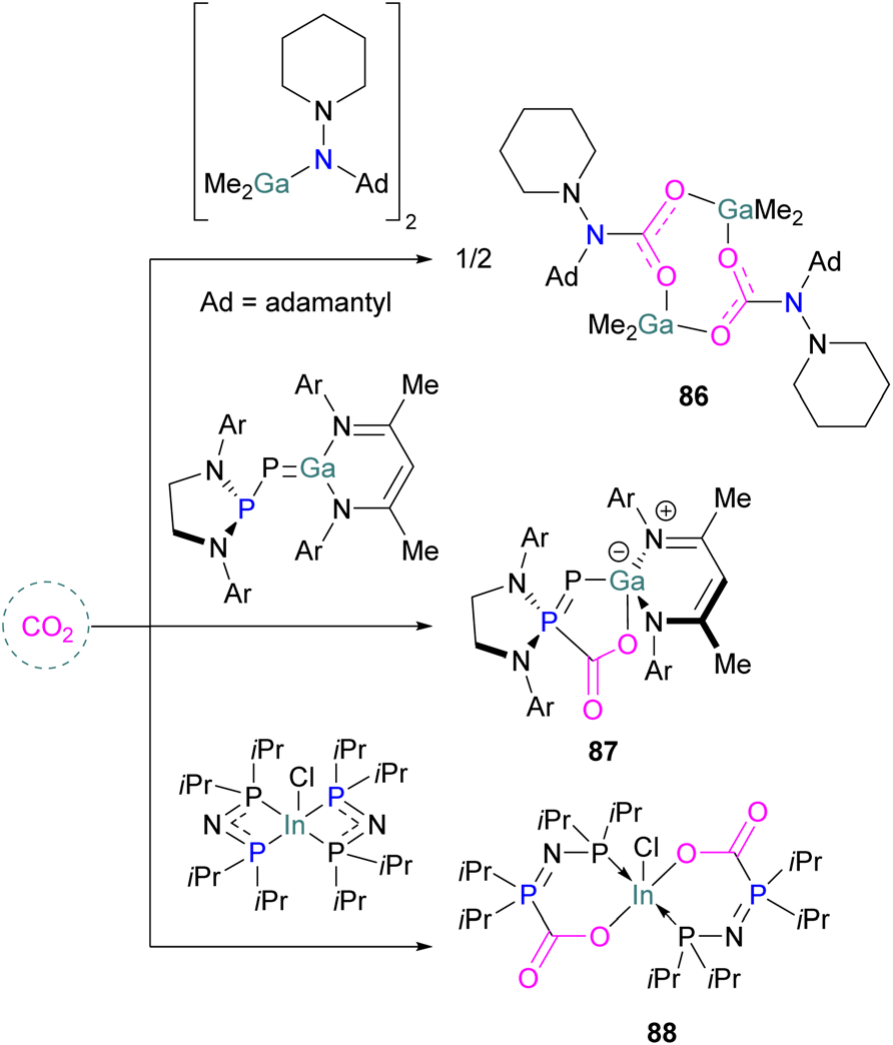
FLP type reactivity of homogenous indium and gallium systems with CO_2_.

Examples of homogenous gallium and indium containing FLPs in CO_2_ activation are rare although several heterogenous systems are known for indium (see later). Uhl and co-workers reported a dimeric gallium hydrazide displaying FLP-like reactivity able to insert CO_2_ into the Ga–N bond, yielding a seven-membered C_2_O_4_Ga_2_ cycle 86 (Scheme 54, top).^122^ An atypical example of FLP-like reactivity with CO_2_ was also reported by Goicoechea using a phosphanyl phosphagallene.

The compound adds to CO_2_ with oxidation of the phosphanyl phosphorus, with gallium bound to the phosphanyl phosphorus and one of the oxygen atoms bound to gallium 87 (Scheme 54, middle).^123^ Kemp and co-workers prepared a *P*,*P*-chelated heteroleptic complex bis[bis-(diisopropylphosphino)amido]indium chloride [(*i*Pr_2_P)_2_N]_2_InCl. In both the solid-state and solution, it was found that CO_2_ inserted into two of the four M–P bonds to produce [O_2_CP(*i*Pr_2_)NP(*i*Pr_2_)]_2_InCl 88 (Scheme 54, bottom). Experimental analysis showed that the time taken for the insertion of CO_2_ at room temperature in solution condition was less than 1 minute and less than 2 h in the solid–gas reaction. The complex was stable up to 60 °C under vacuum but released CO_2_ when heated above 75 °C.^124^

## Group 14 Lewis acids

Compared to group 13, group 14 elements have been less studied as Lewis acid components of FLPs for CO_2_ activation and conversion. Although carbenium ions such as trityl are isoelectronic with boron, the activation of CO_2_ with carbon Lewis acids within an FLP are not known to the best of our knowledge. Examples with heavier Group 14 Lewis acids are known, however, and are described below.

### Silicon FLPs for CO_2_ activation

Silicon cations are highly electrophilic and are therefore good candidates as the Lewis acid component of FLPs for small molecules activation. In addition, CO_2_ transformation into products such as benzoic acid, formic acid, and methanol using silicon cations formed from a [Ph_3_C][B(C_6_F_5_)_4_]/R_3_SiH system in different solvents has already been shown to be effective.^125^

**Fig. 8:**
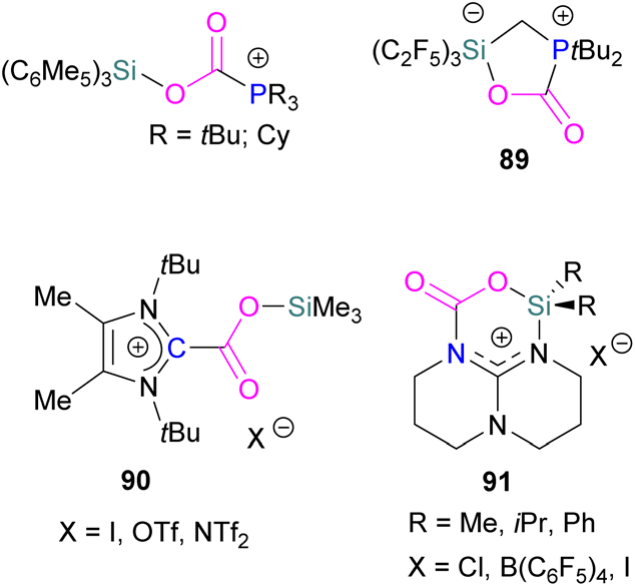
Si-based CO_2_ adducts.

With sterically hindered phosphines, triarylsilylium borates [Ar_3_Si^+^][B(C_6_F_5_)_4_] form FLPs. Müller and co-workers studied a series of silylium ion/phosphane Lewis pairs [Ar_3_Si^+^/PR_3_]. When Ar = Me_5_C_6_ and R = *t*Bu or Cy these FLPs were able to activate CO_2_ (1 atm, 30 min) in benzene at room temperature to obtain the FLP-CO_2_ adducts [R_3_P–CO_2_–SiAr_3_] (Fig. 8).^126^ In 2015, Mitzel and co-workers reported the first synthesis of a neutral Si/P FLP (C_2_F_5_)_3_SiCH_2_P*t*Bu_2_ and this was utilised in trapping CO_2_ at room temperature as a cyclic adduct 89 in quantitative yields.^127^

The stability of the adduct of a trimethylsilylium and a congested *N*-heterocyclic carbene (90) was found to be strongly dependent on the nature of the counterion used in the reaction. The stability was found to increase with decreasing nucleophilicity of the ion X, or increasing Lewis acidity of the silylating agent Me_3_SiX (X = I, OTf, NTf_2_; Tf = SO_2_CF_3_).^128^ Tamm and co-workers explored the *N*-heterocyclic carbene-silylium ion frustrated FLP for the synthesis of adduct 90 (Fig. 8).^129^

Like the N/B intramolecular FLP described earlier, Cantat and co-workers synthesised a series of TBDR_2_SiX [R = Me, *i*Pr, Ph; X = Cl, B(C_6_F_5_)_4_, I; TBD = triazabicyclodecene] compounds and utilised them for CO_2_ capture to obtain N/Si^+^ FLP-CO_2_ adducts 91 (Fig. 8). The formation and stability of the adducts are dependent on the steric and electronic environment at the silicon centre.

**Scheme 55:**
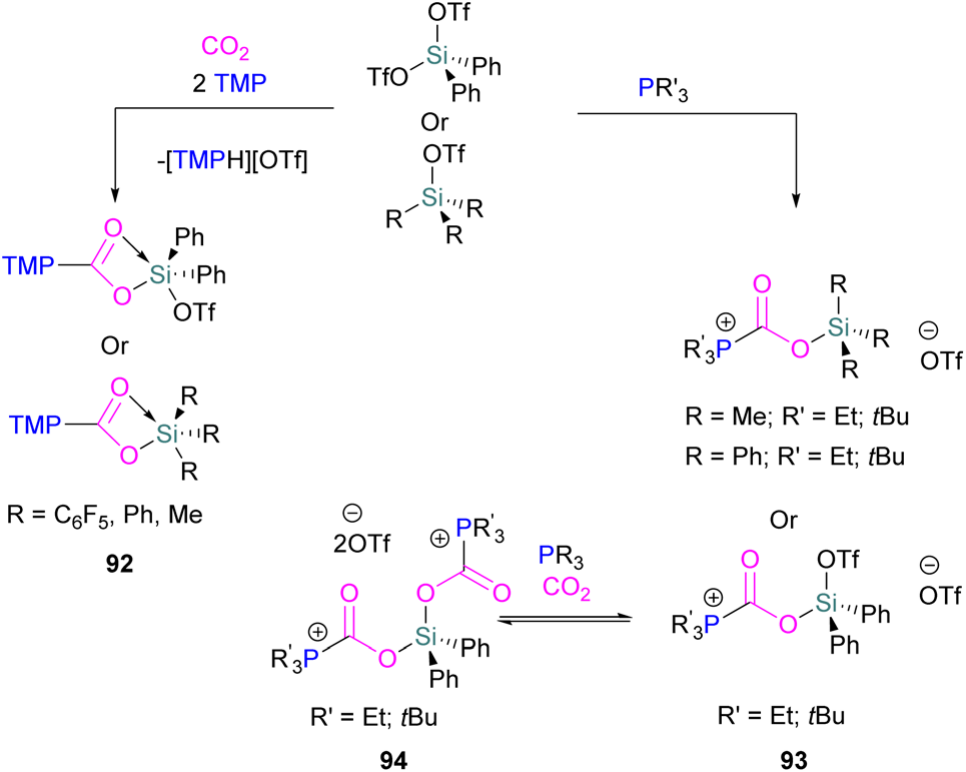
Si-based CO_2_ adducts using silyl triflates as Lewis acids.

Among the synthesised series, R = Me and X = Cl was found to be a good FLP adduct in reducing CO_2_ to methoxyboranes (R_2_BOMe) using 9-BBN as a reducing agent both in a stoichiometric and catalytic way. The authors carried out DFT calculations in support of their experimental results to explore the role of N/Si^+^ FLP-CO_2_ adducts in the catalytic reduction of CO_2_ with different boranes.^130^ They synthesised a series of *o*-phenylene-bridged phosphorus–silicon Lewis pairs and investigated their reactivity towards CO_2_ but no reaction was observed.^131^ Stephan and co-workers applied silyl triflates of the form R_4−*n*_Si(OTf)_*n*_ (R = C_6_F_5_, Ph, Me; *n* = 1, 2; OTf = OSO_2_CF_3_) to activate CO_2_ for adduct formation with bulky amines and phosphines (Scheme 55). Silyl triflates Ph_2_SiOTf_2_ and R_3_SiOTf (R = C_6_F_5_, Ph, Me) with TMP formed the silyl carbamates 92. Trialkylphosphines also activate CO_2_ in combination with the silyl triflate generating FLP-CO_2_ adducts 93, with the silyl triflates R_3_SiOTf showing reversible CO_2_ binding. The bis-CO_2_ adduct 94 was obtained at lower temperature (–40 °C) and using excess phosphine.^132^

### Germanium and tin FLPs for CO_2_ activation

It has been proven that the cleavage of FLP-CO_2_ adducts are quite difficult to great extent due to the strong hard–hard interaction of oxygen and the typical hard Lewis acids used in the FLP system according to the HSAB principle.^133^ Ge and Sn are softer elements and are less oxophilic, thus their use could provide beneficial to enable catalytic CO_2_ reduction. Although FLPs with a germanium Lewis acidic centre are known and have been shown to activate small molecules, their use in CO_2_ activation and conversion is not yet reported.^134^

Mitzel and co-workers reported in 2019 the synthesis of a geminal Sn/P FLP (F_5_C_2_)_3_SnCH_2_P*t*Bu_2_ by reacting LiCH_2_P*t*Bu_2_ with (F_5_C_2_)_3_SnCl. When the FLP (F_5_C_2_)_3_SnCH_2_P*t*Bu_2_ was exposed to CO_2_ at −70 °C, it formed an adduct that was found to be reversible at 25 °C.^135^ Fernandez performed a theoretical analysis of the FLP systems (F_5_C_2_)_3_E–CH_2_–P*t*Bu_2_ (E = Si, Ge, Sn) to understand the effect of the nature of these group 14 elements on their reactivity. Moving down the group, the reactivity of these species is kinetically enhanced (Si < Ge < Sn). Quantitatively, this trend of reactivity was analysed by the “activation strain model” of reactivity in combination with the energy decomposition analysis method. A five-membered TS with CO_2_ lead to the experimentally observed zwitterionic products. The model identifies the interaction energy between the deformed reactants as the main factor controlling the reactivity of these geminal FLPs containing Si/Ge/Sn, where the lone pair of phosphorus donates into the π* orbital of C=O and a stronger electrostatic and orbital interaction is observed for Sn over Si.^136^ Similarly, Pati and co-workers computationally explored the ability of the FLP system (F_5_C_2_)_3_E–CH_2_–D(*t*Bu)_2_ where E = Si, Ge, Sn and D = P, N to act as hydrogenation catalysts using CO_2_ as a substrate.^137^ For the FLPs where D = N, simultaneous proton and hydride migration take place, whereas for D = P FLPs, proton transfer is followed by hydride transfer. NBO analysis shows that LP(O) → σ* (D–H) and σ(E–H) → π* (C=O) dominate along the energy profile. From their studies, they predict that FLP (C_2_F_5_)_3_Sn–CH_2_–N(*t*Bu)_2_ would be able to perform CO_2_ hydrogenation particularly well.

Hulla reported an application of tin-based FLPs in the form R_3_SnX/N-base (R = alkyl and X = OTf^−^ or NTf_2_^−^] which can catalyse the formation of azoles from *ortho*-substituted anilines *via* complete deoxygenation of CO_2_ in the presence of H_2_.^138^

### Computational insights into group 13 and 14 Lewis acids in FLP catalysed CO_2_ activation

**Fig. 9:**
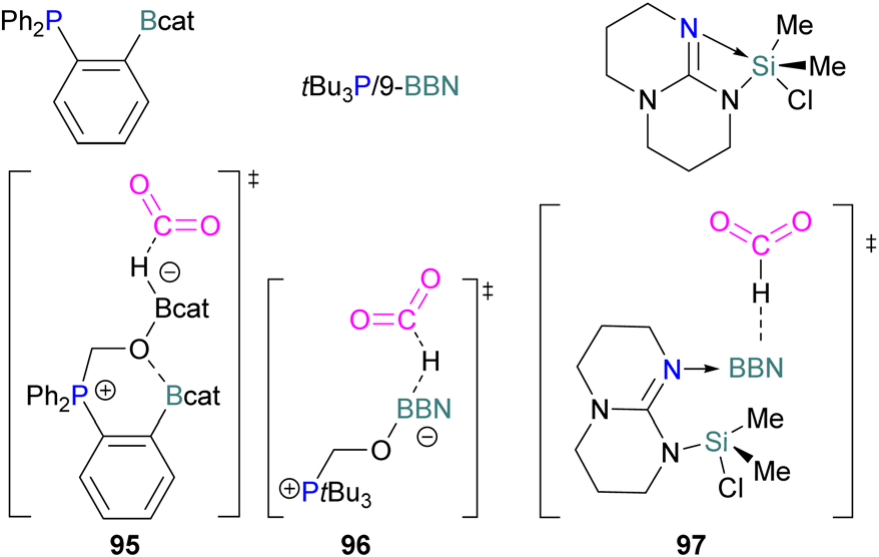
Substrates and intermediates involved in the FLP activation of CO_2_.

Grimme reported mechanistic insights, based on extensive DFT calculations, on all steps of the FLP catalysed reduction of CO_2_ to boryl formate, H_2_CO, bis(boryl) acetal, and methoxyl borane products in 2020.^139^ The work addressed three FLP catalysts that had been previously reported; (i) Fontaine's B/P intramolecular FLP reported in 2013,^55,56^ (ii) Stephan's intermolecular FLP consisting of *t*Bu_3_P and 9-BBN from 2014,^58^ and (iii) Cantat's 2016 Si/N FLP with 9-BBN (Fig. 9, top).^130^ The report unveils the importance of the Lewis-basic CH_2_O “oxide” site in promoting a hydride transfer, from calculations (PW6B95-D3+COSMO-RS//TPSS–D3+COSMO level of theory in THF). Initial formation of a zwitterionic FLP-H_2_CO adduct had been proposed previously and was verified in this report, in the intramolecular FLP reported by Fontaine, this is generated through the Lewis-basic Bcat oxygen atoms 95 (Fig. 9).

Subsequent hydride transfer from the FLP-H_2_CO adduct to CO_2_ then forms boryl formate HCOOBcat through a series of steps, identifying the Lewis acidic Bcat group as the ‘base shuttle’. For Stephan's intermolecular *t*Bu_3_P/9-BBN FLP, a hydride transfer from *t*Bu_3_P–CH_2_O–9-BBN to CO_2_*via*96 (Fig. 9) is exergonic by −16.1 kcal mol^−1^ with a barrier of 7.2 kcal mol^−1^, which is feasible at room temperature. The final reduction step from H_2_C(O-9-BBN)_2_ into H_3_CO-9-BBN is the slowest reduction step, with a barrier of 23.3 kcal mol^−1^. Lastly, when investigating the mechanism for Cantat's Si/N FLP, Grimme and co-workers found the neutral adduct between the Lewis-basic N and 9-BBN to be the most energetically favourable starting point. Here, the B–H is partially activated by the Si/N centres. Hydride transfer to CO_2_ is then exergonic by −7.5 kcal mol^−1^*via*97 (Fig. 9). In summary, zwitterionic FLP-H_2_CO adducts were found to be the active catalysts, strong oxygen and nitrogen Lewis bases were found to stabilise the hydride transfer steps to CO_2_, and finally, Lewis-acidic groups such as Bcat were found to act as a base shuttle.

## Group 15 Lewis acids

**Fig. 10:**
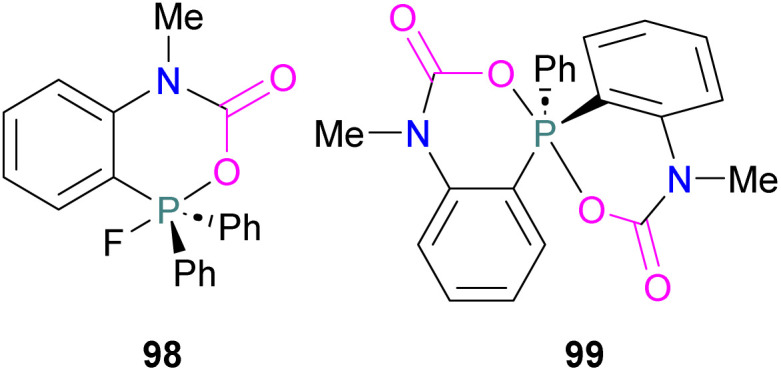
Phosphorus as a Lewis acid in CO_2_ capture.

Generally, in FLP chemistry, group 15 elements are employed as the Lewis base component due to the presence of a lone pair when in the +3 oxidation state. Although nitrogen Lewis acids are known in FLPs, and have been used for small molecule activation, their application for CO_2_ activation has not been explored.^140^ On the other hand, there are a few examples using phosphorus as the Lewis acid. An example was reported by Stephan who prepared a CO_2_-adduct 98 based on intramolecular amidophosphoranes where the phosphorus acts as a Lewis acidic centre and a nitrogen centre in the parent FLP acts as a nucleophilic centre to capture CO_2_ (1 atm) at ambient temperatures. Similarly, the bis-CO_2_-adduct 99 (Fig. 10) was also prepared under the same reaction conditions.^141^ A detailed computational mechanism was studied for the adduct 98 by Zhu and co-workers. They investigated that ring strain, and the *trans-*influence are the key factors in amidophosphoranes to capture CO_2_.^142^

## Transition metal Lewis acids

**Scheme 56:**
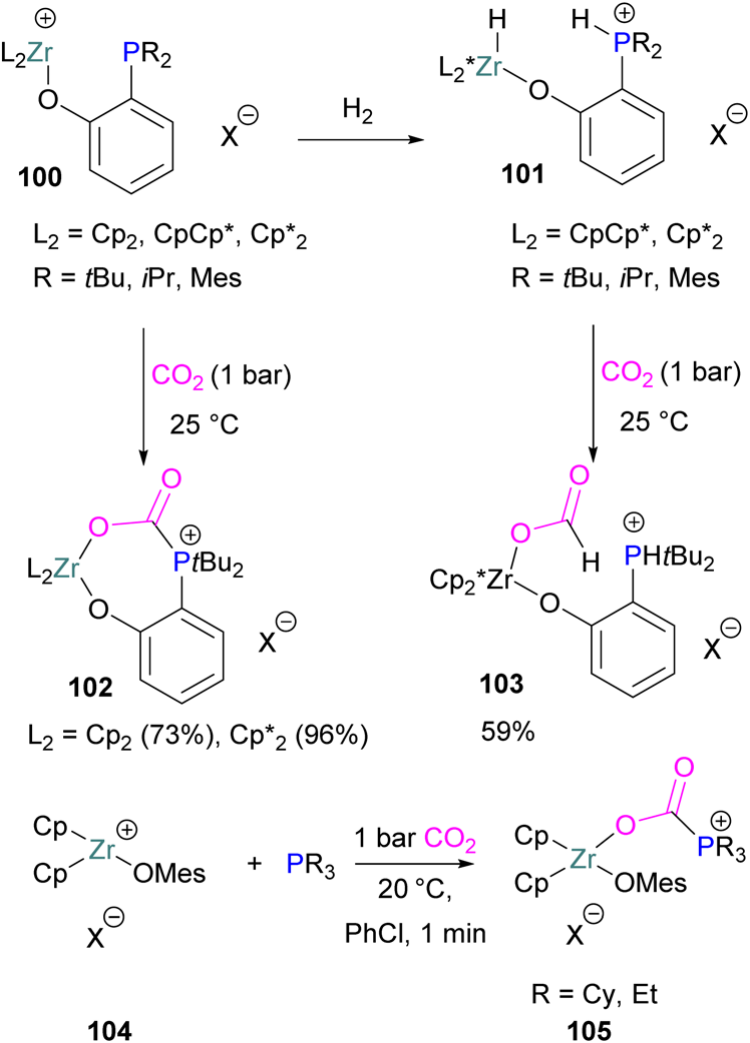
Reaction of zirconium FLPs with H_2_ and with CO_2_. X^−^ = [B(C_6_F_5_)_3_]^−^, Cp = cyclopentadienyl, C_6_H_5_; Cp* = pentamethylcyclopentadienyl, C_5_Me_5_.

Earlier we have discussed examples of how low valent transition metals can act as the Lewis base of an FLP when combined with boron Lewis acids. In this section we will discuss selected reports where the transition metal behaves as the Lewis acid to activate CO_2_ in an FLP fashion. Several early examples by Piers reported the use of Lewis acidic scandium complexes in combination with B(C_6_F_5_)_3_ and a silane to be operative under an FLP type mechanism to reduce CO_2_.^143,144^ Examples by Wass and co-workers in 2011, however, were the first to extend the concept of FLPs to transition metals through the use of cationic zirconocene–phosphinoaryloxide complexes.^145^ Wass reported the synthesis of zirconocene–phosphinoaryloxide complexes 100 and their applications in the FLP activation of H_2_ to generate 101 and activation of CO_2_ to give the FLP-CO_2_ adduct 102. 102 showed no further reaction with H_2_, however 101 could insert into CO_2_ under mild conditions to generate 103 (Scheme 56, top). A similar system also reported by Wass focuses on intermolecular zirconium/phosphorus FLPs where a zirconium(iv) cation 104 is combined with a tertiary phosphine. Activation of CO_2_ occurred under mild conditions to yield the adduct 105 (Scheme 56, bottom).^146^

**Scheme 57:**
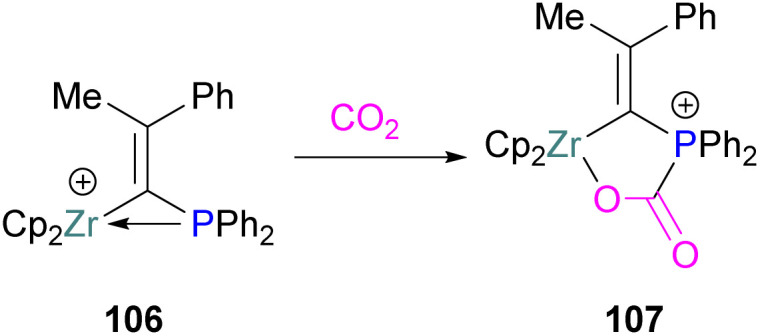
Zr^+^/P Pair system in activation of CO_2_.

Systematic modification of the phosphine Lewis base showed that FLPs with modest Tolman steric parameters are highly reactive and have the maximum selectivity for the intended product. The base was found to affect the selectivity, and PEt_3_ gave the cleanest results. These later findings demonstrate that transition metal FLPs do not require intramolecular systems and allow for the construction of intermolecular transition metal frustrated or cooperative Lewis pairs. Another zirconium based FLP has been reported by Erker in the form of an intramolecular cationic geminal Zr^+^/P pair 106 which could react with CO_2_ to form a five-membered metallaheterocyclic adduct 107 (Scheme 57).^147^ Systems based on 106 have been the subject of theoretical studies for the reactivity of the Zr^+^/P pair system in the activation of CO_2_.^148^ Whereas, computational investigations reveal that the activation reaction between CO_2_ and Zr^+^/P-based FLP-associated compounds is exothermic and coherent, generating a cyclic ring. The Zr^+^–P bond length contributes to the reactivity of these compounds. The donor–acceptor relationship was also found to determine the bonding nature of the activation reactions between CO_2_ and Zr^+^/P-based FLP-related compounds. Accordingly, the calculated O=C=O bond stretching distance and O=C=O bending angle relate to the activation energy for CO_2_ activation reactions with Zr^+^/P-based FLP-related compounds, in line with Hammond's postulate.

The heavier group 4 metal hafnium has also been shown to undergo FLP-type CO_2_ activation between the metal centre and a pendant Lewis basic centre on the ligand.

**Scheme 58:**
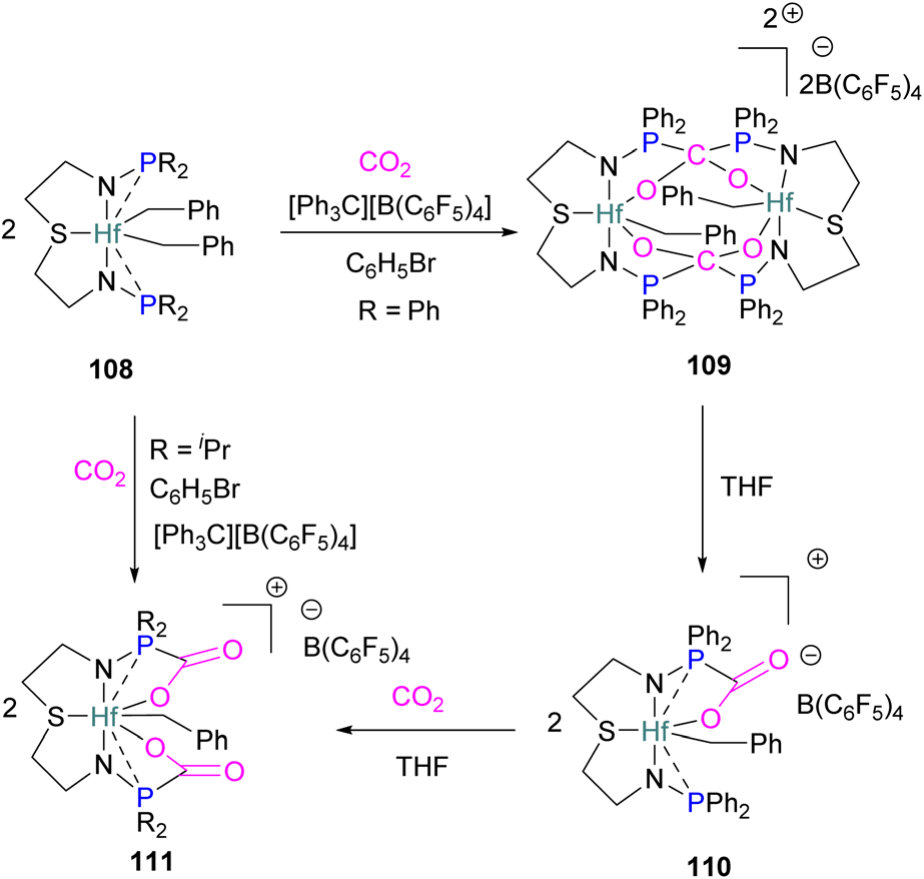
CO_2_ adduct formation using an intramolecular hafnium FLP.

**Scheme 59:**
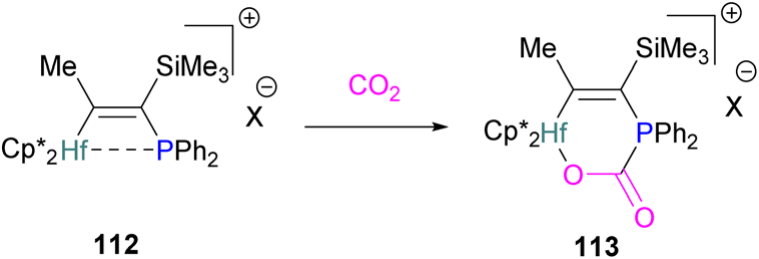
Vicinal hafnium FLP for CO_2_ activation. X^−^ = [B(C_6_F_5_)_4_]^−^.

The hafninum complex 108 was found to react with one or two equivalents of CO_2_ to give a series of monometallic and bimetallic CO_2_ activated products (109–111) depending upon the substituents on the phosphine ligand (Scheme 58).^149^ In these complexes, the phosphinoamines binds to hafnium *via* the nitrogen atom, and binds weakly through the softer phosphorus atom. Reaction of metallocene cation complexes [Cp*_2_HfMe][B(C_6_F_5_)_4_] with trimethylsilyl-(diarylphosphino)acetylenes yielded internal phosphane stabilised hafnium cations [Cp*_2_Hf–C(Me)–C(SiMe_3_)PPh_2_][B(C_6_F_5_)_4_]. As with other vicinal compounds, Hf^+^/P 112 exhibits FLP-like reactivity and generates the adduct 113 when reacted with CO_2_ (Scheme 59).^150^

**Scheme 60:**
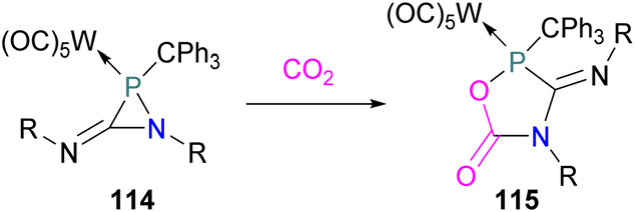
Tungsten-accelerated CO_2_ activation.

An alternative approach is to use a transition metal to assist CO_2_ activation as demonstrated by Streubel and co-workers. The 3-imino-azaphosphiridine complex 114 was prepared and reacted with CO_2_ to obtain a heterocyclic compound 115 (Scheme 60).^151^

**Scheme 61:**
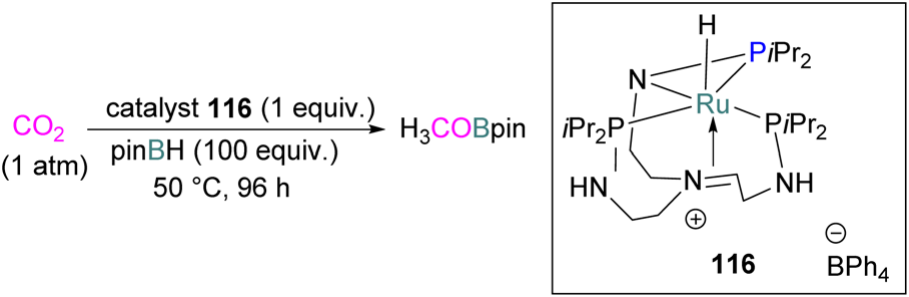
Catalytic reduction of CO_2_ using a Ru–H salt and pinBH.

While numerous creative strategies are being developed for CO_2_ capture, there is an increasing interest in using carbon dioxide as a C1 carbon source. The hydrogenation of CO_2_ to formic acid and its derivatives is one such well-developed strategy based on Ru.^152^ In 2012, Stephan and co-workers developed an elegant catalytic system based on ruthenium hydride 116 (Scheme 61). This salt was explored in the reduction of CO_2_ catalytically using HBpin as a reducing agent. One equivalent of 116 and 18 equivalents of HBpin under an atmosphere of CO_2_ gave the MeOBPin product catalytically after 96 h at 50 °C. Increasing the ratio of 116 : HBpin to 1 : 100 resulted in a small increase in the TON.^153^ In this system the RuNP ring in the catalyst is similar to FLP systems, and the binding of CO_2_ depends upon the cooperative action of the Lewis acidic metal centre with one of the Lewis basic phosphine centres in the ligand. Cleavage of the C–P bond upon reduction with HBpin and a transfer of oxygen from Ru to Bpin allows the system for a catalytic hydroboration of CO_2_. A similar cooperativity between metal and ligand was observed by Crispin and co-workers^154^ who reported the synthesis of iridium–pyridylidene complexes [Tp^Me2^Ir(C_6_H_5_)_2_(C(CH)_3_C(R)NH] (Tp^Me2^ = hydrotris(3,5-dimethylpyrazolyl)borate; R = H, Me, Ph) 117.

**Scheme 62:**
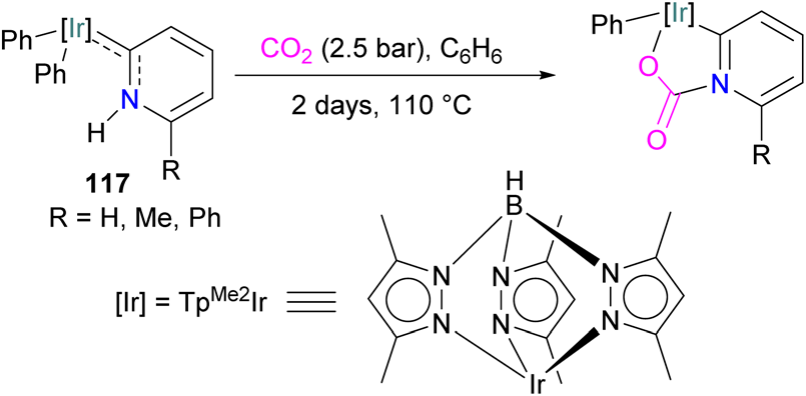
Cooperative activation of CO_2_ using an iridium catalyst.

These species can activate a range of small molecules including CO_2_ (R=H) in an FLP fashion between a Lewis acidic iridium centre and a Lewis basic nitrogen atom on the pyridyl ligand (Scheme 62).

**Scheme 63:**
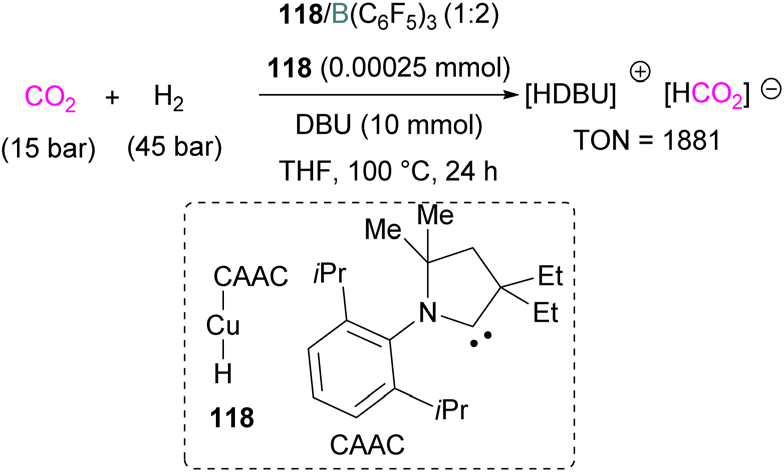
Catalytic reduction of CO_2_ with Cu–H/Lewis pair system.

Similar to the tungsten system described earlier, other metals have been employed to assist classical FLPs in CO_2_ reduction. A copper-hydride system has been developed by Bertrand and co-workers for the activation and reduction of CO_2_ in synergy with a N/B FLP. Amongst several screened reactions, both stoichiometric as well as catalytic, the authors found that a catalytic system consisting of a (CAAC)CuH (CAAC = cyclic (alkyl)(amino)carbene) 118 with B(C_6_F_5_)_3_ in a 1 : 2 ratio and DBU (10 mmol) forms the formate salts of DBU from CO_2_ (15 bar) and H_2_ (45 bar) when heating the reaction mixture at 100 °C for 24 h in THF (Scheme 63). The key step in this reaction is the insertion of CO_2_ into the copper hydride and regeneration of copper hydride with H_2_. While the Cu–H bond readily inserts CO_2_, it is difficult for copper to activate H_2_. Thus, the FLP assists by activating H_2_ to allow regeneration of the copper hydride. The TON for this reaction is observed as 1881.^155^

Zinc metal has been explored for the reduction of CO_2_ to CO. Stephan reported the *in situ* formation of the catalytically active species Et_3_P=C=PEt_3_ through the reduction of CO_2_ to CO employing CH_2_I_2_.

**Scheme 64:**
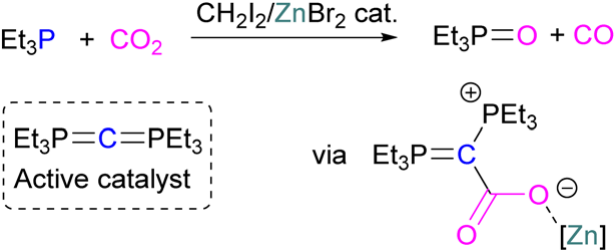
Reduction of CO_2_ using phosphaketene/Zn FLPs.

This (bis)ylide was found to interact with CO_2_, eliminating the phosphine oxide Et_3_PO as a by-product and forming an interim phosphaketene. The addition of catalytic ZnBr_2_ was found to facilitate the process through an FLP-type activation mode and was important for the regeneration of the (bis)ylide with simultaneous removal of CO (Scheme 64).^156^ The same authors described Zn-based FLP chemistry for functionalising CO_2_, using *t*Bu_3_P/ZnEt_2_ FLPs.^157^

The transition metal based Lewis acids in the activation of CO_2_ benefit from a higher coordination number when compared to the lighter main-group Lewis acids that were discussed previously. The ability of the TM species, such as in 110 and 116, to bind CO_2_ as well as ligand systems in more intricate intermediary species offers a higher degree of fine-tuning of the stability of adducts formed. Hence, higher turnover numbers may be observed for transition metal systems, as regeneration of the active catalyst species is kinetically more favourable. This may also explain for the more accessible use of H_2_ as the reducing source compared to silanes or hydrogen surrogates often used for the main group systems. This is seen for 118 and 100, whereas main group based Lewis acids frequently require pre-organised reducing sources, limiting the reaction to hydroboration or hydrosilylation of CO_2_ rather than direct hydrogenation. Nonetheless, concerns of toxicity and environmental impact drive an increased interest in transition metal free reagents. The work on main-group CO_2_ activation has focused on mimicking transition metals in synergistically accepting and donating electrons in the activation of CO_2_ with species that combine filled and vacant orbitals.

## Rare earth metals

**Scheme 65:**
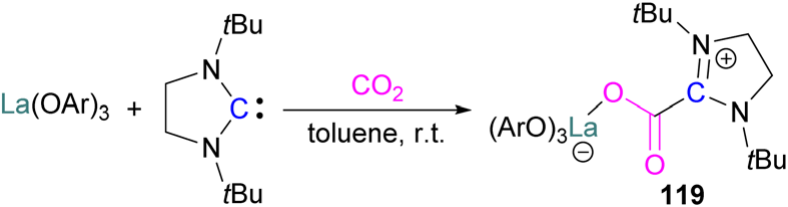
La-in CO_2_ activation. Ar = 2,6-*t*Bu_2_C_6_H_3_.

Rare earth metals are often employed as Lewis acids for a variety of reactions, and have also been employed as Lewis acids in FLPs. Dihydrogen was readily activated by combination of homoleptic rare-earth metal aryloxides, RE(OAr)_3_ (RE = La, Sm, and Y) with *N*-heterocyclic carbenes (NHCs) under mild conditions. In addition, the La/NHC pair exhibited FLP-like reactivity towards carbon dioxide, affording 1,2-addition products 119, as shown in Scheme 65.^158^

## Heterogenous FLPs

**Scheme 66:**
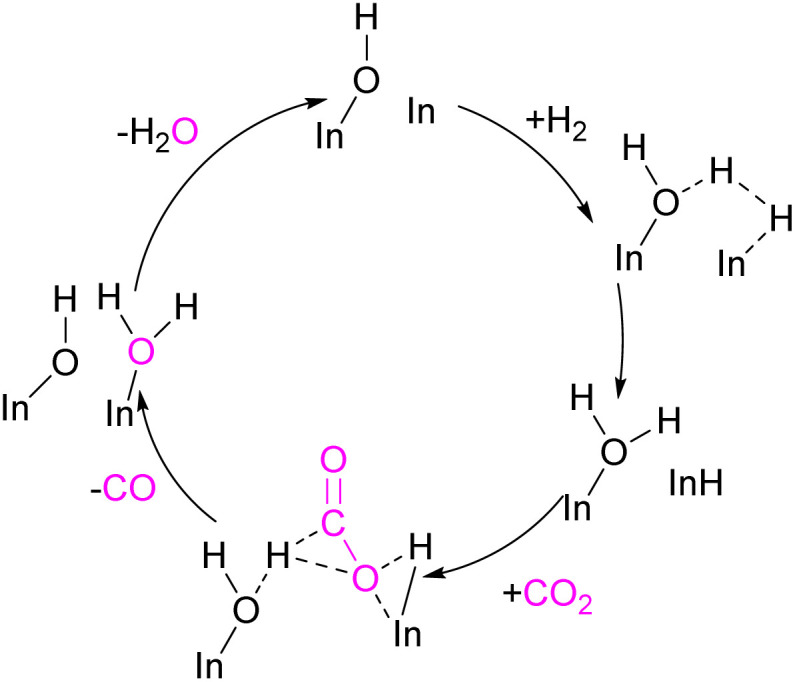
In-FLP mediated CO_2_ reduction.

Recent discoveries of FLPs as homogeneous catalysts has more recently turned to heterogeneous catalysts which operate by an FLP-type mechanism in which the Lewis acidic and Lewis basic centres in the solid structure activate CO_2_. Here, the understanding the chemistry of reactants, intermediates, and products on surfaces is crucial for designing catalytic nanostructures that transform carbon dioxide into carbon-based fuels. Several systems have been reported using indium as a Lewis acid. For example, indium oxide nanocrystals, In_2_O_3*x*_(OH)_*y*_, can catalyse the reverse water gas shift reaction, reducing carbon dioxide to carbon monoxide and water.^159^ Surface hydroxide groups and oxygen vacancies facilitate this reaction as shown in Scheme 66.

The enhancement of activity in the gas-phase reverse water gas shift process has also been investigated, as well as the distinct photoactive behaviour of pristine and defective indium oxide surfaces.^160^ Based on TD-DFT calculations, this study discovered that surface FLP in In_2_O_3*x*_(OH)_*y*_ has Lewis acidic indium sites close to a Lewis basic surface hydroxide. These acquired more acidity/basicity making them more active in the excited state relative to the ground state. In the photochemical reaction this reduces the activation energy relative to the thermal reaction, and could provide a mechanism to design improved photocatalytic systems for solar fuel production. In 2018, Ozin and co-workers, reported a similar system based on a rod-like nanocrystal superstructure of In_2_O_3−*x*_(OH)_*y*_, that could effectively catalyse the hydrogenation of CO_2_ to methanol under light at atmospheric pressure. The rate of conversion was found to be 0.06 mmol g^−1^ h^−1^ with a 50% selectivity and a long-term working stability.^161^

**Fig. 11:**
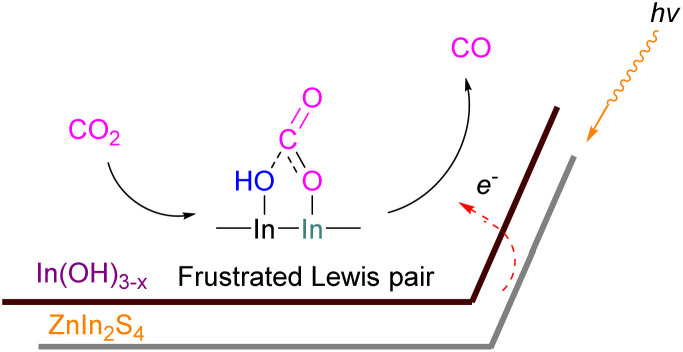
An FLP ZnIn_2_S_4_/In(OH)_3 − *x*_ (ZIOS) heterojunction system for the reduction of CO_2_ to CO.

Another heterogenous system based on indium has been developed by Wang and co-workers (Fig. 11). The authors developed a photocatalytic material based on a ZnIn_2_S_4_/In(OH)_3 − *x*_ heterojunction that works in a cooperative fashion to reduce CO_2_ into CO driven by light. The ZnIn_2_S_4_ functions to harvest the light and transport an electron to the FLP-activated CO_2_ on the In(OH)_3−*x*_ surface. In In(OH)_3−*x*_, the hydroxyl-deficient vacancies (OH_Vs_) acts as a Lewis acid, and the adjacent hydroxyl groups act as a Lewis base generating the FLP which activates CO_2_. This composite showed a CO formation rate of 1945.5 μmol g^−1^ h^−1^.^162^

Above we have seen that heterogenous FLPs can capture and react with H_2_ and CO_2_, boosting photocatalytic CO_2_ reduction. Isomorphous substitution of In^3+^ with Bi^3+^ has been found to increase catalytically active surface FLPs. Isomorphous substitution optimises surface catalytic active sites and affects optoelectronic characteristics, improving our understanding of photocatalytic CO_2_ reduction. Such isomorphous substitution will help to develop CO_2_ reduction materials with higher catalytic performance by tuning surface FLP site strength.^163^ Another bismuth containing heterogenous system has been reported by Wang who synthesised Sn-doped BiOBr with oxygen vacancies. The synthesised material possesses surface frustrated Lewis acidic (bismuth) and Lewis basic (lattice oxygen) pairs in BiOBr through the substitution of Bi^3+^ with Sn^4+^. 4Sn–BiOBr showed the best performance for photocatalytic CO_2_ reduction into CO with a yield of 165.6 μmol g^−1^ h^−1^.^164^ Another main group heterogenous system also reported is B_3_P_3_ doped hexa-cata-hexabenzocoronene, a model of nanographene (B_3_P_3_·NG) which reacted with carbon dioxide. This multi FLP device binds three CO_2_ molecules sequentially or simultaneously on the B_3_P_3_·NG surface.^165^ For the CO_2_ reduction *via* dissociative chemisorption of H_2_, nanocarbon-based FLP bifunctional catalysts are becoming promising due to their unquenched electron transport capability. One study proposes a nanocarbon-based FLP catalyst for the CO_2_ reduction *via* the dissociative chemisorption of H_2_.^166^

The catalyst consists of nitrogen/phosphorus doped graphene and M(C_6_F_5_)_3_ (M = B, Al, Ga, In) as Lewis acids. The study demonstrates the potential of doped carbon-based FLPs as innovative nanostructure catalysts for CO_2_ reduction *via* molecular hydrogen. N-doped FLP catalysts with activation barriers between 0.01 and 0.11 eV are promising for CO_2_ reduction, potentially enabling CO_2_ reduction catalytic material design.

**Scheme 67:**
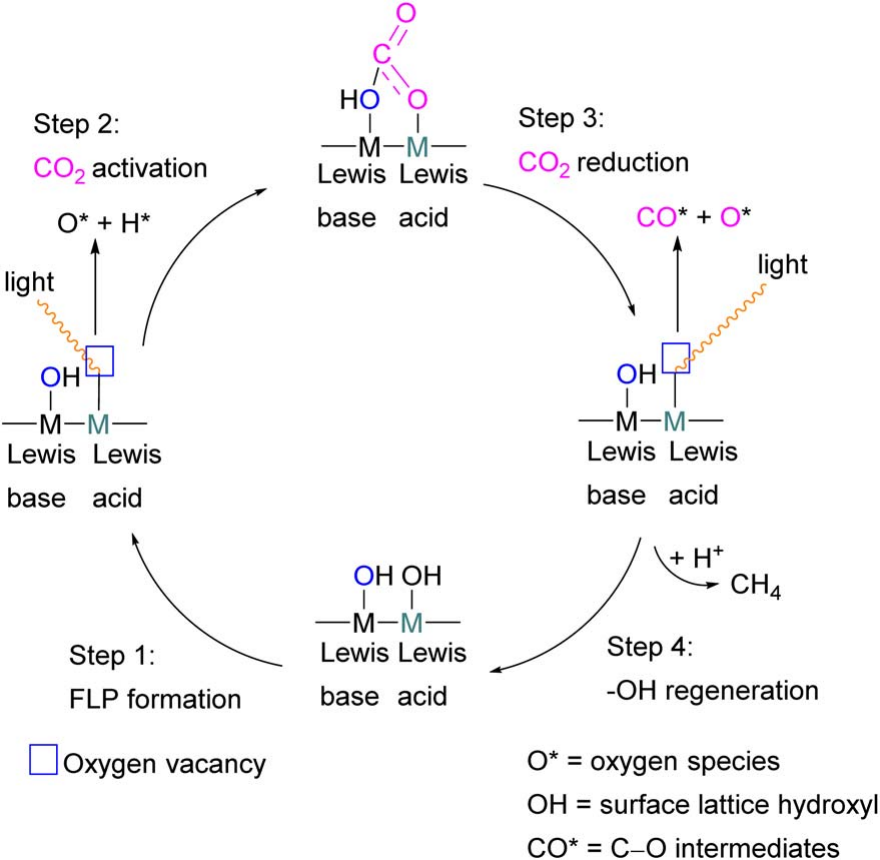
Heterogenous Ge FLP in the reduction of CO_2_ to CH_4_. M = Metallic element.

Converting and storing solar energy through light-driven CO_2_ reduction is a promising area of research. A particular approach for converting CO_2_ to methane gas uses hydroxyls inherent on an oxyhydroxide photocatalyst, such as in CoGeO_2_(OH)_2_, as a proton source. Irradiation of CoGeO_2_(OH)_2_ causes the lattice hydroxyls to be oxidised by photogenerated holes, leading to the formation of oxygen vacancies (OVs) and protons. These OVs and Lewis acid-base pairs bind CO_2_ and protons to activate it before reducing it to CH_4_. In the presence of water molecules, the surface lattice hydroxyls regenerate, allowing for continuous CO_2_ conversion as shown in Scheme 67. This strategy has the potential to pave the way for a novel use of photocatalysis in the field of energy conversion.^167^

**Fig. 12:**
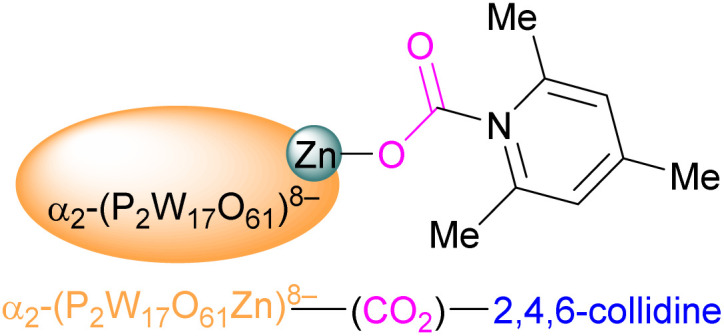
CO_2_ coordinated with α_2_-(P_2_W_17_O_61_Zn)^8−^ and 2,4,6-collidine.

Zinc based heterogenous systems have also been reported. Neumann and co-workers studied the coordination of CO_2_ to a Zn(ii) Lewis acid site in Wells–Dawson type polyoxometalates α_2_-(P_2_W_17_O_61_Zn)^8−^ which bound CO_2_ in an FLP fashion (Fig. 12). This system reveals two distinct binding modes: stronger “side-on” binding at higher temperatures and weaker “end-on” architectures at lower temperatures.^168^ This interaction with 2,4,6-collidine is possible through the development of a frustrated Lewis pair at lower temperatures.

**Fig. 13:**
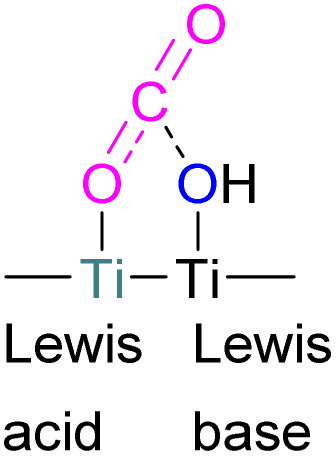
Photocatalytic reduction of CO_2_ with FLP-TiO_2−*x*_.

Like the indium oxide systems described above, efficient photocatalysts for CO_2_ reduction have also been reported using titanium. Anatase TiO_2−*x*_ hierarchical hollow boxes with FLPs can be synthesised through *in situ* topological modification of perovskite as shown in Fig. 13. These structures possess strong adsorption and activation properties, converting CO_2_ to CO without auxiliary substances. This innovative approach converts solar energy into chemical energy.^169^

The above examples of heterogeneous FLPs are important for the activation of CO_2_ compared to synthesising FLP-CO_2_ adducts.

There are some challenges to select suitable methods for achieving light absorption, electron–hole separation, energy gap matching for the reduction of CO_2_ to different products (product selectivity) in a photochemical way. The examples In_2_O_3*x*_(OH)_*y*_, ZnIn_2_S_4_/In(OH)_3−*x*_, 4Sn–BiOBr, and CoGeO_2_(OH)_2_ are promising systems for the photochemical reduction of CO_2_. The use of clean sources of the reducing agent H_2_ in these systems will suppress the chemical waste which is generated when activated reducing agents for the reduction of CO_2_ adducts are used.

**Scheme 68:**
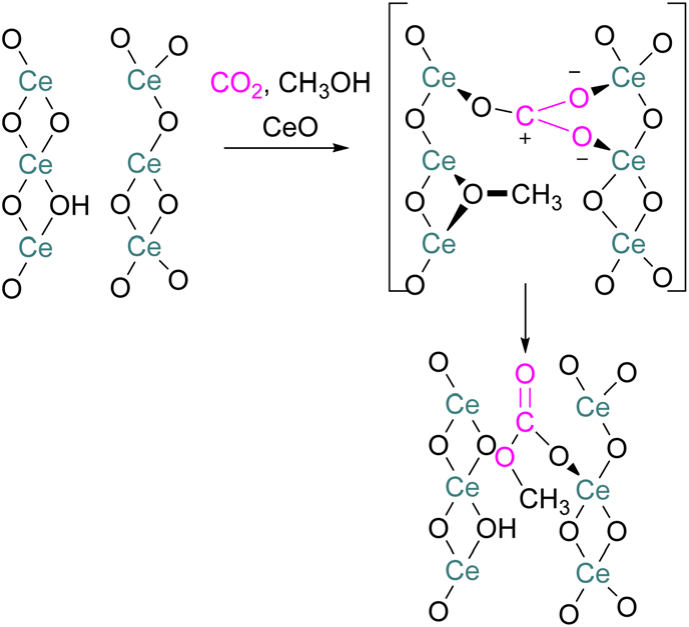
CO_2_ reduction using a CeO_2_ surface FLP to monomethylcarbonate.

Several reports of cerium as a Lewis acid in heterogenous FLPs are reported. Qu synthesised a defect-enriched cerium oxides (CeO_2_) with constructed interfacial FLPs (Ce^3+^···O^2−^) that activate CO_2_ efficiently *via* the interactions between the carbon atom of the CO_2_ molecule with the Lewis basic lattice O^2−^ in CeO_2_, and the two oxygen atoms of CO_2_ with two adjacent Lewis acidic Ce^3+^ centres in CeO_2_. This CeO_2_ solid material showed FLP-inspired tandem activation of CO_2_ and reactions with alkenes to catalytically form selective cyclic carbonates.^170^ Davide *et al.* reported that CO_2_ activation is shown to occur *via* a bidentate carbonate bridging the FLP through a Ce^3+^-to-CO_2_ charge transfer (Scheme 68).^171^ The authors performed a detailed study of the system in which an FLP was formed over a highly defective sample of CeO_2_.

The reaction of CO_2_ with MeOH formed monomethylcarbonate through an FLP mechanism involving Ce^3+^ and oxygen vacancies.

Recently, other cerium based FLP systems have also been explored for the reduction of CO_2_ into products such as CH_4_ and carbonates.^172^

Finally, it should be noted that there are several metal organic framework (MOF) systems that have Lewis acidic metal centres and Lewis basic ligands that can also act in an FLP manner to activate CO_2_.^173–175^

## Stoichiometric and catalytic reduction of CO_2_: scope and limitations

So far, we have witnessed a wealth of FLP systems for CO_2_ activation and reduction to different products. In this final section we summarise the key findings of the different FLP systems described in terms of CO_2_ activation and the stability of the CO_2_ adducts, as well as the CO_2_ reduction strategies using hydrogen, silane or borane reducing agents.

### An insight into the stability of CO_2_ adducts

In this review many different FLP adducts with CO_2_ are discussed. Stability of the FLP-CO_2_ adducts is dependent on different factors, such as the state of the system (solid or solution phase), temperature and conditions (*e.g.* such as applying vacuum), the strength of the Lewis base-C (CO_2_) and Lewis acid-O (CO_2_) bonds, steric effects around the ligand attached to the acidic or basic reactive centre, and the geometry of the FLP system either as intra- or intermolecular systems. Several systems undergo reversible CO_2_ activation which is highly dependent upon the geometry of the FLP (intra- or intermolecular) as well as the electronic and steric effects at the Lewis acid and basic sites. Among the described examples of the FLP systems for CO_2_ activation, the first was described for the B/P intra- and intermolecular systems. The FLP *t*Bu_3_P/B(C_6_F_5_)_3_ with CO_2_ was observed to form a stable adduct *t*Bu_3_P-CO_2_-B(C_6_F_5_)_3_ at room temperature but, upon heating under vacuum releases CO_2_ and regenerates the FLP. The same outcome was observed for intramolecular system 2, which produces a cyclic adduct with CO_2_ with lower stability which decomposes even at −20 °C.^34^ When the Lewis base and acid are aligned in a geminal fashion, an increase in reactivity is observed as seen in the formation of adduct 10 with a non-fluorinated FLP.^42^ A unique binding mode of CO_2_ in the FLP system bis-borane was observed that resulted in compound 9 as a six-membered stable adduct in which two boron Lewis acidic centres in the FLP bind to the two oxygen atoms in the CO_2_ molecule. This, however, was not the case for the FLP *t*Bu_3_P/O(B(C_6_F_5_)_2_)_2_, where chelation of CO_2_ by two B-centres was not observed due to steric effects and as well as a significant π-character in the B–O bonds supported by crystal structure information.^41^ With the more Lewis acidic and oxophilic aluminium Lewis acids, more stable CO_2_ adducts were typically observed and in several cases both oxygen atoms of CO_2_ bound to a Lewis acidic site, through coordination to two aluminium centres for example in compounds 74–76 and 78.^103,111^ The binding strength could be tuned by varying the substituents on aluminium. The more Lewis acidic centres –AlCl_2_ and –Al(C_6_F_5_)_2_ bind CO_2_ irreversibly, while less Lewis acidic –AlMeCl binds CO_2_ reversibly under 2 bar CO_2_, liberating CO_2_ when the excess pressure is released. For catalytic applications, this reversible binding is necessary to enable release of the product. In several cases, not only CO_2_ activation is observed but also further reactivity of the adduct with the FLP to generate more stable CO_2_ activated products. These reactions, however, would be irreversible and therefore stoichiometric. Examples of this have been observed in the B/N FLP 34 in which a cyclohexyl group migrates from boron to an adjacent carbon centre.^72^ Overall, in the activation of CO_2_ molecule specially in the homogeneous FLP-CO_2_ adduct systems, a species that can reversibly form weak adducts of CO_2_ with almost no energy barrier in either direction would be an incredible valuable tool to enable catalytic transformations. Alternatively, combinations of LA and LB are promising that show reversible CO_2_ binding. Homogeneous and heterogenous metal based FLP systems have been shown to be very promising in the activation of CO_2_ through an FLP mechanism and offer much promise for catalytic turnover (see later).

### CO_2_ reductions in FLP systems

**Fig. 14:**
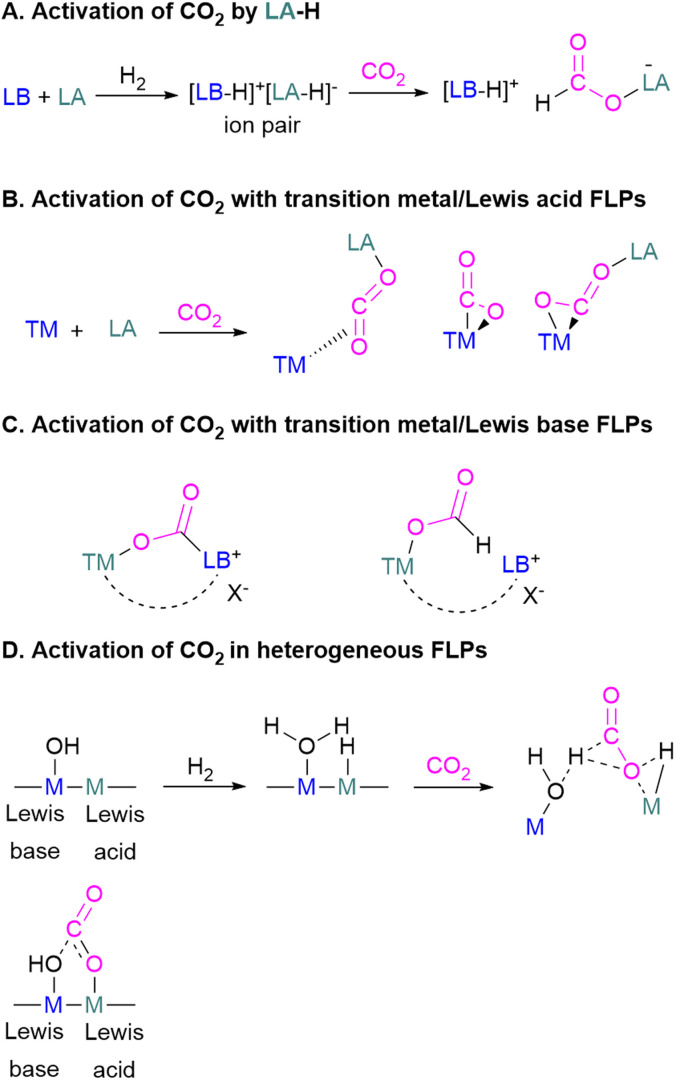
CO_2_ activation mode in different FLP systems.

As we have seen in this review, the binding and activation modes of small molecules (in this case CO_2_ and H_2_) are different in homogeneous (metals and non-metal in inter- and intramolecular) and heterogeneous FLP systems. In homogeneous systems, the combination of Lewis base and acid in frustrated pairs typically cleave the H–H bond heterolytically and form ion pairs [LB–H]^+^[LA–H]^−^. [LA–H]^−^ acts as a hydride source and can reduce CO_2_ by transferring H^−^ to the carbon atom. The oxygen anion generated is then trapped by the Lewis acid generating formates (Fig. 14A). The formation of formate salts of CO_2_ with H_2_ surrogates [LB–H]^+^[LA–H]^−^ are shown as examples in Schemes 12, 24 and 25. To utilise this strategy for CO_2_ reduction in a catalytic way has been studied computationally showing a possible reduction of CO_2_ to HCO_2_H in Schemes 15 and 16 but practically has not been demonstrated. For a practical feasibility, the ion pair [LB–H]^+^ should be able to supply H^+^ to the formed formate ion [HCOO–LA]^−^. If this occurs, then the FLP would catalyse hydrogenation of CO_2_, but the limiting factor is the release of the formate from [HCOO–LA]^−^ due to the strength of the O–LA bond. In other words, for a catalytic hydrogenation of CO_2_ using FLPs, the ion pair [LB–H]^+^[LA–H]^−^ should regenerate the free Lewis acid and base following CO_2_ reduction. This remains a key challenge in main group FLP-CO_2_ reduction and the use of a strong Lewis acid often precludes product release. One strategy to overcome this could be to use inverse frustrated Lewis pair systems. This was observed for the use of excess Lewis base DBU in combination with the Lewis acid tbtb (tris(*p*-bromo)tridurylborane), an inverse FLP as shown in Scheme 31.^83^ Another example of catalytic hydrogenation of CO_2_ to formate was explored by using K_2_CO_3_/B(C_6_F_5_)_3_ with H_2_.^102^

Metal systems as a Lewis basic centre in combination with a Lewis acid show a different way of activating CO_2_ and coordinate to the CO_2_ molecule through the C=O bond (Fig. 14B) as seen with Lewis basic Pt, Re and Mo systems. The Lewis acidic component of the FLP then activates the oxygen atom. CO_2_ can be found in a reduced state with a Re system where reduction to formate is observed through CO_2_ hydrogenation using H_2_. When the transition metal is incorporated as the Lewis acid component of an FLP, then the mode of CO_2_ activation is similar to that observed for the main group Lewis acids with TM–O bond formation (Fig. 14C). Here, it is interesting to note that the system before reaction with CO_2_ can cleave H_2_ heterolytically, similar to other FLPs and the TM–H bond then inserts into the CO_2_ molecule. An example for this type of reaction is shown for cationic zirconocene–phosphinoaryloxide complexes in Scheme 56.

Heterogeneous FLPs systems activate CO_2_ using a similar concept but *via* a different mechanism (Fig. 14D) and are generally not limited by some of the challenges that main group systems face. In these systems the surface has Lewis acidic metal sites such as indium or titanium. However, the Lewis basic sites are typically an oxygen atom (often as a hydroxy group). This tolerance to hydroxy functional groups is a significant advantage in the heterogenous systems and provides easier routes for CO_2_ reduction. In many of the main group FLP systems described herein the strong binding to oxygen centres, while beneficial for CO_2_ activation, is detrimental to catalytic turnover.

### CO_2_ reduction by FLPs: using H_2_, activated B–H and Si–H

Several catalytic reduction methods have been developed utilising different FLP systems using different reducing agents. For CO_2_ reduction, direct hydrogenation provides the best approach as it is clean and generates no waste. However, many FLP systems, especially those using the main group elements, have used other reducing agents such as silanes or boranes due to the inability of the system to activate H_2_ in conjunction with CO_2_. Thus, although some FLPs showed promising results for the CO_2_ reduction with a direct use of H_2_ gas as a reducing agent, heterogeneous systems have shown to be more promising with the direct use of H_2_. As described by several computational studies considering hydrogenation of CO_2_ with only H_2_ as the reducing agent, the energy barrier to the activation of H_2_ by the FLP system is generally higher than the activation barrier for that of CO_2_. Fine-tuning of both the Lewis acid's ability to accept a hydride from H_2_ and the Lewis base's ability to accept a proton from H_2_ is necessary to consequently reduce activated CO_2_. The computational work herein have highlighted the importance for a cumulative high Lewis basicity and acidity, whilst avoiding the combination of a very strong Lewis acid with a very strong Lewis base as this negatively impacts both the activation barriers to H_2_ and CO_2_.

**Fig. 15:**
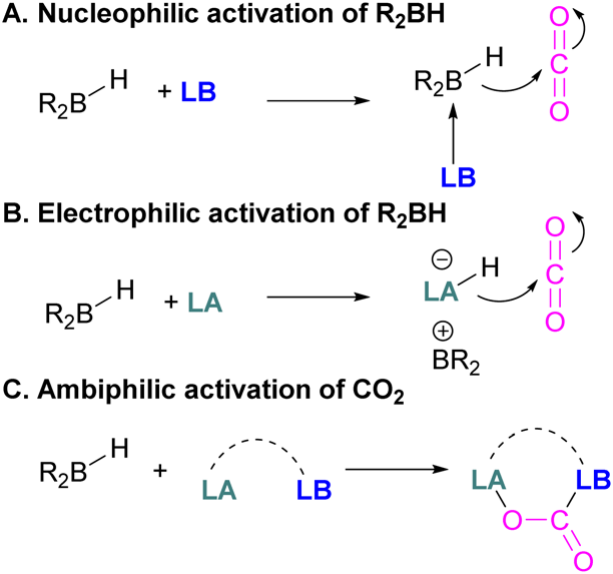
Activation of boranes in CO_2_ reduction.

For many systems seen in this review, boron reducing agents have commonly been employed in homogeneous systems, for example R_2_BH/HBpin. Here either the Lewis base or acid activates the reducing agent towards reduction, as shown in Fig. 15. The first mode of CO_2_ reduction with R_2_BH and the Lewis base is the nucleophilic activation of R_2_BH. Strong nucleophiles favor hydride transfer to CO_2_ by increasing the hydridicity of the B–H bond (Fig. 15A). Alternatively, the Lewis acids may abstract the hydride of the B–H bond to yield a boron electrophile and convert the Lewis acid catalyst to a strong hydride donor (Fig. 15B).^176^ In these two mechanisms, the Lewis acid component is then trapped by the generated oxygen anion. In these cases, the formation of stable CO_2_ adducts often hampers catalysis by stabilising the catalyst's resting state. As we have seen above, the FLP catalyst can also activate the CO_2_ molecule directly. As CO_2_ is a weak Lewis base, this mode of action usually involves a bifunctional activation of CO_2_ with the cooperative effect of a Lewis base and a Lewis acid (Fig. 15C). The borohydride reducing agent then can directly hydrogenate this species. In many cases, “activation” of CO_2_ in the form of an adduct is both deleterious and necessary to the catalytic activity, as it stabilises the lowest intermediate in the potential energy surface yet prepares CO_2_ for the subsequent reduction steps by removing electron density from the carbon by coordination to the Lewis acid. It is noteworthy that future catalytic systems based on this approach should be target compounds that show a lower affinity for CO_2_, based on thermodynamics, yet increase the electrophilicity of the carbon centre. Instead of borane reducing agents, silanes such as Et_3_SiH have also been used for catalytic CO_2_ reduction. For example, using R_3_SiH with a Lewis acid, an electrophilic activation is observed similar to the way shown Fig. 15B. Although several examples use H_2_ as the reducing agent, this remains a challenge with main group systems and more success has been observed in this regard when using either homogenous or heterogenous transition metal systems.

### Stoichiometric and catalytic reduction of CO_2_ in FLPs

**Fig. 16:**
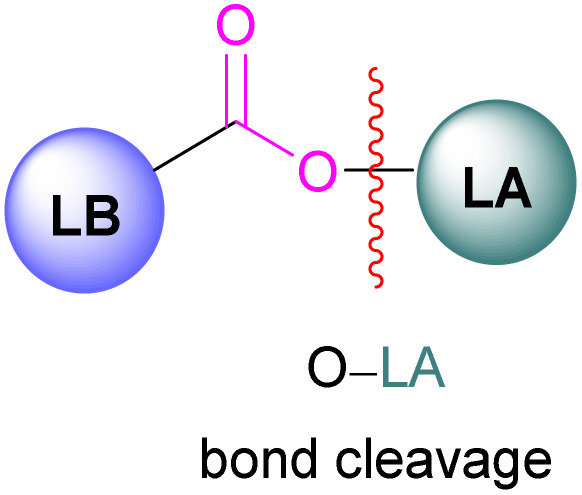
Mode for facile reduction of CO_2_.

Various homogeneous and heterogeneous FLPs systems have been investigated for the reduction of CO_2_. It depends on certain properties of the FLP system to guide the CO_2_ reduction either for a stoichiometric or a catalytic reaction pathway. For CO_2_ adducts, a strong Lewis base and a weak Lewis acid adduct of CO_2_ appear suitable for catalytic CO_2_ reduction whereas stronger Lewis acids form stable CO_2_ adducts. To carry out a catalytic reduction of CO_2_ in a zwitterionic system, a first condition is that the bond between O atom of CO_2_ and the Lewis acid should be weak, *i.e.* the Lewis acid should not be too acidic or oxophilic (see Fig. 16). Secondly, the cation of the activated reducing agents (R_2_B–H or R_3_Si–H) after supplying hydride ion should be able to trap the oxygen atom, cleaving the O–LA bond. Here, both the Lewis acid and base are not coordinated, and the system will be able to reversibly activate and reduce CO_2_ leading to a catalytic pathway. Several CO_2_ adduct systems have been discussed for catalytic CO_2_ reductions. Intramolecular FLP 21 acts as an efficient catalyst because it does not form an adduct with CO_2_ (as most of the FLPs form stable CO_2_ adducts), and the CH_2_O moiety is released upon reduction from the catalyst and thus makes 21 active for another turnover.^55–57^ In the *t*Bu_3_P/9-BBN FLP system, hydride transfer from boron to the carbonyl carbon releases *t*Bu_3_P for the next cycle, and hence this system also works catalytically.^58^ Another interesting catalytic example is for the TMP/B(C_6_F_5_)_3_ FLP system, where Et_3_SiH is employed as a silane reducing agent producing Et_3_Si^+^ following B(C_6_F_5_)_3_ Si–H activation.^78^ Et_3_Si^+^ is a good oxygen acceptor and thus promotes the catalytic deoxygenation of CO_2_ to CH_4_ and (Et_3_Si)_2_O. Although catalytic, the stoichiometric use of silane is not desirable in the longer term and routes that employ H_2_ as a hydrogen source should be sought. Making use of FLPs in direct catalytic hydrogenations of CO_2_ remain difficult. Although hydride transfer from [LA–H] to CO_2_ have been described in this review, the proton transfer from [LB–H] does not occur readily due to the formation of a strong O–LA bond (Fig. 16). Hence, most FLP systems are seen to terminate at adduct formation as O–LA, and although the H_2_ activation barrier may have been overcome, full hydrogenation of CO_2_ is prohibited. Some success has been achieved with inverse FLP systems (*e.g.* tris(*p*-bromo)tridurylborane (tbtb)/DBU).^83^ To obtain a suitable FLP for the direct catalytic hydrogenation of CO_2_, the intrinsic reactivity of each of the components is very important *i.e.*, free energy of proton attachment to the Lewis base and free energy of hydride attachment to the Lewis acid. For high turnover numbers and turnover frequencies, a catalyst should be stable enough and should regenerate in the catalytic cycle. In some of the examples, the highest in carbene system 58 using 9-BBN as the reducing agent, FLPs have shown a good TON and TOF for the reduction of CO_2_. As shown in the latter part of this review, metals and heterogeneous FLPs are generally efficient to activate and use H_2_ as a direct reducing source for CO_2_. For example, indium oxide has been found to be a particularly good example of a heterogeneous FLP system where H_2_ is directly utilised for the reduction of CO_2_.^159^ The potential of transition metals is that they provide reactive sites for the activation of H_2_ as well as CO_2_, but in several cases, they are not cost efficient.

## Conclusions

Tremendous progress in CO_2_ activation and reduction to value added products in both homogeneous and heterogeneous systems on bench scale has been seen. Both homogenous and heterogenous transition metal systems, as Lewis acids and bases have been efficient for the reduction of CO_2_. However, main group elements have emerged as alternatives and remarkable progress has been made here. Group 13 elements, boron and aluminium as Lewis acids have been heavily explored combined with Lewis bases such as phosphines, amines, or carbenes. In many cases, activation of CO_2_ has been achieved up to full conversion under mild conditions, and in several examples reduced products can be obtained in the form of methanol, formates, acetates and CH_4_ upon addition of a silane or borane, or in some cases H_2_. Most commonly, the formation of a zwitterionic product is the initial key activation step in the reduction of CO_2_. However, subsequent product liberation has been seen to be the most limiting step in these reactions, thus limiting the scope of catalytically viable reactions. Currently H_2_ is utilised as a reducing agent on industrial scale as it is cheap and widely available, but its use in CO_2_ reduction with FLPs is limited and other reducing agents such as hydrosilanes, hydroboranes or ammonia boranes are often used. However, a drawback of these reducing agents in CO_2_ reduction is that they form strong Si–O and B–O bonds and form oxidised products such as siloxanes or boroxanes, making the process less atom economic. Thus, there still need to be significant development of FLP CO_2_ reduction using H_2_ as the reducing agent. Compared to main group FLP-CO_2_ adduct systems, transition metals possessing empty orbitals that offer site selective coordination have been applied as more suitable systems to activate the non-polar covalent bond in H_2_ and utilise this as a direct reducing source. Conversely, heterogeneous systems provide the opportunity of recycling and have also been seen to achieve higher TONs and TOFs. Present chemical methods of recycling siloxanes or boroxanes to the corresponding hydrosilanes or hydroboranes are energy intense. Electrochemical methods are efficient and have made material recycling possible. Therefore, electrochemical methods may be an attractive and efficient approach over chemical routes for recycling of the oxides to their corresponding hydrides. The key to obtain high TON for hydrosilylation, hydroboration, or hydrogenation relies on the stability of the catalyst, the release of the reduced products and then catalyst regeneration. Thermodynamic control is the main limitation in accessing CO_2_ reduced products beyond carboxylates, whilst kinetic control is the main limitation in achieving high output catalytic cycles that regenerate the catalyst. Although some progress has been made to achieve CO_2_ reduction in a catalytic manner, the challenge is still to achieve a robust and effective FLP system that can catalyse CO_2_ reduction at ambient temperature and pressure utilising H_2_ as a reducing agent. In addition, there must be more focus on selective reduction reactions to give valuable products that are of interest to industry. Further investigations should focus on developing highly active and selective FLP systems for the catalytic conversion to C_1_ or C_2_ products, and could include various methods for conversion such as thermal, photochemical, or electrochemical methods.

## Author contributions

All authors contributed to the writing and revisions of the review.

## Conflicts of interest

There are no conflicts to declare.

## Supplementary Material
